# Antibacterial Spirotetronate Polyketides from an *Actinomadura* sp. Strain A30804

**DOI:** 10.3390/molecules27238196

**Published:** 2022-11-24

**Authors:** Kuan-Chieh Ching, Elaine J. Chin, Mario Wibowo, Zann Y. Tan, Lay-Kien Yang, Deborah C. Seow, Chung-Yan Leong, Veronica W. Ng, Siew-Bee Ng, Yoganathan Kanagasundaram

**Affiliations:** Singapore Institute of Food and Biotechnology Innovation (SIFBI), Agency for Science, Technology and Research (A*STAR), Singapore 138673, Singapore

**Keywords:** spirotetronate, decatromicin, pyrrolosporin, antibacterial, natural products, structure elucidation, *Actinomadura* sp.

## Abstract

Large scale cultivation and chemical investigation of an extract obtained from *Actimonadura* sp. resulted in the identification of six previously undescribed spirotetronates (pyrrolosporin B and decatromicins C–G; **7**–**12**), along with six known congeners, namely decatromicins A–B (**1**–**2**), BE-45722B–D (**3**–**5**), and pyrrolosporin A (**6**). The chemical structures of compounds **1**–**12** were characterized via comparison with previously reported data and analysis of 1D/2D NMR and MS data. The structures of all new compounds were highly related to the spirotetronate type compounds, decatromicin and pyrrolosporin, with variations in the substituents on the pyrrole and aglycone moieties. All compounds were evaluated for antibacterial activity against the Gram-negative bacteria, *Acinetobacter baumannii* and Gram-positive bacteria, *Staphylococcus aureus* and were investigated for their cytotoxicity against the human cancer cell line A549. Of these, decatromicin B (**2**), BE-45722B (**3**), and pyrrolosporin B (**7**) exhibited potent antibacterial activities against both Gram-positive (MIC_90_ between 1–3 μM) and Gram-negative bacteria (MIC_90_ values ranging from 12–36 μM) with weak or no cytotoxic activity against A549 cells.

## 1. Introduction

Antimicrobial resistance is one of the leading threats to human health globally [1]. Recently, it was estimated that 1.2 million people died from antibiotic-resistant bacterial infections, which was more than that caused by HIV/AIDS or malaria [2]. Thus, there is clearly an urgent need for new and effective antimicrobials. For many years, our group has engaged in a screening effort to detect secondary metabolites from Nature that can inhibit pathogenic microorganism [3,4,5,6], such as *Staphylococcus aureus*. Notably, *S. aureus* is one of the leading pathogens (the second after *Escherichia coli*) for fatalities associated with resistance [2]. Microbes have been one of the most prolific sources of small molecules where two third of known microbial secondary metabolites are derived from actinobacteria [7,8]. Actinobacteria is a large group of morphologically and physiologically diverse bacteria well known for their production of natural products and biotechnologically relevant compounds [9]. It is known that 70% of them are produced by the genus *Streptomyces*. Among the non-*Streptomyces* species, *Actinomadura* has been reported as a producer of chemically and biologically unique polyketides possessing antitumor, antimicrobial and anticoccidial activities. Up to date, several spirotetronate-class polyketides have been isolated from *Actinomadura*. For instance, nomimicin, a polyketide isolated from an *Actinomadura* strain TP-A0878 in a compost sample collected at Nomi, Ishikawa, Japan. Subsequently nomimicins B-D, new tetronate-class polyketides were also isolated from a marine-derived actinomycete of the genus *Actinomadura*. These compounds showed antimicrobial activities [10,11].

**Figure 1:**
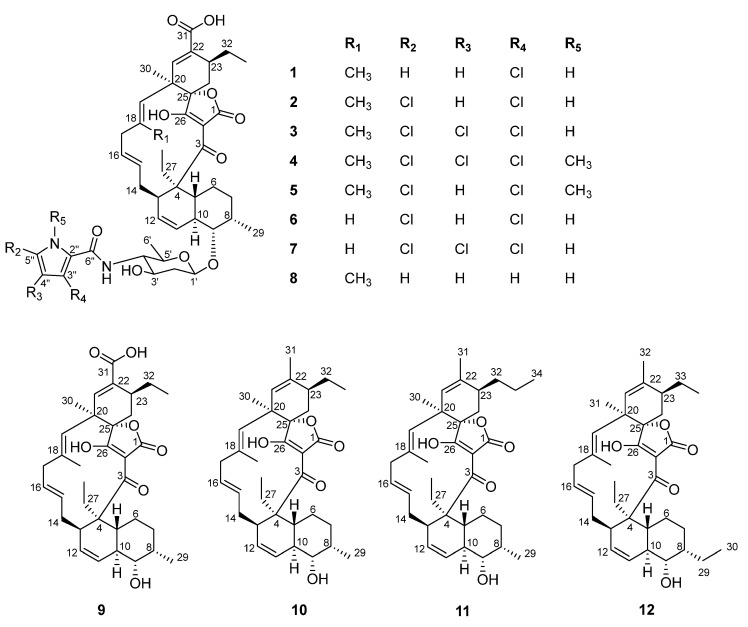
Chemical structures of compounds **1**–**12**.

During our screening assays, a few MeOH extracts from our in-house Actinobacteria strains library [12] exhibited antibacterial activity against *S. aureus*. Chemical dereplication of the active extracts suggested the presence of several spirotetronate polyketides, a group of compounds that was known for their significant pharmacological potentials [13]. The spirotetronate polyketides feature an unusual aglycone core containing a typical tetronic acid spiro-connected to a six-membered ring and linked to a *trans*-decalin moiety. Further chemical analysis of the active extracts shortlisted one extract from *Actinomadura* sp. strain A30804 that produced higher yields of potentially new spirotetronate analogs for large-scale cultivation. Bioassay guided purification of the extract yielded twelve spirotetronate natural products, including six known compounds, decatromicin A–B (**1**–**2**) [14,15], BE-45722B–D (**3**–**5**) [16,17,18], and pyrrolosporin A (**6**) [19,20], as well as six new analogs, pyrrolosporin B (**7**) and decatromicin C–G (**8**–**12**) (Figure 1). In this report, we describe the purification and structure elucidation of these natural products and further demonstrate the antimicrobial potential of the spirotetronates. Moreover, the discovery of these structurally intricate compounds enriches the chemical diversity of the spirotetronates and potentially leads to a better understanding of preliminary structure–activity relationships.

## 2. Materials and Methods

### 2.1. General Experimental Procedures

Specific rotations were recorded using JASCO P-2000 digital polarimeter. Bruker DRX-400 NMR spectrometer with cryoprobe was used to record NMR spectra. The NMR spectrometer was equipped with 5 mm BBI (^1^H, COSY, edited HSQC, and HMBC) or BBO (^13^C) probe head with *z*-gradients. The ^1^H and ^13^C NMR chemical shifts were referenced to the residual solvent peaks for MeOH-*d*_4_ at δ_H_ 3.31 and δ_C_ 49.0 ppm; or DMSO-*d*_6_ at δ_H_ 2.50 and δ_C_ 39.5 ppm. Preparative RP-HPLC was performed using XTerra MS C_18_ Prep column (19 × 300 mm, 10 µm) on an Agilent 1260 Infinity Preparative-Scale LCMS Purification System hyphenated with Agilent 6130B single quadrupole MS as a detector. LCMS data were recorded using Agilent UHPLC 1290 Infinity coupled to Agilent 6540 accurate-mass quadrupole time-of-flight (QTOF)-ESIMS. Standard gradient conditions of 98% H_2_O (0.1% FA) to 100% CH_3_CN (0.1% FA) were run over 8.6 min using an Acquity UPLC BEH C_18_ (2.1 × 50 mm, 1.7 µm) column, all at a flow rate of 0.5 mL/min. The QTOF were set using the same parameter as previously reported [4].

### 2.2. Molecular Identification and Phylogenetic Analysis of the Bacteria Isolate A30804

The DNA of bacterial strain A30804 was extracted from the plate using the DNeasy PowerSoil Pro Kit (Qiagen, Hilden, Germany) according to the manufacturer’s protocol. Upon extraction, DNA purity and yield of A30804 was measured with NanoDrop2000 spectroscopy system (ThermoFisher Scientific, Waltham, MA, USA). Bacterial 16S rRNA genes of interest were amplified from the extracted DNA using the universal 16S primers 27F (5′—AGA GTT TGA TCC TGG CTC AG—3′) and 1492R (5′—TAC GGY TAC CTT GTT ACG ACT T—3′) [21,22]. The PCR amplification reactions were performed using Applied Biosystems ProFlex Thermocycler (ThermoFisher Scientific, Waltham, MA, USA) with a total reaction volume of 20 µL that comprised of 2.0 µL of 10× PCR buffer with 20 mM MgCl_2_, 2.0 µL of 2 mM dNTPs, 1 unit of Taq polymerase (ThermoFisher Scientific, USA), 1.0 µL of 10 µM of each primer and 1.0 μL of purified DNA templates. The thermal cycling profile used was, 5 min initial denaturation at 95 °C; further denaturation at 95 °C (30 cycles, 30 s each), annealing at 60 °C for 50 s, followed by 1 min extension at 72 °C, and a final extension at 72 °C for 5 min. A negative control and non-template were included in the run. This was followed by Sanger Sequencing of the PCR amplified DNA fragments (1st BASE, Singapore). Alignment of the sequences were carried out using Benchling and followed by analysis using BLAST [National Center for Biotechnology Information (NCBI)]. A30804 was aligned using ClustalW with respective closely related actinobacteria strains obtained from the GenBank databases with I6S rRNA region. Neighbor-joining tree algorithm method was used to establish the genetic relationship between the strains. The phylogenetic tree was constructed with a bootstrapped database containing 1000 replicates in MEGA 11.0 software (Mega, US). The I6S rRNA gene sequence of strain A30804 has been deposited in GenBank database of NCBI under the accession number OP225395.

### 2.3. Fermentation and Extraction of Bacterial Crude Extract

*Actinomadura* sp. strain A30804 were cultured in 5 mL SV2 media, (For 1 L, add 15 g glucose (1st BASE, Singapore), 15 g glycerol (VWR, Radnor, PA, USA), 15 g soya peptone (Oxoid, Basingstoke, Hampshire, UK), and 1 g calcium carbonate (Sigma Aldrich, St. Louis, MO, USA), pH adjusted to 7.0) for 3 days at 28 °C under constant shaking at 200 rpm to generate a seed culture. The seed culture was inoculated into fresh CA09LB media (10 g meat extract (Sigma-Aldrich, St. Louis, MO, USA), 4 g yeast extract (BD Biosciences, Franklin Lakes, NJ, USA), 20 g glucose (1st BASE, Singapore), and glycerol 3 g (VWR, Radnor, PA, USA) in 1 L of H_2_O, pH adjusted to 7.0) in a 1:20 volume ratio and incubated at 28 °C in the dark with shaking at 200 rpm. After 9 days of incubation, the cultures were centrifuged to separate biomass and supernatant, followed by lyophilization. The dried cultures were extracted by MeOH and filtered through filter paper (Whatman Grade 4, Maidstone, Kent, UK). MeOH was removed under reduced pressure to give a crude extract of a combined weight of 40.02 g.

### 2.4. Isolation and Structure Elucidation

The dried extracts obtained were combined and partitioned with CH_2_Cl_2_/MeOH/H_2_O in a ratio of 2:1:1. The CH_2_Cl_2_ layer was then evaporated to dryness under reduced pressure using a rotary evaporator. The CH_2_Cl_2_ crude extract (2.1 g) was subjected to a Si gel column chromatography (2.5 x 22 cm) using a stepwise gradient solvent system from 100% CH_2_Cl_2_ to 12% MeOH/CH_2_Cl_2_ (2% MeOH increment, 250 mL each), followed by a final 100% MeOH elution to afford eight fractions (A1–A8). Fractions A4–A6 (509.9 mg) were combined and further separated by C_18_ RP-HPLC. Isocratic conditions of 50% MeCN/H_2_O) (0.1% FA) were initially held for 5 min, followed by a linear gradient to 90% MeCN/H_2_O (0.1% FA) over 55 min, a gradient to 100% MeCN (0.1% FA) over 2 min, and isocratic conditions at 100% MeCN for 10 min, all at a flow rate of 24 mL/min. This purification step yielded decatromicin B (**2**, 6.5 mg) and BE-45722B (**3**, 3.4 mg). Fractions A7 and A8 were pooled (356.6 mg) and separated by C_18_ RP-HPLC. Isocratic conditions of 50% MeCN/H_2_O) (0.1% FA) were initially held for 5 min, followed by a linear gradient to 80% MeCN/H_2_O (0.1% FA) over 45 min, a gradient to 100% MeCN (0.1% FA) over 2 min, and isocratic elution at 100% MeCN for 10 min, all at a flow rate of 24 mL/min to afford compound **8** (7.5 mg), decatromicin A (**1**, 10.9 mg), pyrrolosporin A (**6**, 3.1 mg) and compound **7** (3.8 mg). Fraction A2 (257.7 mg) was further separated using C_18_ RP-HPLC. Initially, isocratic conditions of 50% MeCN/H_2_O) (0.1% FA) were held for 5 min, followed by a linear gradient to 100% MeCN (0.1% FA) over 45 min, then isocratic conditions at 100% MeCN for 10 min, all at a flow rate of 24 mL/min to yield BE-45722D (**5**, 17.1 mg), BE-45722C (**4**, 16.8 mg), compound **9** (2.1 mg), compound **10** (4.1 mg), and a subfraction containing a mixture of **11** and **12**. The mixture (3.5 mg) of **11** and **12** was further separated using preparative TLC with a solvent system of 0.5% MeOH/CH_2_Cl_2_ to obtain pure compounds **11** (1.2 mg) and **12** (1.0 mg).

### 2.5. Chemical Structural Data

The UV spectra and HRESIMS spectra of **7–12**, and 1D and 2D NMR spectra of **1**–**12** are provided in Supplementary Information, Figures S1, S2 and S4–S55.

Pyrrolosporin B (**7**): White amorphous powders; ${\left[\alpha \right]}_{D}^{23}$−4 (c 0.5, MeOH); UV (MeCN/H_2_O) λmax (%) 220 (100%), 272 (70%) nm; (–)-HRESIMS: *m/z* 873.2706 (84%) [M–H]^−^; 875.2685 (100%) [M–H]^−^ (calcd for C_44_H_52_N_2_O_10_Cl_3_, 873.2693); ^1^H and ^13^C NMR data, see Table 1.

Decatromicin C (**8**): White amorphous powders; ${\left[\alpha \right]}_{D}^{23}$ + 39 (c 1.0, MeOH); UV (MeCN/H_2_O) λmax (%) 216 (100%), 270 (70%) nm; (–)-HRESIMS: *m/z* 785.4034 [M–H]^−^ (calcd for C_45_H_57_N_2_O_10_, 785.4019); ^1^H and ^13^C NMR data, see Table 1.

Decatromicin D (**9**): White amorphous powders; ${\left[\alpha \right]}_{D}^{23}$ + 133 (c 0.2, MeOH); UV (MeCN/H_2_O) λmax (%) 216 (100%), 272 (28%) nm; (–)-HRESIMS: *m/z* 563.3015 [M–H]^−^ (calcd for C_34_H_43_O_7_, 563.3014); ^1^H and ^13^C NMR data, see Table 2.

Decatromicin E (**10**): White amorphous powders; ${\left[\alpha \right]}_{D}^{23}$ + 86 (c 0.4, MeOH); UV (MeCN/H_2_O) λmax (%) 216 (100%), 272 (44%) nm; (–)-HRESIMS: *m/z* 533.3275 [M–H]^−^ (calcd for C_34_H_45_O_5_, 533.3272); ^1^H and ^13^C NMR data, see Table 2.

Decatromicin F (**11**): White amorphous powders; ${\left[\alpha \right]}_{D}^{23}$ + 35 (c 0.2, MeOH); UV (MeCN/H_2_O) λmax (%) 224 (100%), 277 (38%) nm; (–)-HRESIMS: *m/z* 547.3443 [M-H]- (calcd for C_35_H_48_O_5_, 547.3429); ^1^H and ^13^C NMR data, see Table 2.

Decatromicin G (**12**): White amorphous powders; ${\left[\alpha \right]}_{D}^{23}$ + 90 (c 0.1, MeOH); UV (MeCN/H_2_O) λmax (%) 224 (100%), 377 (38%) nm; (–)-HRESIMS: *m/z* 547.3438 [M-H]- (calcd for C_35_H_48_O_5_, 547.3429); ^1^H and ^13^C NMR data, see Table 2.

**Table 2:** ^1^H (400 MHz) and ^13^C (100 MHz) NMR data of decatromicins D– G (**9–12**) in MeOH-*d*_4_.

Pos.	9		10		11		12	
	^13^C	^1^H, Mult. (*J* = Hz)	^13^C	^1^H, Mult. (*J* = Hz)	^13^C	^1^H, Mult. (*J* = Hz)	^13^C	^1^H, Mult. (*J* = Hz)
1	167.7, C	–	169.8, C	–	*	–	168.7,C	–
2	102.5, C	–	104.3, C	–	103.6	–	*	–
3	203.0, C	–	206.1, C	–	*	–	205.1, C	–
4	55.8, C	–	56.1, C	–	56.0, C	–	56.0, C	–
5	41.8, CH	1.79, m	41.4, CH	1.81, m	41.6, CH	1.80, m	41.5, CH	1.82, m
6	23.9, CH_2_	1.32, m; 1.80, m	23.9, CH_2_	1.38, m; 1.81, m	23.9, CH_2_	1.37, m; 1.82, m	23.9, CH_2_	1.31, m; 1.84, m
7	33.4, CH_2_	1.63 (2H), m;	33.3, CH_2_	1.65 (2H), m	33.4, CH_2_	1.65 (2H), m	33.1, CH_2_	1.54 (2H), m
8	36.2, CH	2.12, m	36.1, CH	2.14, m	36.1, CH	2.12, m	44.1, CH	1.77, m
9	77.5, CH	3.41, dd (5.2, 10.6)	77.2, CH	3.42, dd (5.2, 10.6)	77.3, CH	3.42, dd (5.1, 10.7)	77.7, CH	3.44, dd (4.8, 10.4)
10	40.8, CH	2.05, m	40.6, CH	2.08, t (10.7)	40.7, CH	2.06, t (10.2)	41.4, CH	2.01, m
11	125.5, CH	5.79, d (9.9)	125.7, CH	5.82, d (9.6)	125.6, CH	5.80, d (10.2)	125.7, CH	5.83, d (10.3)
12	133.0, CH	5.65, m	132.1, CH	5.67, ddd (2.3, 5.8, 10.1)	132.7, CH	5.66, ddd (2.4, 6.0, 9.9)	132.4, CH	5.65, ddd (2.3, 5.7, 9.9)
13	43.3, CH	2.98, m	44.1, CH	2.90, br q (5.3)	43.9, CH	2.96, m	43.9, CH	2.92, m
14	38.1, CH_2_	1.91, m; 2.00, m	38.0, CH_2_	1.99 (2H), m	38.0, CH_2_	1.96 (2H), m	38.0, CH_2_	1.97 (2H), m
15	132.7, CH	5.24, m	132.3, CH	5.17, m	132.7, CH	5.21, m	132.4, CH	5.19, m
16	128.7, CH	5.40, m	129.3, CH	5.41, m	129.1, CH	5.39, m	129.4, CH	5.38, m
17	45.7, CH_2_	2.34, m; 2.46, dd (9.3, 12.4)	46.0, CH_2_	2.34, m; 2.44, m	45.9, CH_2_	2.33, m; 2.42, m	46.0, CH_2_	2.33, m; 2.43, m
18	139.5, C	–	139.3, C		139.0, C		139.1, C	
19	127.3, CH	4.99, br s	128.0, CH	4.94, br s	128.5, CH	4.95, br s	128.3, CH	4.94, br s
20	44.0, C	–	43.5, C		43.6, C	–	43.6, C	–
21	144.7, CH	7.03, d (1.1)	128.8, CH	5.57, br t (1.4)	129.1, C	5.55, br s	129.2, C	5.56, br t (1.7)
22	133.0, C	–	134.9, C		134.7, C	–	134.8, C	–
23	37.5, CH	2.64, m	42.0, CH	2.17, m	39.7, CH	2.27, m	42.1, CH	2.15, m
24	30.7, CH_2_	1.79, m; 2.35, m	31.5, CH_2_	1.75, m; 2.46, m	35.9, CH_2_	1.69, m; 2.42, m	31.4, CH_2_	1.74, m; 2.40, m
25	86.3, C	–	87.8, C	–	87.6, C	–	87.6, C	–
26	200.6, C	–	200.8, C	–	201.1, C	–	200.9, C	–
27	24.1, CH_2_	1.83, m; 2.75, q (7.5)	24.1, CH_2_	1.89, m; 2.72, m	24.1, CH_2_	1.85, m; 2.72, m	24.1, CH_2_	1.80, m; 2.72, m
28	13.3, CH_3_	0.93, t (7.5)	12.2, CH_3_	0.90, t (7.5)	12.4, CH_3_	0.91, t (7.6)	12.4, CH_3_	0.90, m
29	12.7, CH_3_	1.00, d (7.0)	12.6, CH_3_	1.00, d (7.0)	12.6, CH_3_	1.00, d (6.9)	18.4, CH_2_	1.76, m
30	27.0, CH_3_	1.27, s	27.6, CH_3_	1.26, s	27.4, CH_3_	1.25, s	13.0, CH_3_	0.92, m
31	171.4, C	–	22.2, CH_3_	1.79, s	22.3, CH_3_	1.77, s	27.5, CH_3_	1.25, s
32	27.3, CH_2_	1.72 (2H), m	26.2, CH_2_	1.53, dq (7.2, 10.5); 1.79, m	26.2, CH_2_	1.61 (2H), m	22.3, CH_3_	1.78, s
33	12.5, CH_3_	0.91, t (7.6)	13.3, CH_3_	0.91, t (7.4)	23.7, CH_2_	1.34 (2H), m	26.2, CH_2_	1.60, m; 1.77, m
34	–	–	–	–	14.3, CH_3_	0.91, t (7.6)	13.4, CH_3_	0.91, m
18-CH_3_	19.1, CH_3_	1.76, s	18.6, CH_3_	1.77, s	18.6, CH_3_	1.77, s	18.6, CH_3_	1.77, s

* Not detected due to minute amounts.

### 2.6. Biological Assays

Antimicrobial effect of compounds **1**–**12** were tested against a panel of Gram-negative bacteria, including *Acinetobacter baumannii* (ATCC^®^ 19606™), *Pseudomonas aeruginosa* (ATCC^®^ 9027™), and *Klebsiella aerogenes* (ATCC^®^ 13048™), the Gram-positive *Staphylococcus aureus* Rosenbach (ATCC^®^ 25923™) and the fungal strain *Aspergillus fumigatus* (ATCC^®^ 46645™). The minimum inhibitory concentration (MIC) and minimum bactericidal/fungicidal concentration (MBC/MFC) determination were carried out using the microbroth dilution method, based on the Clinical Laboratory Standards Institute (CLSI) guidelines, with minor modifications. Antibacterial assays were tested with cells seeded at 5.5 × 10^5^ cells/mL, whereas the antifungal assay was carried out at 2.5 × 10^4^ spores/mL. The tested compounds were incubated together with the bacterial cells at 37 °C for 24 h, and at 25 °C for 72 h for the fungal spores for MIC testing. OD_600_ measurement was subsequentially performed on the plates to determine the inhibitory effect of the compounds. MBC/MFC was determined by transferring 5 µL of the treated culture into new media microplates, where the plates were incubated under the same condition, followed by OD_600_ measurement. Isolated compounds **1–12** were also tested against A549 human lung carcinoma cells (ATCC^®^ CCL-185™) for cytotoxicity assessment, where cells were seeded at 3.3 × 10^4^ cells/mL. Following that, compounds were added to the cells and further incubated for 72 h at 37 °C in the presence of 5% CO_2_. The cytotoxic effect of the compounds was detected using PrestoBlue™ cell viability reagent (ThermoFisher Scientific, Waltham, MA, USA). After the addition of the reagent, cells were further incubated for 2 h before fluorescence measurement at excitation 560 nm and emission 590 nm. All assays were performed in triplicates to ensure reproducibility. Standard inhibitors gentamicin (Gibco, Waltham, MA, USA) was used as the assay control for antibacterial, amphotericin (Sigma Aldrich, St. Louis, MO, USA) for antifungal and puromycin (Sigma Aldrich, St. Louis, MO, USA) for cytotoxicity testing. The dose–response curves for IC_90_ and IC_50_ values determination were plotted using the GraphPad Prism 8 software (GraphPad, San Diego, CA, USA).

## 3. Results and Discussion

**Figure 2:**
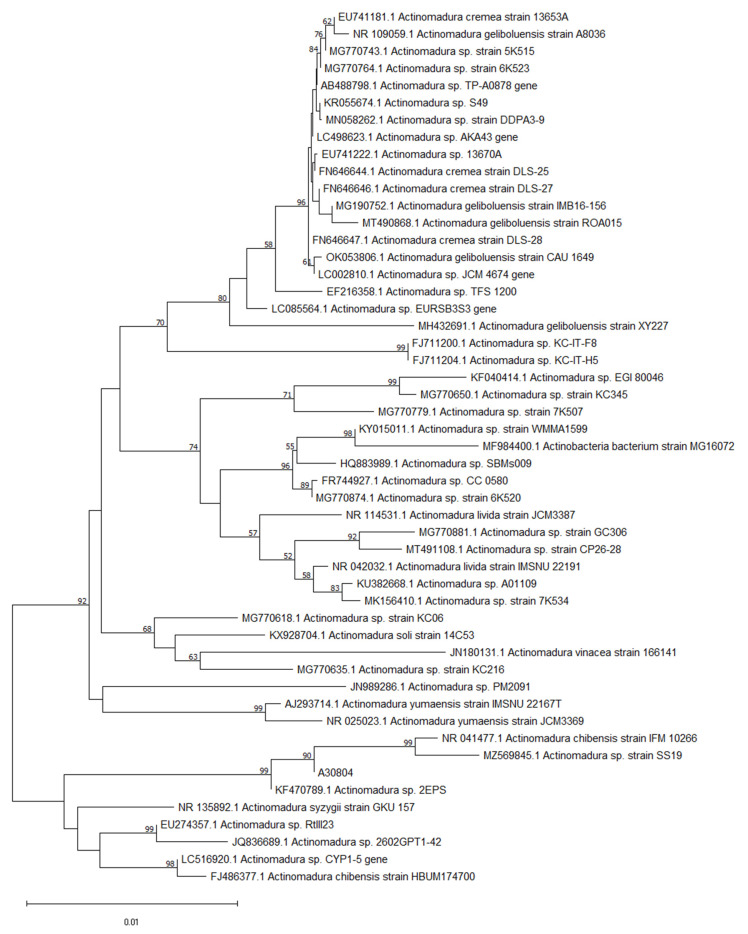
Phylogenetic tree showing the evolutionary relationship between A30804 and other type species of the genus *Actinomadura*. Neighbor-joining phylogenetic tree was constructed based on 16S rRNA gene sequence showing the relationship between isolated strain A30804 and representatives or related actinobacteria strains retrieved from the GenBank with their respective accession numbers. Bootstrap values greater than 50% are shown at the number on the branches nodes which were analyzed based on 1000 replicates. Bar, 0.01 substitutions per nucleotide position.

**Figure 3:**
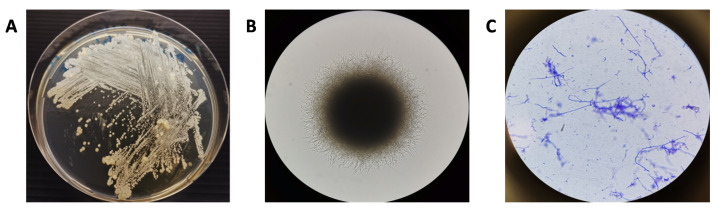
Visual images of the strain *Actinomadura* sp. (**A**) Macroscopic plate image. (**B**) Colony morphology on agar plate (100× magnification). (**C**) Cell morphology by Gram stain on glass slide (1000× magnification).

Molecular identification was used to identify the closely related species of interest to the isolated strain A30804. The sequence obtained from the 16S rRNA gene sequence of A30804 was aligned and further analyzed where a nucleotide BLAST search was performed against the NCBI 16S ribosomal RNA database. The phylogenetic relatedness using the neighbor-joining analysis method of isolated strain and its closely related species obtained from the Genbank database is shown in Figure 2. Phylogenetic analysis based on 16S rRNA gene sequences revealed that strain A30804, accession number OP225395 belonged to the genus *Actinomadura* (Figure 3). The isolate formed a subcluster with *Actinomadura* sp. 2EPS, *Actinomadura chibensis* strain IFM 10266 and *Actinomadura* sp. strain SS19 recovered by neighbor-joining analysis. Previous studies have shown that *Actinomadura* sp. have been reported to produce spirotetronate polyketides compounds such as decatromicin A and B [15,17].

The MeOH extract obtained from the liquid culture of *Actinomadura* sp. was separated by various chromatographic techniques to afford six known and six previously undescribed spirotetronate polyketides, pyrrolosporin B (**7**), decatromicin C–G (**8**–**12**) (Figure 1). The known spirotetronates, decatromicin A–B (**1**–**2**), BE-45722B–D (**3**–**5**), and pyrrolosporin A (**6**) were characterized by comparisons of their MS data, ^1^H and ^13^C NMR spectra to published data.

**Figure 4:**
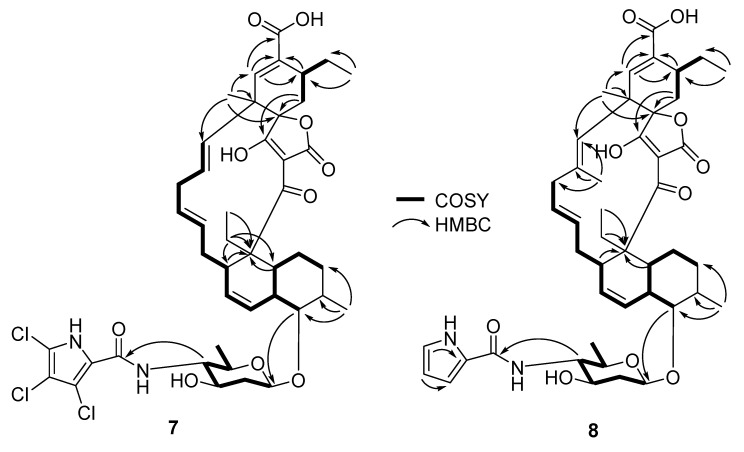
Selected HMBC and COSY correlations for **7** and **8**.

**Figure 5:**
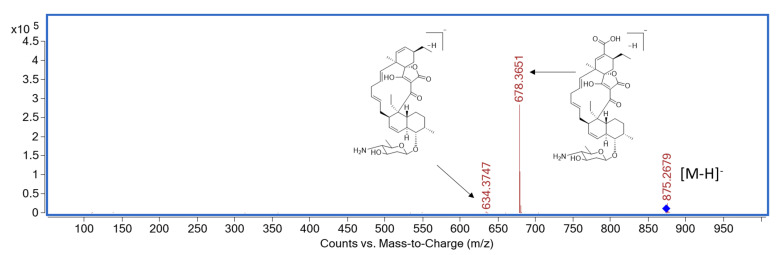
MS/MS spectrum for **7**.

**Figure 6:**
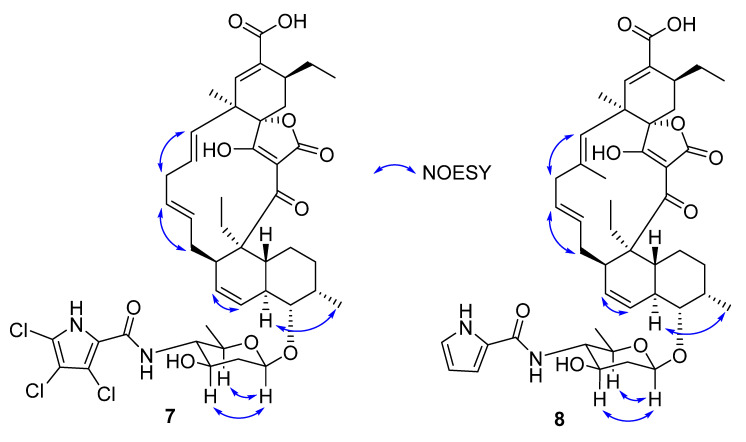
Selected NOESY correlations for **7** and **8**.

Compound **7**, white amorphous powders, had a molecular formula of C_44_H_53_C_l3_N_2_O_10_, as revealed by (–)-HRESIMS. The ^1^H, ^13^C, and HSQC NMR data (Table 1) indicated the presence of five methyl, eight methylene, seventeen methine, and fourteen non-protonated carbons. Detailed analysis of 2D NMR spectra suggested that **7** belonged to the spirotetronate polyketide structural class (Figure 4). For example, ^1^H-^1^H COSY spectrum readily assigned the fragment H-5 along the chain to H-19; HMBC cross-peaks from H_3_-30 to C-19, C-21, and C-25, together with HMBC correlations from H-21 to C-22, C-23, and C-31 as well as ^1^H-^1^H COSY between H-23 and H_2_-24 and HMBC correlation from H-24 to C-25 allowed the identification of a cyclohexene ring (linked to C-19) decorated with a carboxylic acid at C-22. ^1^H-^1^H COSY between H_2_-32 and H-23 and HMBC correlation from H-33 to C-23 established an ethyl fragment attached to C-23. Further HMBC correlations from H_2_-27 to C-4, C-5, and C-13; and H_3_-28 to C-4 assigned the position of CH_3_-CH_2_- fragment on C-4. The presence of a sugar moiety attached to C-9 was evident by ^1^H-^1^H COSY between H-1′/H_2_-2′/H-3′/H-4′/H-5′/H_3_-6′, and HMBC correlation from H-9 to an anomeric carbon at δ_C_ 102.7 ppm. The features of the ^1^H and ^13^C spectra of **7** were very similar to those for pyrrolosporin A (**6**) (Supplementary Information Table S3), except that the -CH resonance from the pyrrole moiety in **6** was absent in **7**. Analysis of MS data and the isotopic pattern of the molecular ion peak suggested the presence of three chlorine atoms in **7**. These observations together with MS data analysis revealed that **7** had a unique trichlorinated pyrrole moiety. Consistently, MS/MS fragmentation analysis showed a fragment with an *m/z* value of 687.3651, which suggested a loss of the trichlorinated pyrrole carbonyl fragment (Figure 5) [23]. Similar coupling constant values and NOESY correlations in **7** to those of pyrrolosporin A (**6**) indicated the same relative configurations, for instance, a coupling constant of 8.8 Hz between H--1′ and H--2′ax readily indicated a β-glycosidic linkage between the sugar and the backbone of the molecule, a coupling constant of 10.2 Hz between H-9 and H-10 suggested a *trans*-diaxial relationship. NOESY correlations between H-10 and H_3_-29, H-1′ and H-3′ and H-1′ and H-5′ further supported the relative configuration as drawn on Figure 1 and Figure 6. Thus, the structure of **7** was established as a new member of the spirotetronate polyketide structural class and named pyrrolosporin B (**7**).

Compound **8**, white amorphous powders, had a molecular formula of C_45_H_58_N_2_O_10_ assigned based on (–)-HRESIMS data. Comparison of ^1^H NMR data of **8** with those of **7** showed an additional singlet methyl at δ_H_ 1.79 and three additional proton resonances at δ_H_ 6.16, 6.82, and 6.90, characteristic ^1^H resonances of a pyrrole moiety (Table 1). The additional methyl was positioned at C-18 based on HMBC correlations from δ_H_ 1.79 to C-17, C-18, and C-19 (Figure 4). The other features of ^1^H and ^13^C of **8** were similar to those of decatromicin A (**1**). Based on these observations, compound **8** was a dechlorinated version of decatromicin A (**1**). The relative configurations of **8** were deemed to be the same as in **7** following analyses of *J*-coupling and NOESY data (Figure 6). Thus, the structure of **8** was assigned and named decatromicin C. It is worth mentioning that compound **8** was previously obtained from a dechlorination reaction of **1** using tri-*n*-butylstannane [14]. However, spectroscopic data of **8** was not reported. We have also included HRMS and full NMR spectroscopic data of **8**, which were not reported in the original paper. This is the first report of the identification of **8** from a natural source.

**Figure 7:**
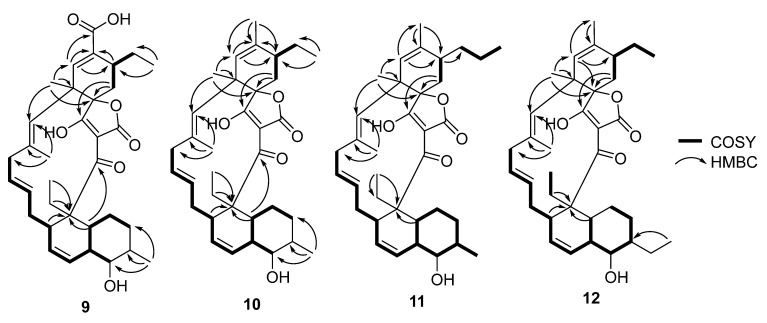
Selected COSY, HMBC correlations for **9**–**12**.

**Figure 8:**
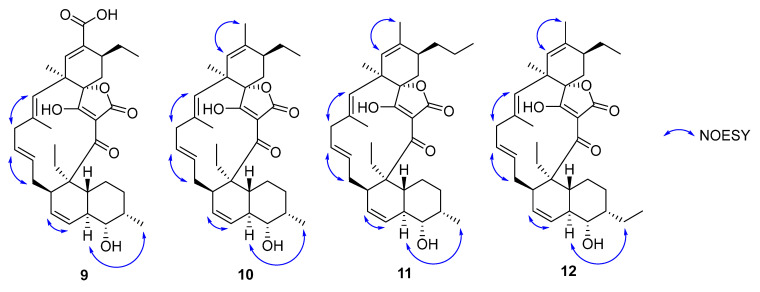
Selected NOESY correlations for **9**-**12**.

Compound **9** had a molecular formula of C_34_H_44_O_7_ following HRMS data analysis. ^1^H and ^13^C NMR spectra of **9** were similar to those of **8**. However, the ^1^H NMR signals for the pyrrole group and amino sugar in **8** were missing in **9** (Table 2). Furthermore, the ^1^H NMR resonance of H-9 was shifted downfield from δ_H_ 3.39 in **8** to δ_H_ 3.41 in **9**. Detailed 2D NMR (Figure 7) data analysis indicated that **9** was the aglycone core of **8**, and named decatromicin D. The relative configuration of **9** was deemed to be identical as **8** following NOESY data interpretation (Figure 8). Previously, aglycone **9** was obtained from a degradation study of decatromicin A (**1**) [14]. Here, we report the isolation and identification of **9** from a natural source.

Compound **10**, white amorphous powders, was assigned the molecular formula C_34_H_46_O_5_. The ^1^H and ^13^C NMR spectra of **10** were almost identical to those of **9**, indicating that compound **10** had the same spirotetronate aglycone core structure. ^1^H NMR data showed that compound **10** had an extra methyl compared to **9** (Table 2). The ^13^C carbonyl NMR resonance at δ_C_ 171.4 in **9** was not observed in **10**, suggesting that the carboxylic acid group in **9** was replaced by a methyl group. This was supported by HMBC correlations from the singlet methyl at δ_H_ 1.79 to C-21, C-22, and C-23 (Figure 7). The same relative configuration previously assigned for **9** was also determined for **10** following analyses of the NOESY data and ^1^H-^1^H coupling constants (Figure 8). Thus, the structure of **10** was established and named decatromicin E.

The molecular formula C_35_H_48_O_5_ was assigned for compound **11** following (–)-HRESIMS spectrum analysis. MS and NMR data comparison between **10** and **11** revealed that the latter compound had an additional -CH_2_- moiety (Table 2). ^1^H-^1^H COSY spectrum established a fragment of H_2_-32/H_2_-33/H-34 which was attached to C-23 since HMBC correlation from H-23 to C-32 was observed (Figure 7). The relative configuration of **11** was judged to be identical to **10** following the NOESY data comparison (Figure 8). Therefore, the structure of **11** was elucidated and named decatromicin F.

The minor compound **12** had the same molecular formula C_35_H_48_O_5_ as that of **11**, indicating that these compounds were structural isomers. However, the methyl doublet at δ_H_ 1.00 in **11** was absent in **12**. This observation together with HMBC correlation from H_3_-30 to C-8 located an ethyl group on C-8 (Figure 7). ^1^H-^1^H coupling constants and the NOESY spectrum analysis assigned the relative configurations in **12** to be the same as in **10** (Figure 8). Hence, the structure of **12** was elucidated and named decatromicin G. Due to the minute amount of decatromicin F (**11**) and G (**12**), several of ^13^C NMR resonances for these molecules were not observed in the ^13^C NMR spectra (Table 2). However, their ^1^H/2D NMR, UV, and MS spectra were comparable with other isolated compounds (**1**–**10**), indicating they belonged to spirotetronate polyketide type of compounds. 

During the isolation of compounds **7**–**12**, six known spirotetronate polyketides, including decatromicin A–B (**1**–**2**), BE-45722B–D (**3**–**5**), and pyrrolosporin A (**6**) were also isolated and identified. Decatromicins A (**1**) and B (**2**) were initially discovered from *Actinomadura* sp. MK73-NF4 and showed potent antimicrobial activity against Gram-positive methicillin-resistant *Staphylococcus aureus* (MRSA) [14,15], while BE-45722 series of compounds (**3**–**5**) were analogs of the decatromicins with an altered decoration of chlorine on their pyrrole group [16,17]. Compounds **3**–**5** were also reported to demonstrate antibacterial activity against a panel of Gram-positive bacteria, including *Bacillus cereus*, *Bacillus subtilis*, *S. aureus*, and *Clostridium perfringens*. Another congener, pyrrolosporin A (**6**), was first isolated from *Micromonospora* sp. (ATCC 53791) [19,20]. The main structural difference between pyrrolosporin A (**6**) and decatromicins/BE-45722 series was the absence of a methyl group attached to an olefin in the aglycone core in **6**.

**Figure 9:**
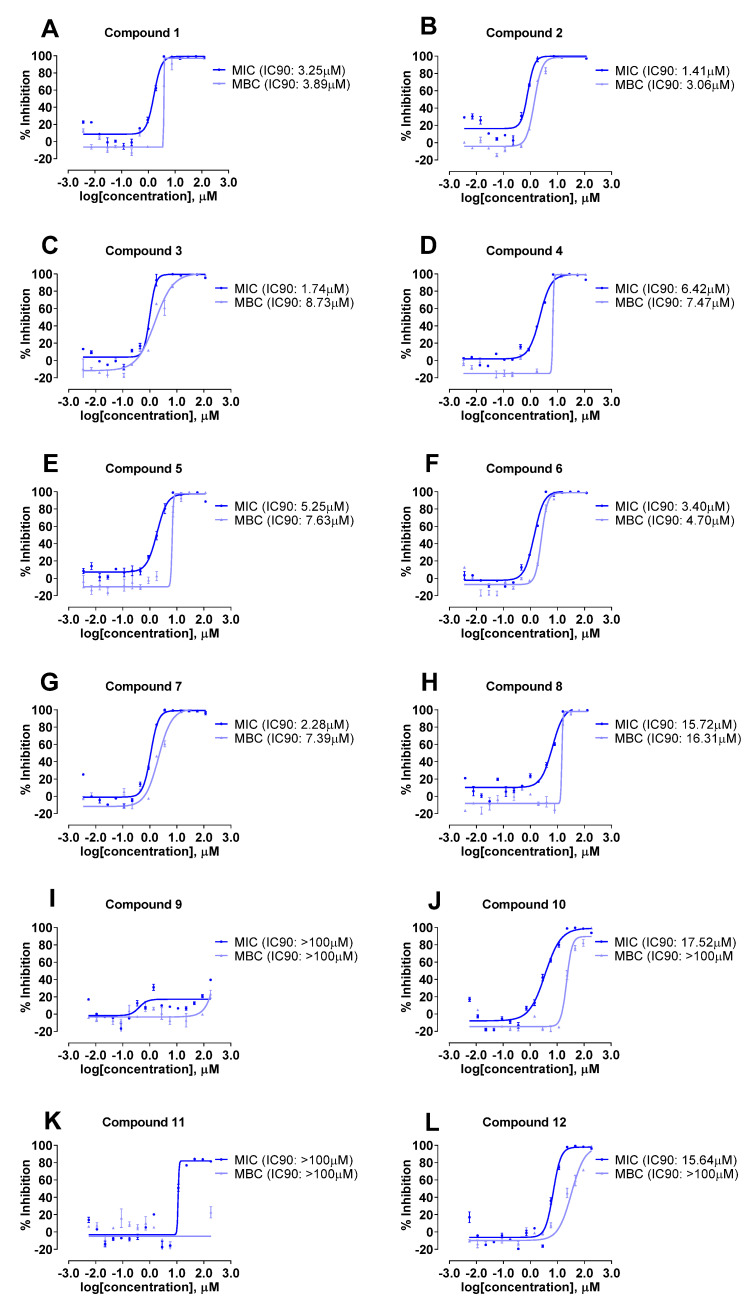
Inhibitory effect dose–response curve against *S. aureus* Rosenbach (ATCC^®^ 25923™) for compounds **1**–**12** (**A**–**L**).

**Figure 10:**
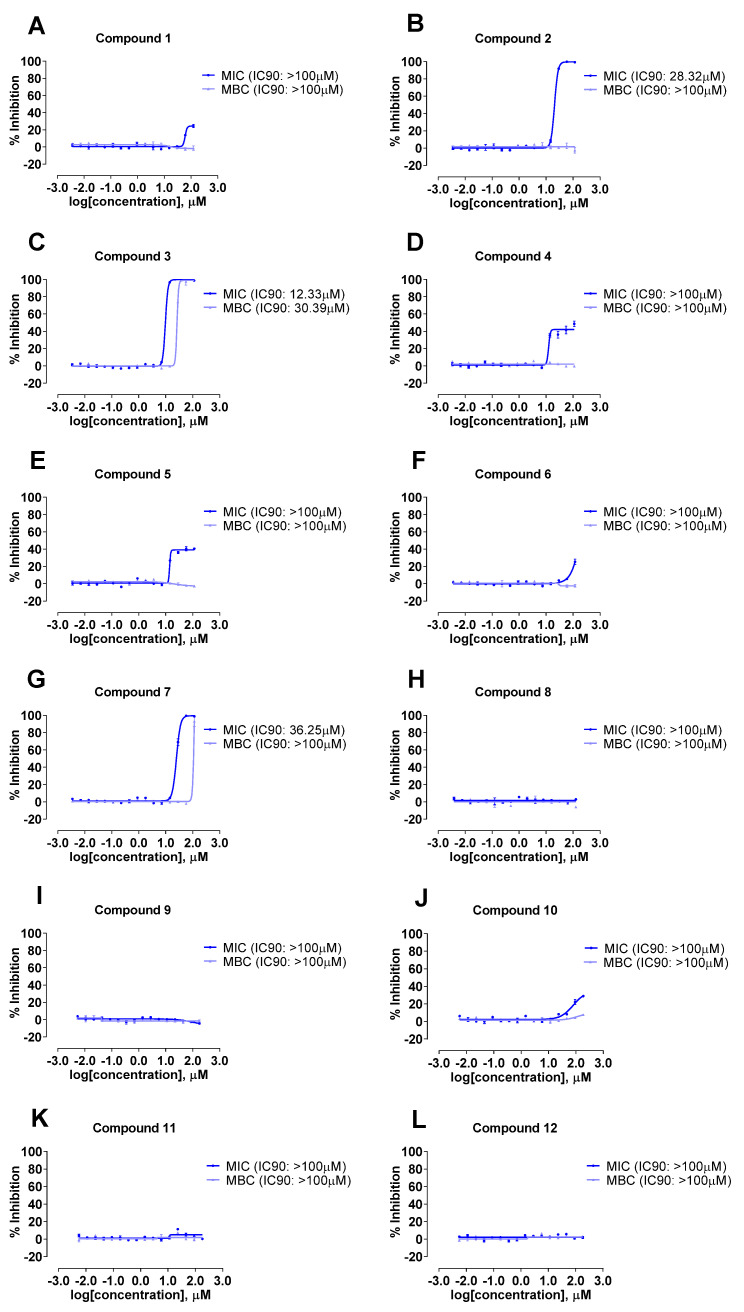
Inhibitory effect dose–response curve against *A. baumannii* (ATCC^®^ 19606) for compounds **1**–**12** (**A**–**L**).

The antibacterial activity of spirotetronate polyketides **1**–**12** was evaluated against a panel of bacterial strains, namely *A. baumannii*, *K. aerogenes*, *P. aeruginosa*, and *S. aureus* Rosenbach. All compounds were inactive against the Gram-negative bacteria *P. aeruginosa* and *K. aerogenes* (Figure S56). Compounds **1**–**8**, **10**, **12** demonstrated activities against *S. aureus* Rosenbach, the only Gram-positive bacteria strain tested (Table 3 and Figure 9). Interestingly, compounds **2**, **3** and **7** exhibited inhibitory activity against Gram negative strain *A. baumannii* (Table 3 and Figure 10), which was not reported previously for this class of compounds. In addition, the antifungal activities of **1**–**12** was also evaluated against *A. fumigatus;* no antifungal activity was observed (Figure S57). Furthermore, all the compounds were investigated for their cytotoxicity against the human lung carcinoma cell line; and compounds **3**–**5** showed weak cytotoxic activity towards A549 cells (Figure S58). Notably, it has been well documented that decatromicin compounds exhibited a wide range of biological properties; and are strong antibiotics against Gram-positive bacteria, including contemporary strains of methicillin-resistant *Staphylococcus aureus* (MRSA) [14,15].

Compounds **1**–**5** and **8** possess the same aglycone core structure. In this series of compounds, at least two chloro substitutions and a free NH on the pyrrole moiety are important for antibacterial activity against *A. baumannii* as seen in compounds **2** and **3** (i.e., MIC_90_ between 12 μM and 30 μM). In addition, compound **3** was 2-fold more active against *A. baumannii* (MIC_90_ of 12.3 µM and MBC_90_ of 30.4 µM) as compared to compound **2** (i.e., MIC_90_ of 28.3 µM and MBC_90_ >100 µM) as the number of chloro substituent on the pyrrole moiety increases (Table 3). 

Similar observation was made in compounds **6** and **7** which possess the same aglyone structure. For instance, compound **7** which was trichloro substituted at the pyrrole moiety showed antibacterial activity against *A. baumannii* (i.e., MIC_90_ of 36.3 μM) while compound **6** (i.e., dichloro substituted) was inactive against *A. baumannii*. Moreover, a 3-fold increase in antibacterial activity was observed in compound **3** as compared to compound **7** when the C-18 position in the aglycone moiety was substituted with a methyl group (Table 3).

Compounds **1**-**8**, **10**, **12** were active against *S. aureus* Rosenbach. Among these compounds, compounds **2**, **3** and **7** were the most potent (MIC_90_ between 1 μM and 3 μM). Our studies showed that the potency of these compounds against *S. aureus* Rosenbach was likely attributed to at least two chloro- substitutions on the pyrrole moiety. On the other hand, a decrease in antibacterial activity against *S. aureus* Rosenbach was observed in compounds **4** and **5** (i.e., MIC_90_ between 5 μM and 7 μM) when NH group in pyrrole moiety was substituted with a methyl group even though their pyrrole moieties were substituted with at least two chlorine. In addition, the absence of any chlorine and methyl substitutions on the pyrrole moiety in compound **8** led to 3 to 10-fold decrease in antibacterial activity against *S. aureus* Rosenbach (i.e., MIC_90_ of 15.7 µM) as compared to compounds **2**–**5**. 

Interestingly, a replacement of the carboxylic acid group on the C-22 position in the aglycone moiety in compound **9** with a methyl group in compound **10** led to antibacterial activity against *S. aureus* Rosenbach as shown in Table 3 and Figure 9 (i.e., **9**: MIC_90_ > 100 µM; **10**: MIC_90_ of 17.5 µM). This could be due to the poor cell membrane permeability of the carboxylic acid group, thus resulting in no antibacterial activity in compound **9**. 

For compounds **10** and **11**, a replacement of the ethyl group with a propyl group on the C-23 position in the aglycone moiety resulted in weaker activity against *S. aureus* Rosenbach with an MIC_90_ value of 17.5 µM in **10**, whilst **11** did not exhibit any antibacterial activity. In addition, a replacement of the methyl group with an ethyl group on the C-8 position in the aglycone moiety as seen in compounds **10** and **12,** respectively**,** did not lead to any significant change in activity against *S. aureus* Rosenbach, with a MIC_90_ value of 15.6 µM in compound **12**. Of all the compounds tested, compounds **2** and **3** (decatromicin B and BE-45722B, respectively) and **7** (pyrrolosporin B) showed the most potent antibacterial activities against both Gram-negative and Gram-positive bacteria, as well as weak or no cytotoxic activity against A549 cells.

## 4. Conclusions

*Actinomadura* sp. strain A30804 was found to produce spirotetronate polyketides **1**-**12**. Compounds **2** and **3** (decatromicin B and BE-45722B, respectively) and **7** (pyrrolosporin B) not only exhibited weak to no cytotoxicity to laboratory human cell line A549, but also demonstrated potent antibacterial activity against both Gram-positive bacteria, *S. aureus* Rosenbach (ATCC^®^ 25923™) with MIC_90_ values ranging from 1 µM to 3 µM and Gram-negative bacteria, *A. baumannii* (ATCC^®^ 19606™) with MIC_90_ values ranging from 12 µM to 36 µM. Preliminary structure–activity relationship studies showed the presence of at least two chloro- substitutions on the pyrrole moieties was important for antibacterial activity in this series of spirotetronate compounds. This report further supported the potential of these spirotetronate polyketides as potent antibacterial agents. 

## Figures and Tables

**Table 1:** ^1^H (400 MHz) and ^13^C (100 MHz) NMR data of decatromicins C (**7**) and D (**8**) in MeOH-*d*_4_.

Pos.	7		8	
	^13^C, Type	^1^H, Mult. (*J* = Hz)	^13^C, Type	^1^H, Mult. (*J* = Hz)
1	164.7, C	–	169.5, C	–
2	104.5, C	–	104.0, C	–
3	206.2, C	–	205.5, C	–
4	56.1, C	–	56.0, C	–
5	41.9, CH	1.82, m	41.7, CH	1.84, m
6	23.9, CH_2_	1.37, m; 1.80, m	24.0, CH_2_	1.38, m; 1.81, m
7	33.3, CH_2_	1.59 (2H), m	33.2, CH_2_	1.63 (2H), m
8	35.6, CH	2.34, m	35.4, CH	2.40, m
9	87.3, CH	3.37, dd (5.3, 10.2)	87.4, CH	3.39, m
10	40.0, CH	2.15, t (10.7)	39.7, CH	2.17, t (10.6)
11	125.4, CH	5.59, m	125.5, CH	5.68, t (10.4)
12	132.8, CH	5.69, ddd (2.2, 5.9, 10.1)	132.6, CH	5.70, ddd (1.4, 5.2, 9.9)
13	44.8, CH	2.94, m	43.9, CH	2.90, br dd (4.5, 9.1)
14	37.8, CH_2_	1.95 (2H), m	38.0, CH_2_	2.02 (2H), m
15	129.0, CH	5.30, m	132.5, CH	5.21, m
16	129.0, CH	5.35, m	129.1, CH	5.43, ddd (5.3, 9.5, 14.9)
17	35.2, CH_2_	2.53 (2H), m	45.7, CH_2_	2.37, dd (4.8, 12.3); 2.50, dd (9.0, 12.0)
18	132.3, CH	5.63, dd (8.1, 16.1)	140.2, C	–
19	134.2, CH	5.41, d (15.9)	126.8, CH	5.00, br s
20	43.3, C	–	44.0, C	–
21	143.9, CH	6.76, d (1.8)	143.9, CH	7.06, d (1.5)
22	133.8, C	–	132.8, C	–
23	37.5, CH	2.65, m	37.2, CH	2.70, m
24	31.5, CH_2_	1.83, m; 2.46, m	31.0, CH_2_	1.83, m; 2.47, m
25	86.7, C	–	86.9, C	–
26	200.4, C	–	200.4, C	–
27	24.2, CH_2_	1.82, m; 2.74, m	24.1, CH_2_	1.87, m; 2.73, m
28	12.4, CH_3_	0.94, t (7.5)	12.2, CH_3_	0.92, t (7.2)
29	13.5, CH_3_	1.01, d (7.0)	13.5, CH_3_	1.04, d (7.0)
30	24.4, CH_3_	1.28, s	26.9, CH_3_	1.31, s
31	170.6, C	–	170.5	–
32	27.6, CH_2_	1.53, m; 1.65, m	27.3, CH_2_	1.60, m; 1.75, m
33	13.6, CH_3_	0.91, t (7.2)	13.2, CH_3_	0.92, t (7.2)
1′	102.7, CH	4.47, d (8.8)	103.0, CH	4.52, dd (1.3, 9.6)
2′	40.7, CH_2_	1.56, m; 2.22, m	41.2, CH_2_	1.59, m; 2.27, ddd (1.3, 4.6, 12.4)
3′	70.0, CH	3.72, dt (4.9, 11.2)	70.3, CH	3.71, ddd (4.9, 9.8, 11.3)
4′	59.8, CH	3.61, t (9.5)	59.4, CH	3.57, t (9.8)
5′	72.6, CH	3.44, m	72.5, CH	3.42, m
6′	18.9, CH_3_	1.24, d (6.2)	18.7, CH_3_	1.22, d (6.2)
2″	129.1, C	–	127.0	–
3″	121.9, C	–	122.9, CH	6.90, dd (1.4, 2.5)
4″	110.3, C	–	110.1, CH	6.16, dd (2.6, 3.6)
5″	116.8, C	–	111.7, CH	6.82, dd (1.4, 3.7)
6″	161.1, C	–	164.3, C	–
18-CH_3_	–	–	19.1, CH_3_	1.79, s

**Table 3:** Biological activities of compounds **1**–**12**.

**Target Organism or Cell Line (ATCC^®^ Number)**	**Minimal Inhibitory Concentration, MIC_90_ of Decatromicin Compounds (μM)**											
	**1**	**2**	**3**	**4**	**5**	**6**	**7**	**8**	**9**	**10**	**11**	**12**
*Acinetobacter baumannii* (ATCC^®^ 19606™)	>100	28.3	12.3	>100	>100	>100	36.3	>100	>100	>100	>100	>100
*Staphylococcus aureus* Rosenbach (ATCC^®^ 25923™)	3.3	1.4	1.7	6.4	5.3	3.4	2.3	15.7	>100	17.5	>100	15.6
**Target organism or cell line (ATCC^®^ number)**	**Minimal bactericidal concentration, MBC_90_ of decatromicin compounds (μM)**											
	**1**	**2**	**3**	**4**	**5**	**6**	**7**	**8**	**9**	**10**	**11**	**12**
*Acinetobacter baumannii* (ATCC^®^ 19606™)	>100	>100	30.4	>100	>100	>100	>100	>100	>100	>100	>100	>100
*Staphylococcus aureus* Rosenbach (ATCC^®^ 25923™)	3.9	3.1	8.7	7.5	7.6	4.7	7.4	16.3	>100	>100	>100	>100

## Data Availability

Data is contained within the article or Supplementary Materials.

## Supplementary Materials

The following are available online at https://www.mdpi.com/article/10.3390/molecules27238196/s1, Table S1: ^1^H and ^13^C NMR data of 1 and 2; Table S2: ^1^H NMR data of 3, 4 and 5; Table S3: ^1^H NMR data of 6; Figure S1: UV spectra for compounds 7–12; Figure S2: (–)-HRESIMS spectra for compounds 7–12; Figure S3: Selected COSY, HMBC and NOESY correlations of 1; Figure S4: ^1^H NMR spectrum (MeOH-*d*4, 400 MHz) of 1; Figure S5: ^13^C NMR spectrum (MeOH-*d*4, 100 MHz) of 1; Figure S6: COSY spectrum of 1; Figure S7: NOESY spectrum of 1; Figure S8: HSQC spectrum of 1; Figure S9: HMBC spectrum of 1; Figure S10: ^1^H NMR spectrum (MeOH-*d*4, 400 MHz) of 2; Figure S11: ^13^C NMR spectrum (MeOH-*d*4, 100 MHz) of 2; Figure S12: COSY spectrum of 2; Figure S13: HSQC spectrum of 2; Figure S14: HMBC spectrum of 2; Figure S15: ^1^H NMR spectrum (MeOH-*d*4, 400 MHz) of 3; Figure S16: ^1^H NMR spectrum (MeOH-*d*4, 400 MHz) of 4; Figure S17: ^1^H NMR spectrum (MeOH-d4, 400 MHz) of 5; Figure S18: ^1^H NMR spectrum (DMSO-d6, 400 MHz) of 6; Figure S19: ^1^H NMR spectrum (MeOH-d4, 400 MHz) of 6; Figure S20: ^1^H NMR spectrum (MeOH-d4, 400 MHz) of 7; Figure S21: ^13^C NMR spectrum (MeOH-d4, 100 MHz) of 7; Figure S22: COSY spectrum of 7; Figure S23: NOESY spectrum of 7; Figure S24: HSQC spectrum of 7; Figure S25: HMBC spectrum of 7; Figure S26: ^1^H NMR spectrum (MeOH-d4, 400 MHz) of 8; Figure S27: ^13^C NMR spectrum (MeOH-d4, 100 MHz) of 8; Figure S28: COSY spectrum of 8; Figure S29: NOESY spectrum of 8; Figure S30: HSQC spectrum of 8; Figure S31: HMBC spectrum of 8; Figure S32: ^1^H NMR spectrum (MeOH-d4, 400 MHz) of 9; Figure S33: ^13^C NMR spectrum (MeOH-d4, 100 MHz) of 9; Figure S34: COSY spectrum of 9; Figure S35: NOESY spectrum of 9; Figure S36: HSQC spectrum of 9; Figure S37: HMBC spectrum of 9; Figure S38: ^1^H NMR spectrum (MeOH-d4, 400 MHz) of 10; Figure S39: ^13^C NMR spectrum (MeOH-d4, 100 MHz) of 10; Figure S40: COSY spectrum of 10; Figure S41: NOESY spectrum of 10; Figure S42: HSQC spectrum of 10; Figure S43: HMBC spectrum of 10; Figure S44: ^1^H NMR spectrum (MeOH-d4, 400 MHz) of 11; Figure S45: ^13^C NMR spectrum (MeOH-d4, 100 MHz) of 11; Figure S46: COSY spectrum of 11; Figure S47: NOESY spectrum of 11; Figure S48: HSQC spectrum of 11; Figure S49: HMBC spectrum of 11; Figure S50: ^1^H NMR spectrum (MeOH-d4, 400 MHz) of 12; Figure S51: ^13^C NMR spectrum (MeOH-d4, 100 MHz) of 12; Figure S52: COSY spectrum of 12; Figure S53: NOESY spectrum of 12; Figure S54: HSQC spectrum of 12; Figure S55: HMBC spectrum of 12; Figure S56: Dose–response curve of compounds 1-12 against *Klebsiella aerogenes* (ATCC^®^ 13048™) and *Pseudomonas aeruginosa* (ATCC^®^ 9027™); Figure S57: Dose–response curve of compounds 1-12 against *Aspergillus fumigatus* (ATCC^®^ 46645™); Figure S58: Dose–response curve of compounds 1-12 against A549 Human lung carcinoma cells (ATCC^®^ CCL-185™).

## Sample Availability

Samples of the compounds are not available from the authors.
