# Supplementary material for: Antibacterial Spirotetronate Polyketides from an Actinomadura sp. Strain A30804
Source: Molecules. 2022 Nov 24;27(23):8196. doi: 10.3390/molecules27238196 (PMC9737171; doi:10.3390/molecules27238196)
Supplement: Supplementary file 1 [file molecules-27-08196-s001.zip › molecules-2037079-supplementary.pdf]

Supplementary information for

Antibacterial Spirotetronate Polyketides from an *Actinomadura* sp. Strain A30804

Kuan-Chieh Ching †, Elaine J. Chin †, Mario Wibowo, Zann Y. Tan, Lay-Kien Yang, Deborah C. Seow, Chung-Yan Leong, Veronica W. Ng, Siew-Bee Ng * and Yoganathan Kanagasundaram *

Singapore Institute of Food and Biotechnology Innovation (SIFBI), Agency for Science, Technology and Research (A*STAR), Singapore 138673, Singapore

* Correspondence: ngsb@sifbi.a-star.edu.sg (S.-B.N.); yoganathank@sifbi.a-star.edu.sg (Y.K.)

† These authors contributed equally to this work.

Table S1. ^{1}H and ^{13}C NMR data of **1** and **2**.

Table S2. ^{1}H NMR data of **3**, **4** and **5**.

Table S3. ^{1}H NMR data of **6**.

Figure S1. UV spectra for compounds **1**–**12**.

Figure S2. (-)-HRESIMS spectra for compounds **1**–**12**.

Figure S3. Selected COSY, HMBC and NOESY correlations of **1**.

Figure S4. ^{1}H NMR spectrum (MeOH-d_4, 400 MHz) of **1.**

Figure S5. ^{13}C NMR spectrum (MeOH-d_4, 100 MHz) of **1**.

Figure S6. COSY spectrum of **1**.

Figure S7. NOESY spectrum of **1**.

Figure S8. HSQC spectrum of **1**.

Figure S9. HMBC spectrum of **1**.

Figure S10. ^{1}H NMR spectrum (MeOH-d_4, 400 MHz) of **2.**

Figure S11. ^{13}C NMR spectrum (MeOH-d_4, 100 MHz) of **2**.

Figure S12. COSY spectrum of **2**.

Figure S13. HSQC spectrum of **2**.

Figure S14. HMBC spectrum of **2**.

Figure S15. ^{1}H NMR spectrum (MeOH-d_4, 400 MHz) of **3**.

Figure S16. ^{1}H NMR spectrum (MeOH-d_4, 400 MHz) of **4**.

Figure S17. ^{1}H NMR spectrum (MeOH-d_4, 400 MHz) of **5**.

Figure S18. ^{1}H NMR spectrum (DMSO-d_6, 400 MHz) of **6**.

Figure S19. ^{1}H NMR spectrum (MeOH-d_4, 400 MHz) of **6**.

Figure S20. ^{1}H NMR spectrum (MeOH-d_4, 400 MHz) of **7**.

Figure S21. ^{13}C NMR spectrum (MeOH-d_4, 100 MHz) of **7**.

Figure S22. COSY spectrum of **7**.

Figure S23. NOESY spectrum of **7**.

Figure S24. HSQC spectrum of **7**.

Figure S25. HMBC spectrum of **7**.

Figure S26. ^{1}H NMR spectrum (MeOH-d_4, 400 MHz) of **8.**

Figure S27. ^{13}C NMR spectrum (MeOH-d_4, 100 MHz) of **8.**

Figure S28. COSY spectrum of **8.**

Figure S29. NOESY spectrum of **8**.

Figure S30. HSQC spectrum of **8.**

Figure S31. HMBC spectrum of **8.**

Figure S32. ^{1}H NMR spectrum (MeOH-d_4, 400 MHz) of **9.**

Figure S33. ^{13}C NMR spectrum (MeOH-d_4, 100 MHz) of **9.**

Figure S34. COSY spectrum of **9.**

Figure S35. NOESY spectrum of **9**.

Figure S36. HSQC spectrum of **9.**

Figure S37. HMBC spectrum of **9.**

Figure S38. ^{1}H NMR spectrum (MeOH-d_4, 400 MHz) of **10.**

Figure S39. ^{13}C NMR spectrum (MeOH-d_4, 100 MHz) of **10.**

Figure S40. COSY spectrum of **10.**

Figure S41. NOESY spectrum of **10**.

Figure S42. HSQC spectrum of **10.**

Figure S43. HMBC spectrum of **10.**

Figure S44. ^{1}H NMR spectrum (MeOH-d_4, 400 MHz) of **11.**

Figure S45. ^{13}C NMR spectrum (MeOH-d_4, 100 MHz) of **11.**

Figure S46. COSY spectrum of **11.**

Figure S47. NOESY spectrum of **11**.

Figure S48. HSQC spectrum of **11.**

Figure S49. HMBC spectrum of **11.**

Figure S50. ^{1}H NMR spectrum (MeOH-d_4, 400 MHz) of **12.**

Figure S51. ^{13}C NMR spectrum (MeOH-d_4, 100 MHz) of **12.**

Figure S52. COSY spectrum of **12.**

Figure S53. NOESY spectrum of **12**.

Figure S54. HSQC spectrum of **12.**

Figure S55. HMBC spectrum of **12.**

Figure S56. Dose response curve of compounds **1-12** against *Klebsiella aerogenes* (ATCC® 13048™) and *Pseudomonas aeruginosa* (ATCC® 9027™).

Figure S57. Dose response curve of compounds **1-12** against *Aspergillus fumigatus* (ATCC® 46645™).

Figure S58. Dose response curve of compounds **1-12** against A549 Human lung carcinoma cells (ATCC® CCL-185™).

Table S1. ^{1}H and ^{13}C NMR data of **1** (Decatromicin A) and **2** (Decatromicin B).

Position	**1**		**2**	
	^{13}C	^{1}H, mult. (*J* = Hz)	^{13}C	^{1}H, mult. (*J* = Hz)
1	169.3	–	169.7	–
2	104.1	–	104.0	–
3	205.7	–	205.4	–
4	56.0	–	56.0	–
5	41.7	1.84, m	41.8	1.84, m
6	24.0	1.38, m; 1.79, m	24.0	1.38, m; 1.80, m
7	33.2	1.62, m; 1.62, m	33.4	1.63, m; 1.63, m
8	35.5	2.39, m	35.5	2.39, m
9	87.4	3.38, m	87.4	3.39, m
10	39.7	2.16, t (10.0)	39.8	2.16, t (10.9)
11	125.5	5.68, t (10.4)	125.4	5.66, br d (10.8)
12	132.7	5.70, ddd (2.5, 5.4, 10.0)	132.8	5.70, m
13	43.9	2.89, m	44.0	2.89, m
14	38.0	2.02, m; 2.02, m	38.0	2.01, m; 2.01, m
15	132.6	5.21, m	132.6	5.22, m
16	129.2	5.43, ddd (5.2, 9.5, 15.1)	129.1	5.43, m
17	45.7	2.36, dd (5.0, 12.1); 2.51, m	45.6	2.37, m; 2.50, m
18	140.2	–	140.2	–
19	126.7	5.00, br s	126.9	5.00, br s
20	44.1	–	43.9	–
21	143.9	7.06, d (1.7)	144.1	7.06, s
22	132.8	–	132.8	–
23	37.2	2.71, m	37.2	2.70, m
24	31.0	1.83, m; 2.48, m	31.0	1.81, m; 2.47, m
25	87.0	–	86.9	–
26	200.5	–	200.5	–
27	24.1	1.87, m; 2.74, m	24.1	1.82, m; 2.73, m
28	12.2	0.91, t (7.4)	12.3	0.92, t (7.2)
29	13.5	1.04, d (7.2)	13.5	1.04, d (7.0)
30	26.9	1.31, s	26.9	1.31, s
31	170.5	–	170.5	–
32	27.3	1.59, m; 1.74, m	27.4	1.60, m; 1.73, m
33	13.2	0.92, t (7.4)	13.2	0.92, t (7.2)
1’	103.0	4.52, dd (1.5, 9.6)	102.9	4.53, d (9.1)

2’	41.2	1.58, m; 2.27, ddd (1.2, 4.7, 12.4)	41.1	1.58, m; 2.27, dd (4.3, 12.4)
3’	70.2	3.69, ddd (4.7, 9.7, 11.3)	70.3	3.73, m
4’	59.4	3.55, t (9.9)	59.8	3.59, t (9.5)
5’	72.5	3.41, m	72.5	3.46, m
6’	18.7	1.21, d (6.2)	18.8	1.24, d (6.0)
2”	126.8	–	122.5	–
3”	120.0	–	119.6	–
4”	108.5	6.03, d (3.8)	109.4	6.11, s
5”	112.8	6.77, d (3.8)	114.7	–
6”	163.2	–	161.7	–
18-CH_3	19.2	1.79, s	19.1	1.79, s

^{1}H (400 MHz) and ^{13}C (100 MHz) in MeOH-*d*$_4$. Assignments based on COSY, NOESY, HSQC and HMBC and comparison with literature compounds. Chemical shifts (δ) in ppm. s: singlet; br s: broad singlet; d: doublet; br d: broad doublet; t: triplet, m: multiplet. One proton unless otherwise stated.

Table S2. ^{1}H NMR data of **3** (BE-45722B), **4** (BE-45722C) and **5** (BE-45722D).

Position	**3**	**4**	**5**
	^{1}H, mult. (J = Hz)	^{1}H, mult. (J = Hz)	^{1}H, mult. (J = Hz)
5	1.79-1.91, m	1.80-1.91, m	1.72-1.95, m
6	1.36, m; 1.79-1.91, m	1.38, m; 1.80-1.91, m	1.38, m; 1.72-1.95, m
7	1.61, m; 1.61, m	1.63, m; 1.63, m	1.54-1.68, m
8	2.39, m	2.36, m	2.36, m
9	3.39, dd (5.0, 10.7)	3.39, dd (4.8, 10.6)	3.39, m
10	2.16, br t (10.7)	2.17, br t (11.0)	2.17, br t (10.9)
11	5.64, br d (10.3)	5.66, br d (10.4)	5.66, d (11.1)
12	5.70, ddd (2.1, 5.5, 9.9)	5.71, ddd (2.0, 5.7, 10.1)	5.71, ddd (1.8, 5.2, 10.0)
13	2.91, m	2.89, m	2.89, m
14	2.00, m; 2.00, m	2.01, m; 2.01, m	2.01, m; 2.01, m
15	5.24, dt (6.4, 14.5)	5.19, dt (6.7, 14.6)	5.19, m
16	5.43, ddd (4.6, 8.5, 14.8)	5.43, ddd (5.2, 9.2, 14.8)	5.43, ddd (5.1, 9.4, 15.0)
17	2.40, m; 2.47, m	2.40, m; 2.47, m	2.40, m; 2.46, m
19	5.00, s	4.99, s	4.99, s
21	7.05, d (1.5)	7.06, d (1.5)	7.06, d (1.5)
23	2.73, m	2.71, m	2.64-2.71, m
24	1.79-1.91, m; 2.50, m	1.80-1.91, m; 2.50, m	1.72-1.95, m; 2.50, m
27	1.79-1.91, m; 2.68, m	1.80-1.91, m; 2.69, m	1.72-1.95, m, 2.69, m
28	0.93, t (7.3)	0.92, t (7.3)	0.91, d (7.4)
29	1.02, d (7.0)	1.04, d (7.0)	1.04, d (7.1)
30	1.31, s	1.30, s	1.31, s
32	1.61, m; 1.71, m	1.60, m; 1.75, m	1.54-1.68, m; 1.72-1.95, m
33	0.91, t (7.0)	0.91, t (7.5)	0.92, t (7.3)
1’	4.52, dd (1.2, 8.8)	4.52, dd (1.3, 9.5)	4.52, dd (1.4, 9.6)
2’	1.59, m; 2.26, ddd (1.2, 4.8, 12.4)	1.59, m; 2.29, m	1.54-1.68, m
3’	3.73, ddd (5.2, 9.7, 11.5)	3.70, m	3.70, m
4’	3.60, t (9.6)	3.60, t (9.7)	3.61, t (9.7)
5’	3.47, m	3.46, m	3.45, m

6’	1.24, d (6.1)	1.29, d (7.0)	1.29, d (6.3)
4”	–	–	6.14, s
18-CH_3	1.78, d (1.0)	1.79, s	1.78, s
CH_3-1”	–	3.75, s	3.72, s

^{1}H (400 MHz) in MeOH-d_4. Assignments based on comparison with literature compounds. Chemical shifts (δ) in ppm. s: singlet; br s: broad singlet; d: doublet; br d: broad doublet; t: triplet, m: multiplet. One proton unless otherwise stated.

Table S3. ^{1}H NMR data of **6** (Pyrrolosporin A).

Position	[a]**6**	[b]**6**
	[1]H, mult. (J = Hz)	[1]H, mult. (J = Hz)
5	1.75, m	1.67-1.92, m
6	1.25, m; 1.70, m	1.37, m; 1.67-1.92, m
7	1.52, m; 1.52, m	1.44-1.65, m; 1.44-1.65, m
8	2.29, m	2.38, m
9	3.32, m	3.38, dd (4.8, 10.8)
10	2.06, br t (10.6)	2.16, t (10.3)
11	5.59, m	5.63, m
12	5.69, m	5.70, ddd (2.0, 5.5, 10.1)
13	2.82, m	2.90, m
14	1.88, m; 1.92, m	1.98, m; 1.98, m
15	5.14, m	5.20, m
16	5.26, m	5.33, m
17	2.47, m; 2.47, m	2.46-2.59, m; 2.46-2.59, m
18	5.50, dd (8.0, 15.8)	5.66, m
19	5.32, d (15.9)	5.39, d (15.8)
21	6.63, d (1.0)	6.77, d (1.8)
23	2.59, m	2.70, m
24	1.71, m; 2.45, m	1.67-1.92, m; 2.46-2.59, m
27	1.75, m; 2.64, m	1.67-1.92, m; 2.74, m
28	0.84, t (7.0)	0.93, t (7.3)
29	0.95, d (7.0)	1.03, d (7.1)
30	1.21, s	1.29, s
32	1.42, m; 1.62, m	1.44-1.65, m; 1.67-1.92, m
33	0.83, t (6.9)	0.92, t (7.5)
1’	4.43, d (8.5)	4.52, d (9.0)
2’	1.40, m; 2.12, m	1.44-1.65, m; 2.26, m
3’	3.61, m	3.72, ddd (4.8, 9.7, 14.5)
4’	3.43, m	3.59, t (9.7)
5’	3.36, m	3.46, m
6’	1.09, d (6.0)	1.24, d (6.4)
4”	6.24, d (2.5)	6.11, s
4’-NH	7.08, br s	–
1”-NH	12.50, br s	–

[a]^{1}H (400 MHz) and ^{13}C (100 MHz) in DMSO-d_6. [b]^{1}H (400 MHz) and ^{13}C (100 MHz) in MeOH-d_4. Assignments based on comparison with literature compounds. [c]Not determined. Chemical shifts (δ) in ppm. s: singlet; br s: broad singlet; d: doublet; br d: broad doublet; t: triplet, m: multiplet. One proton unless otherwise stated.

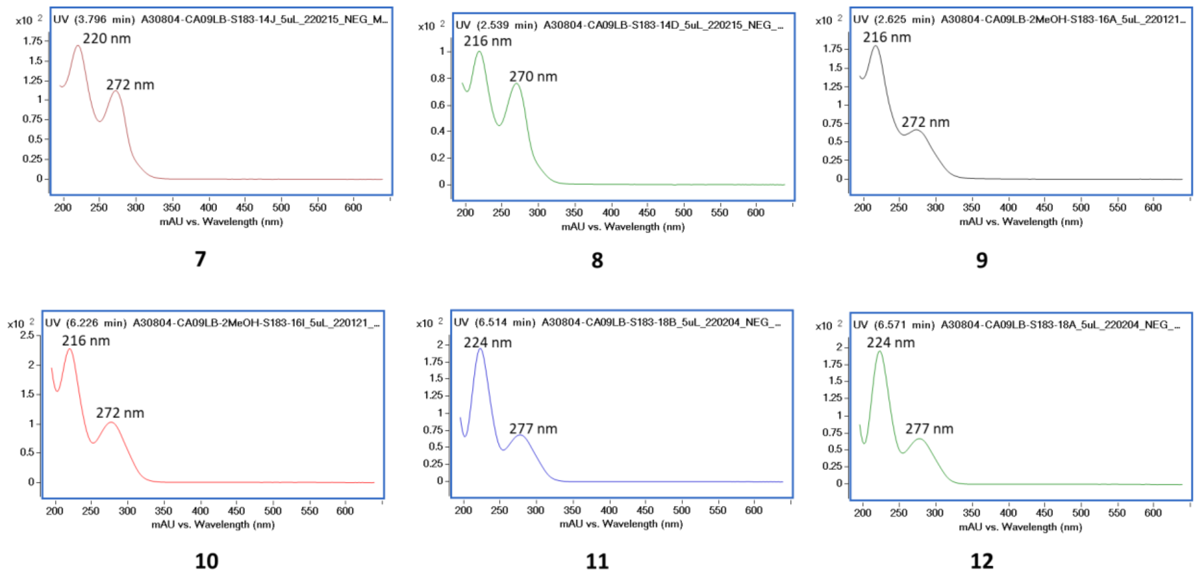

Figure S1. UV spectra for compounds **7**–**12**.

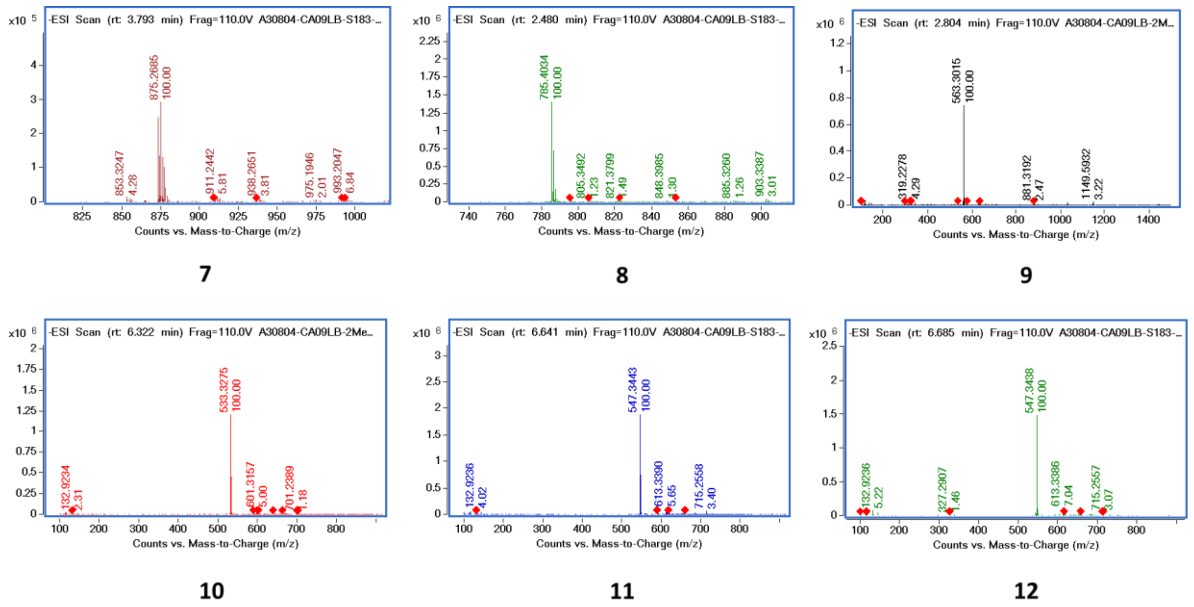

Figure S2. (–)-HRESIMS spectra for compounds **7**–**12**.

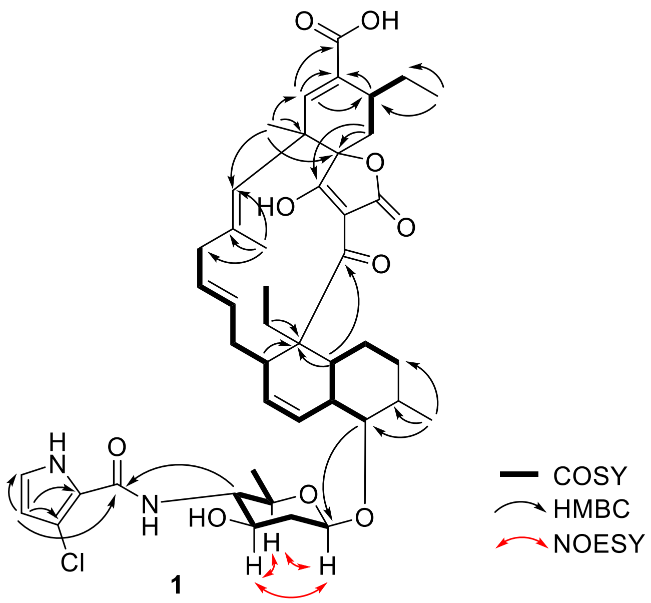

Figure S3. Selected COSY, HMBC and NOESY correlations of **1**.

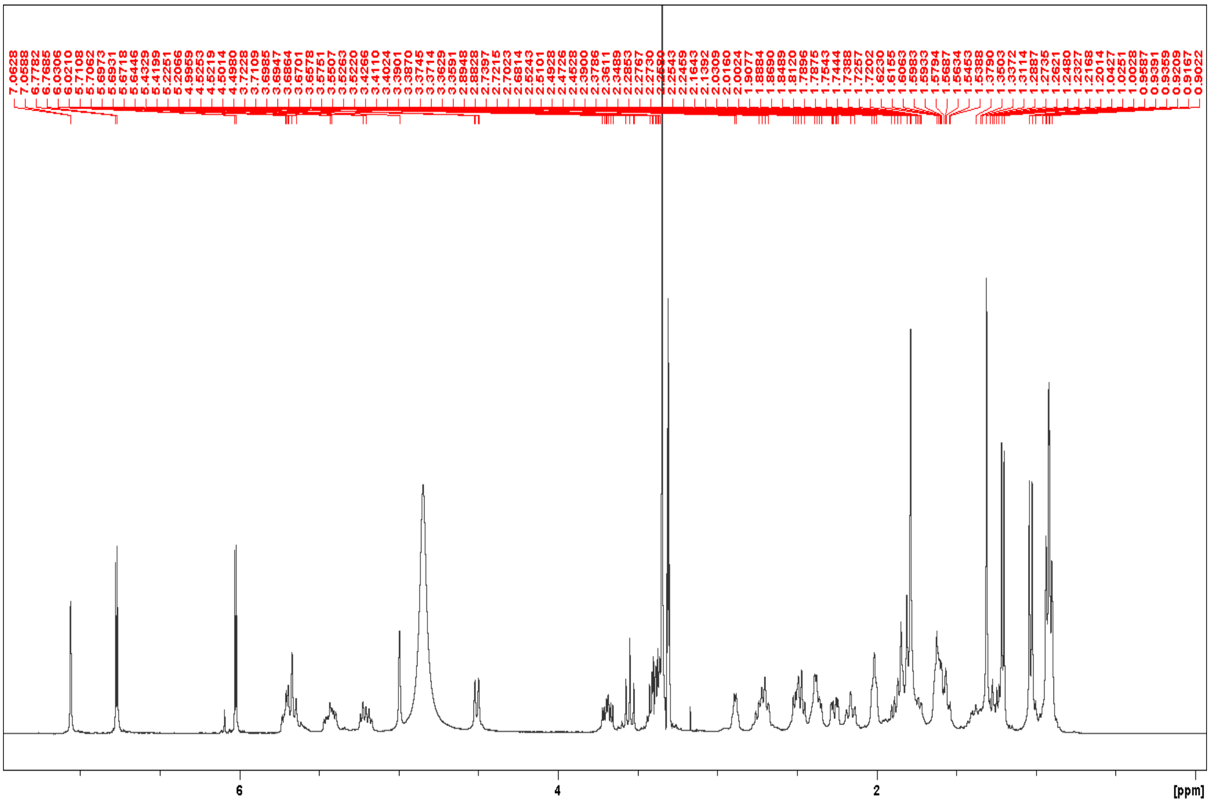

Figure S4. ^{1}H NMR spectrum (MeOH-d_4, 400 MHz) of **1.**

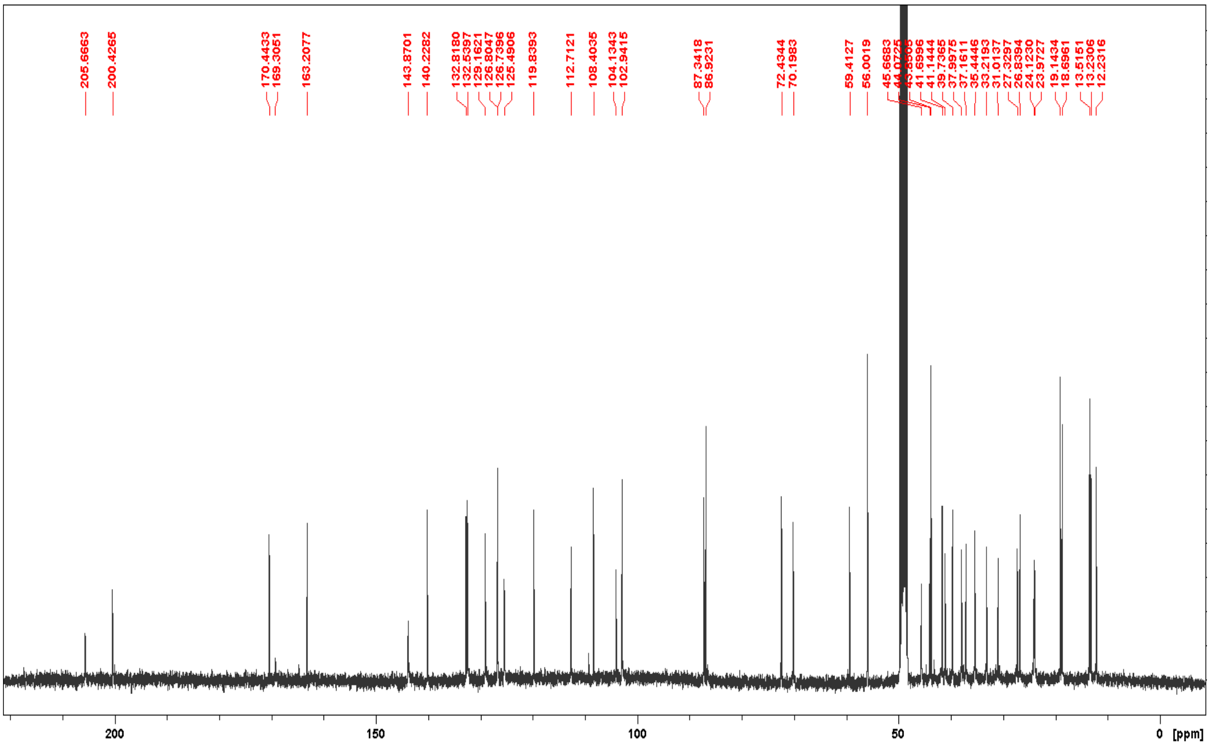

Figure S5. ^{13}C NMR spectrum (MeOH-d_4, 100 MHz) of **1**.

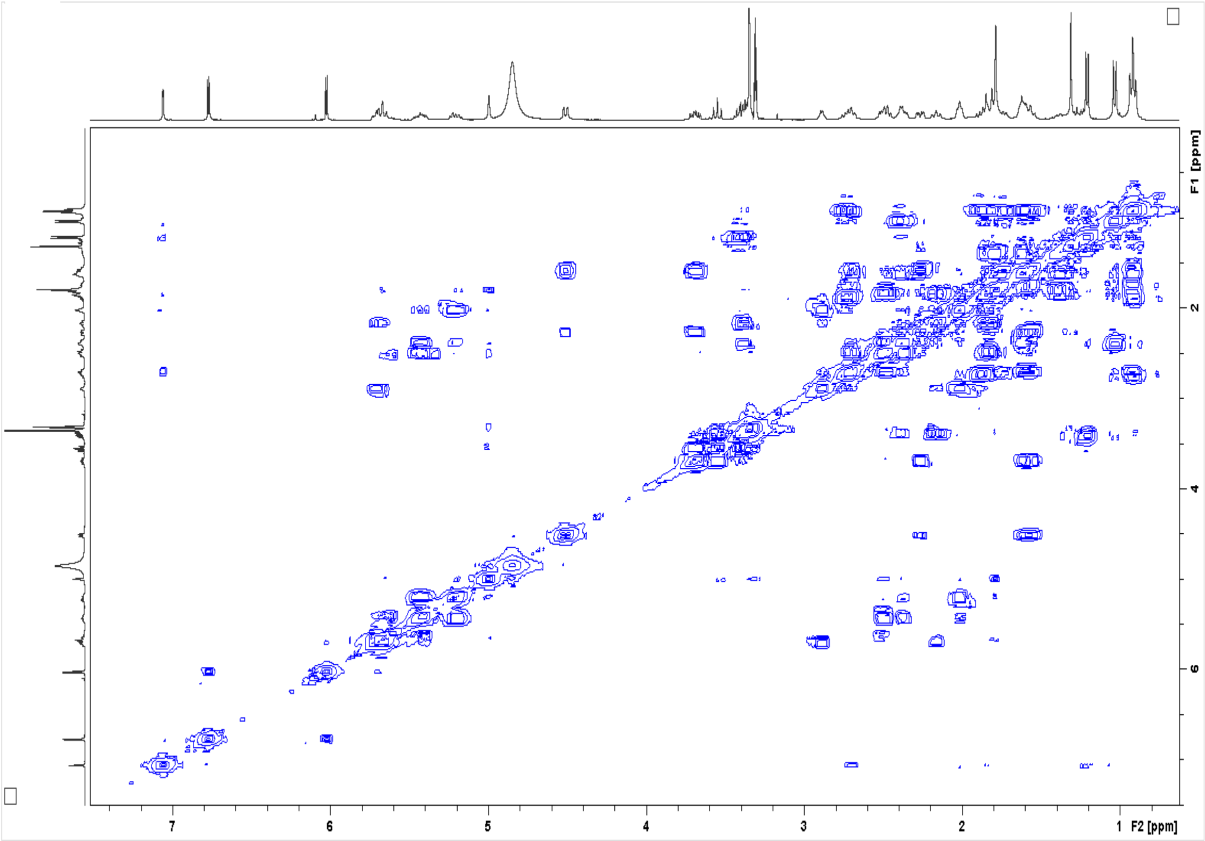

Figure S6. COSY spectrum of **1**.

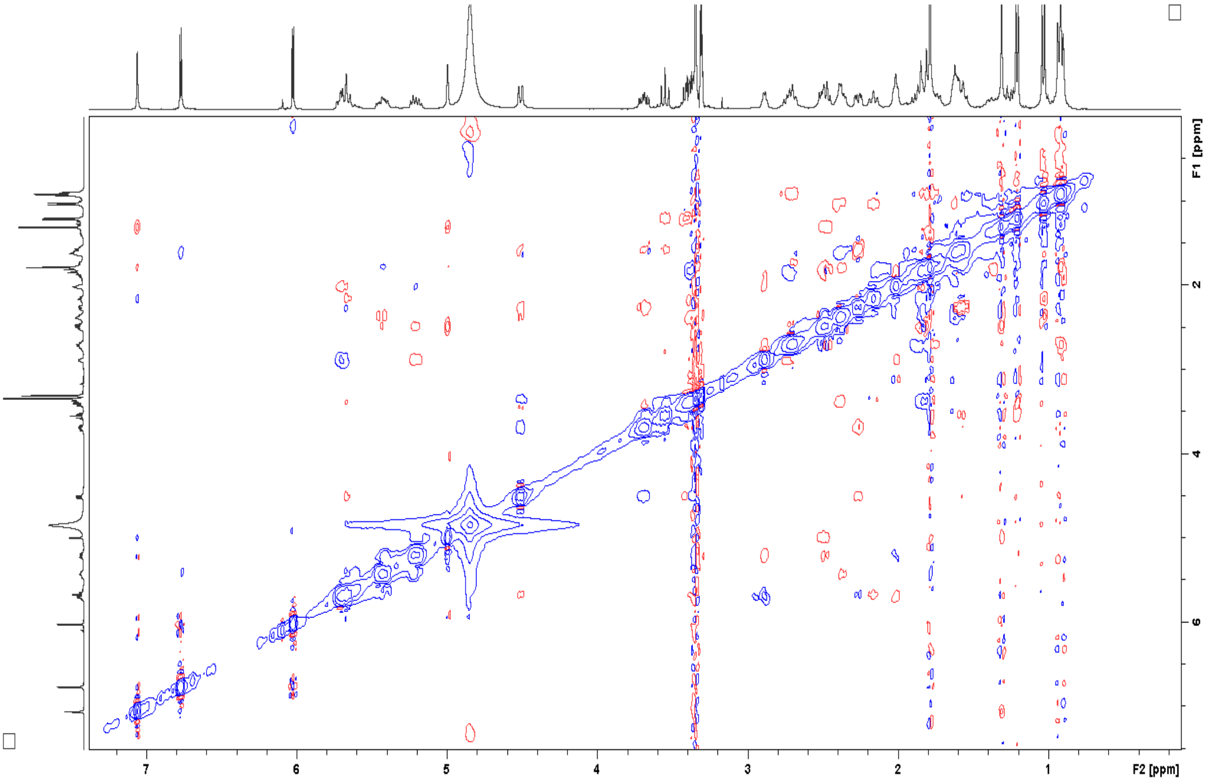

Figure S7. NOESY spectrum of **1**.

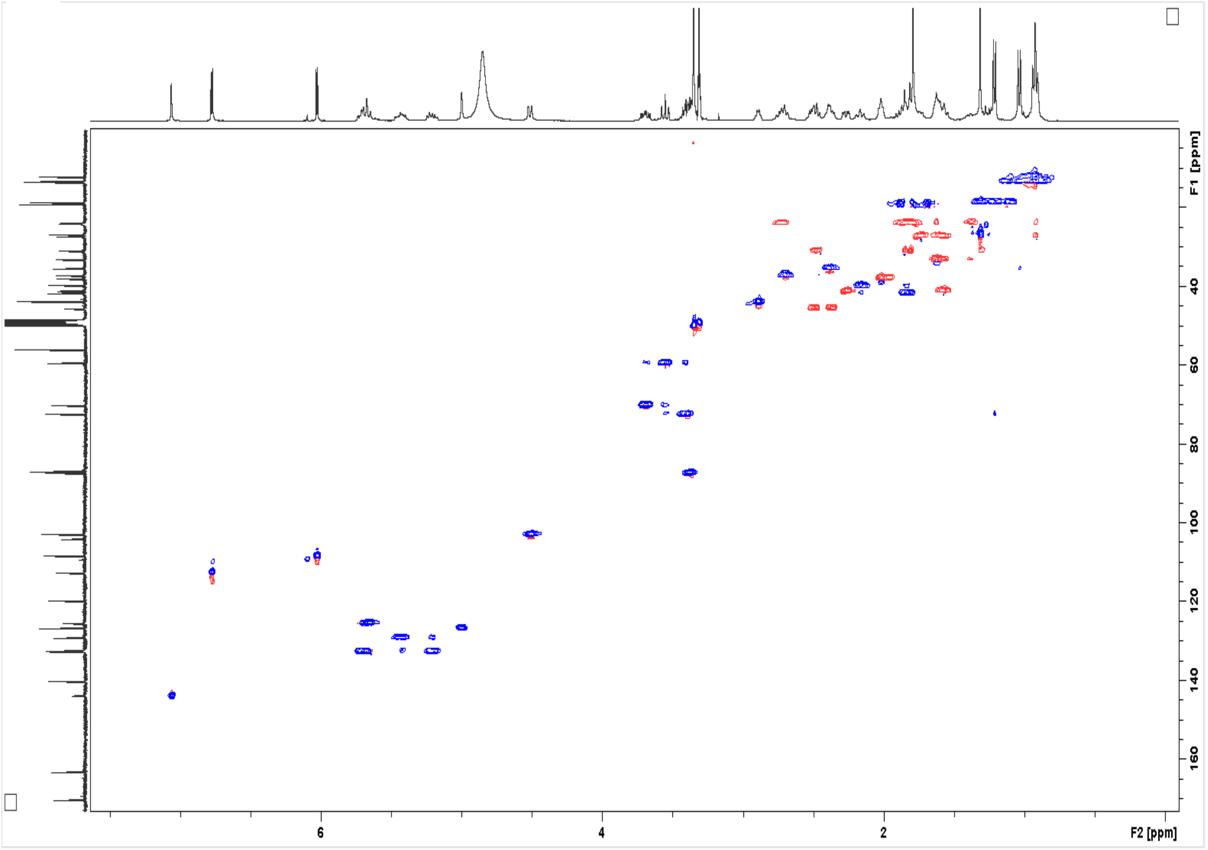

Figure S8. HSQC spectrum of **1**.

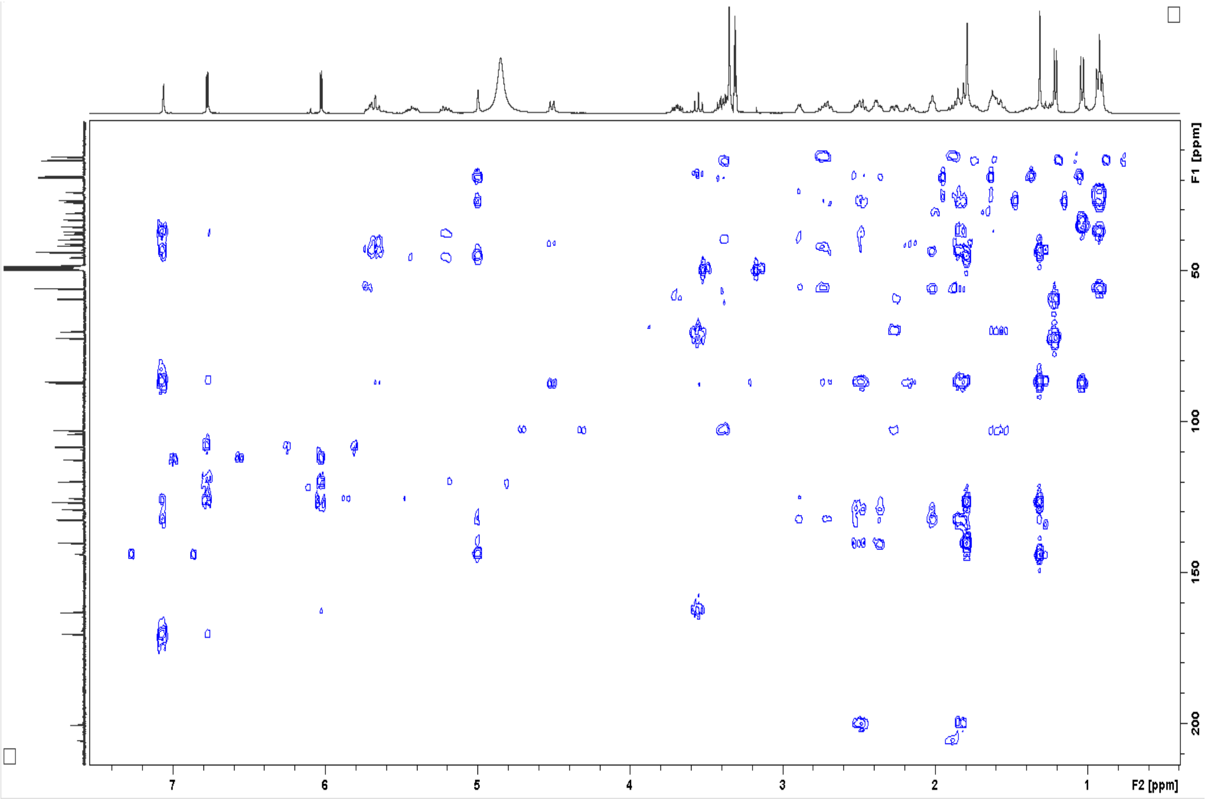

Figure S9. HMBC spectrum of **1**.

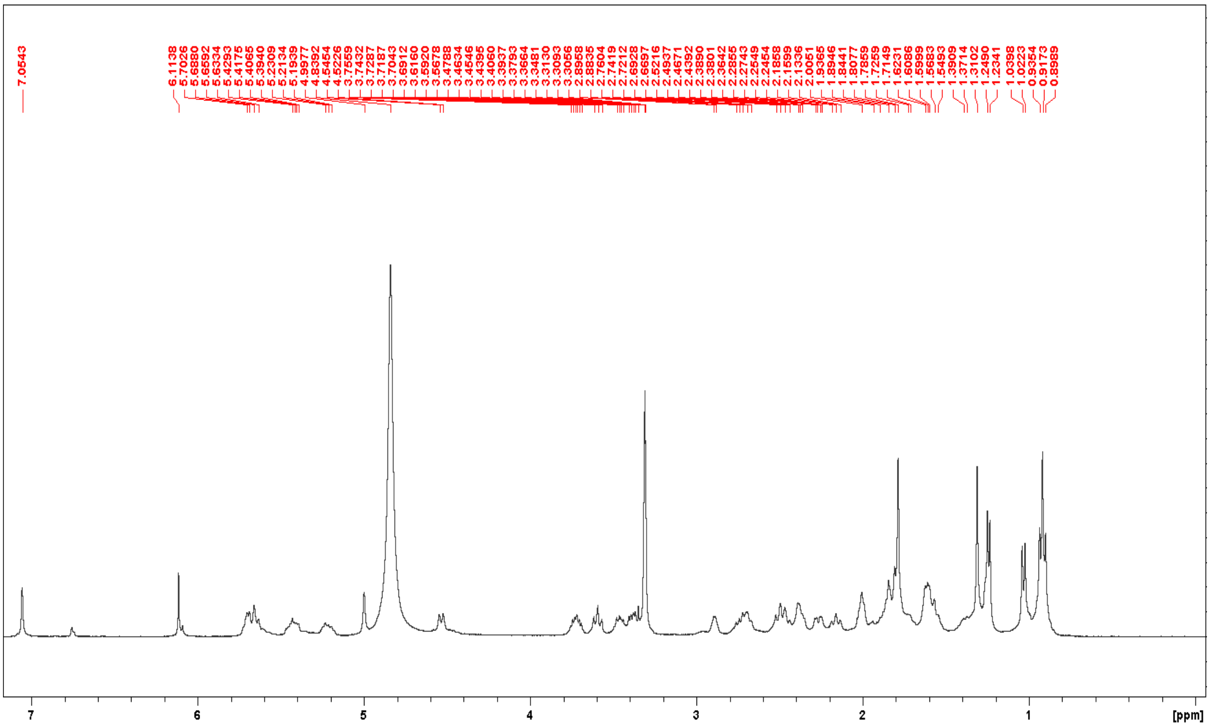

Figure S10. ^{1}H NMR spectrum (MeOH-*d*$_4$, 400 MHz) of **2.**

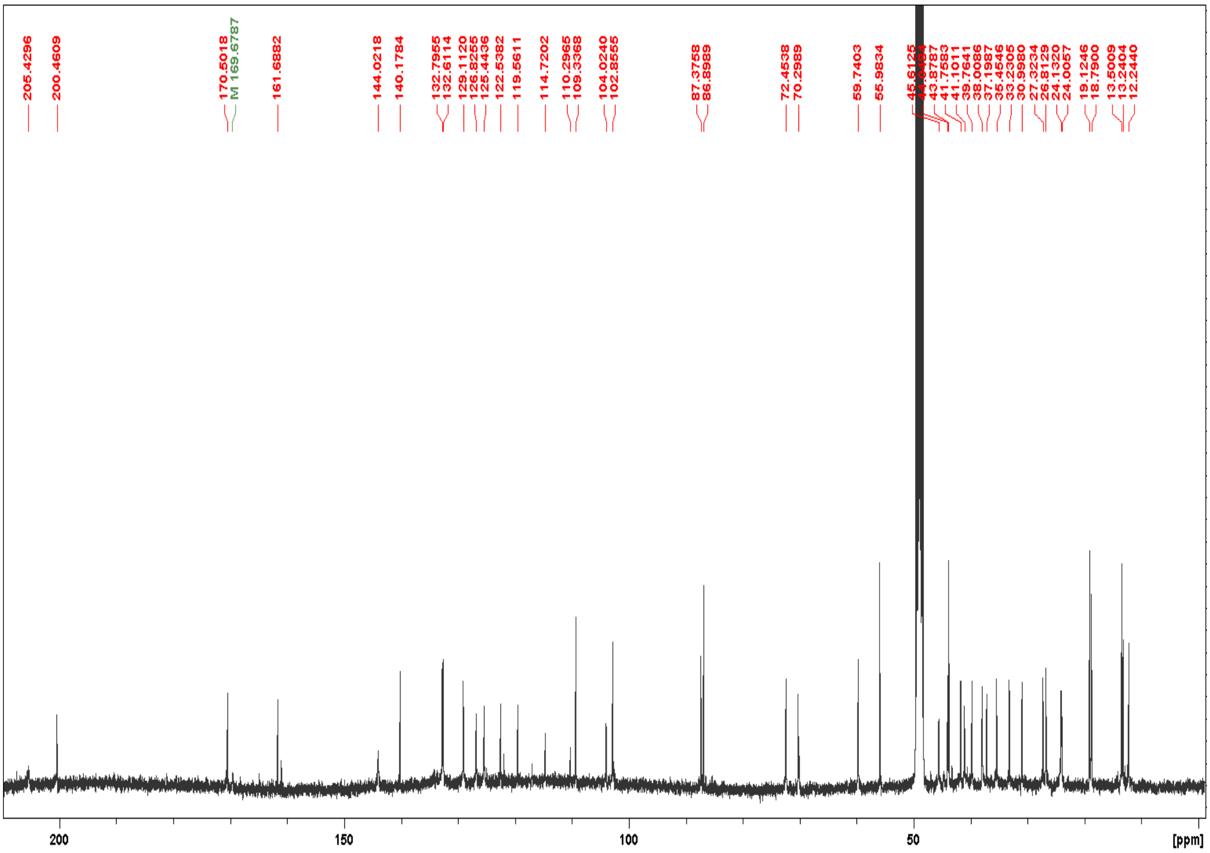

Figure S11. ^{13}C NMR spectrum (MeOH-d_4, 100 MHz) of **2**.

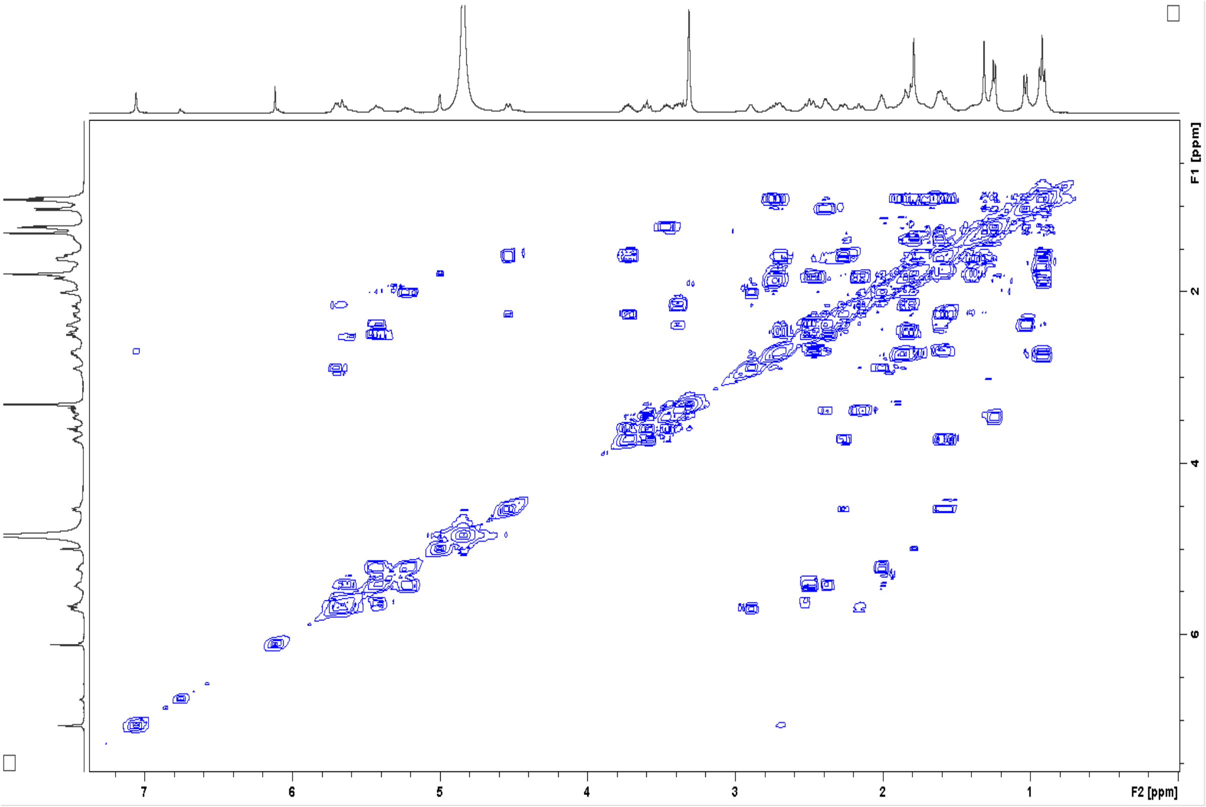

Figure S12. COSY spectrum of **2**.

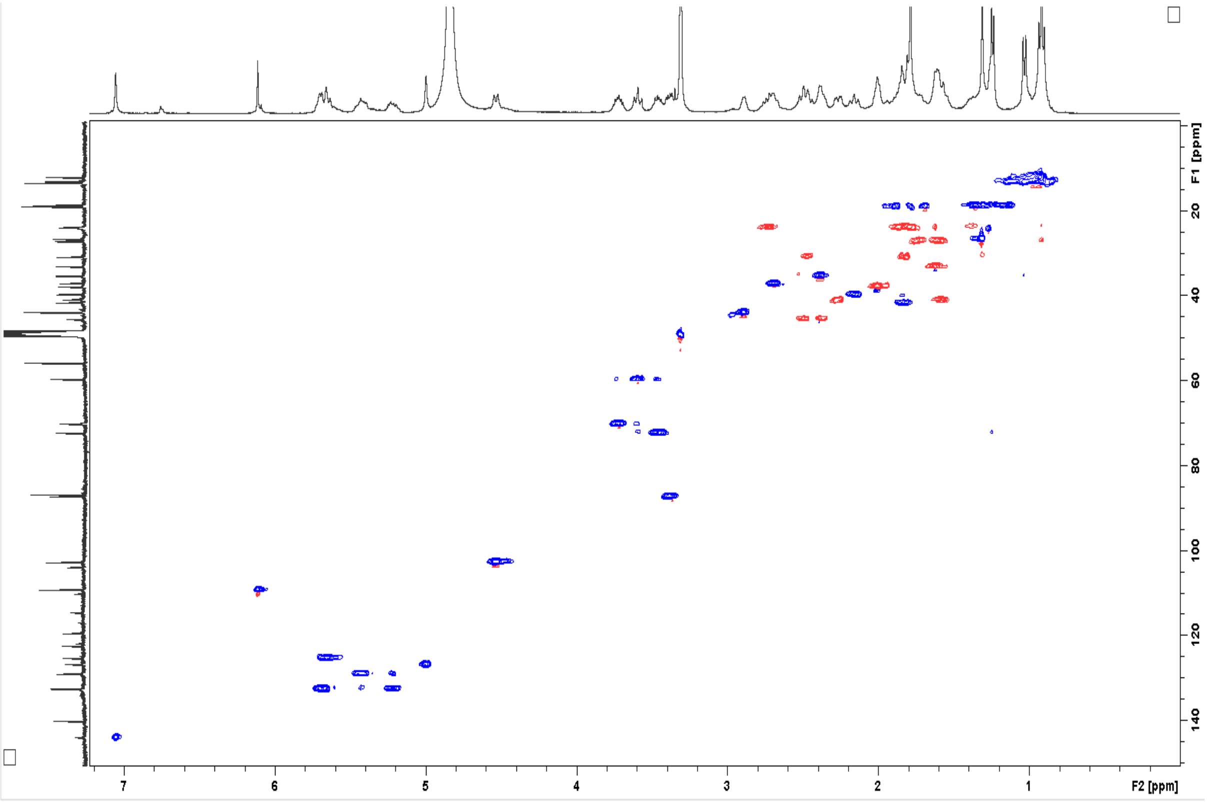

Figure S13. HSQC spectrum of **2**.

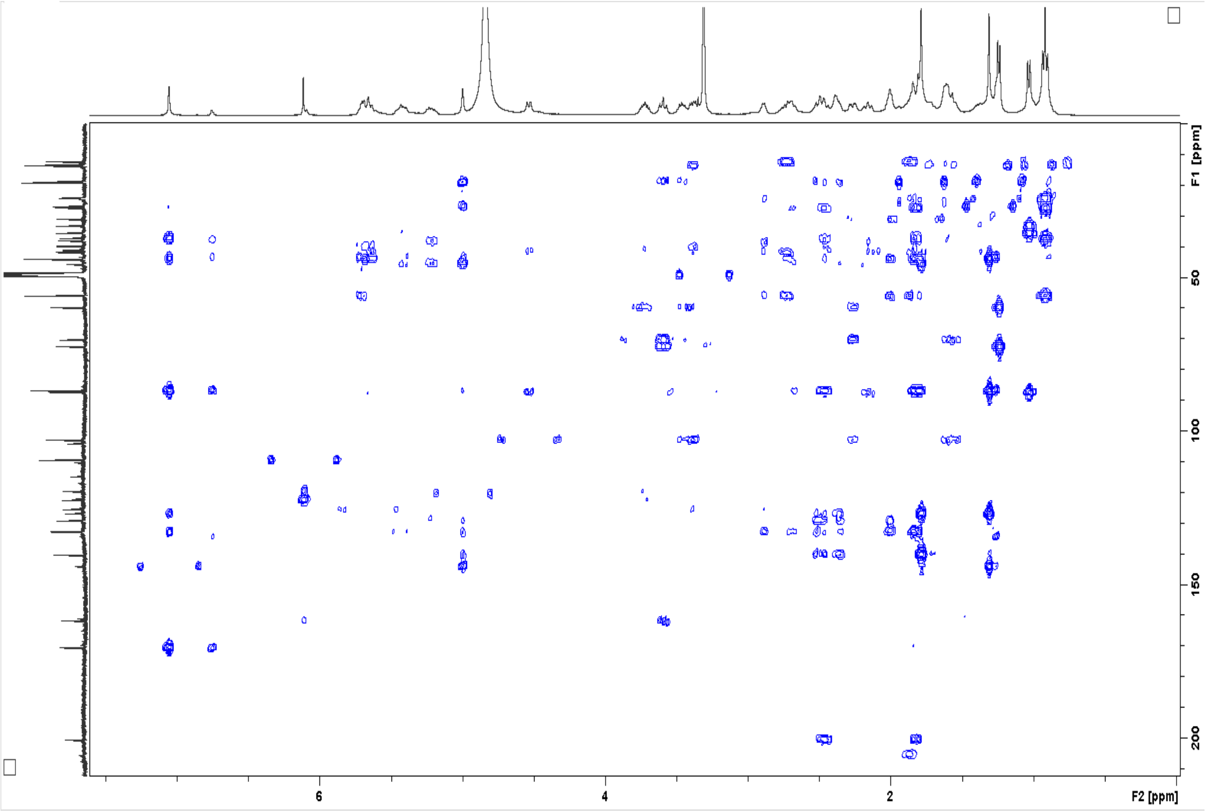

Figure S14. HMBC spectrum of **2**.

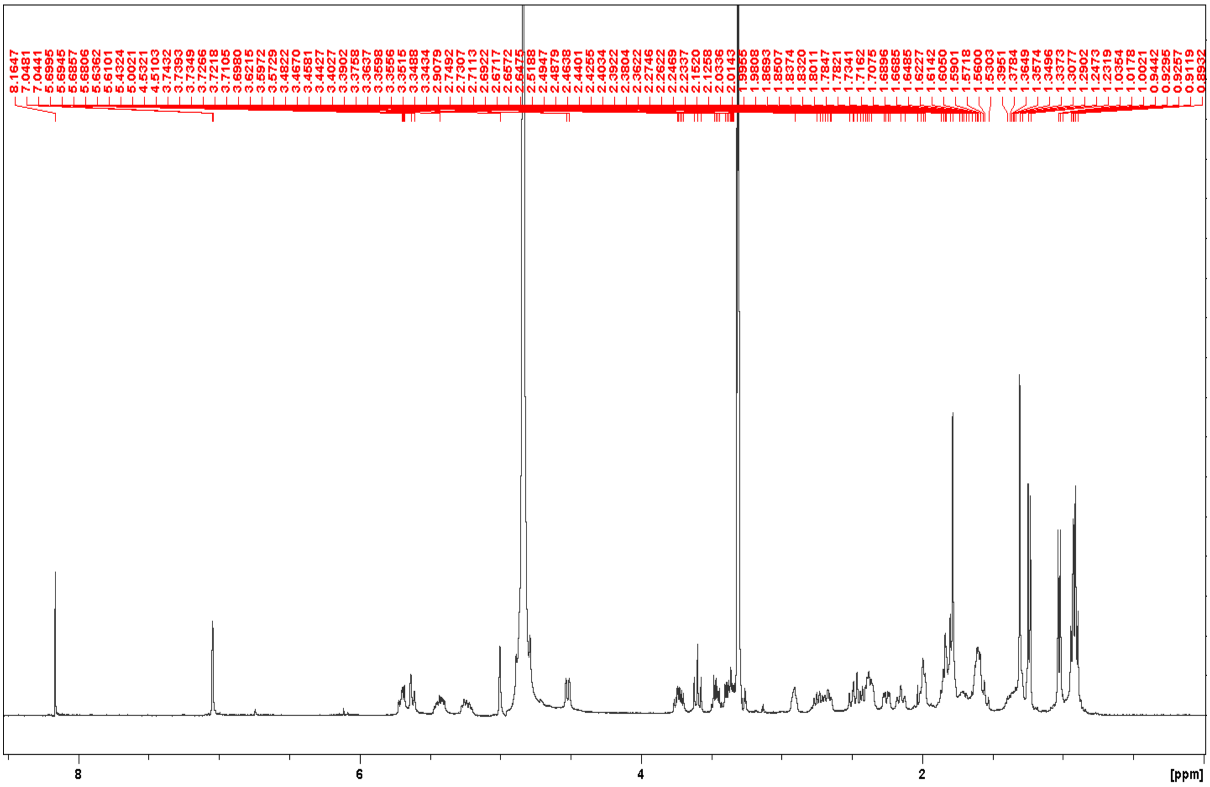

Figure S15. ^{1}H NMR spectrum (MeOH-d_4, 400 MHz) of **3**.

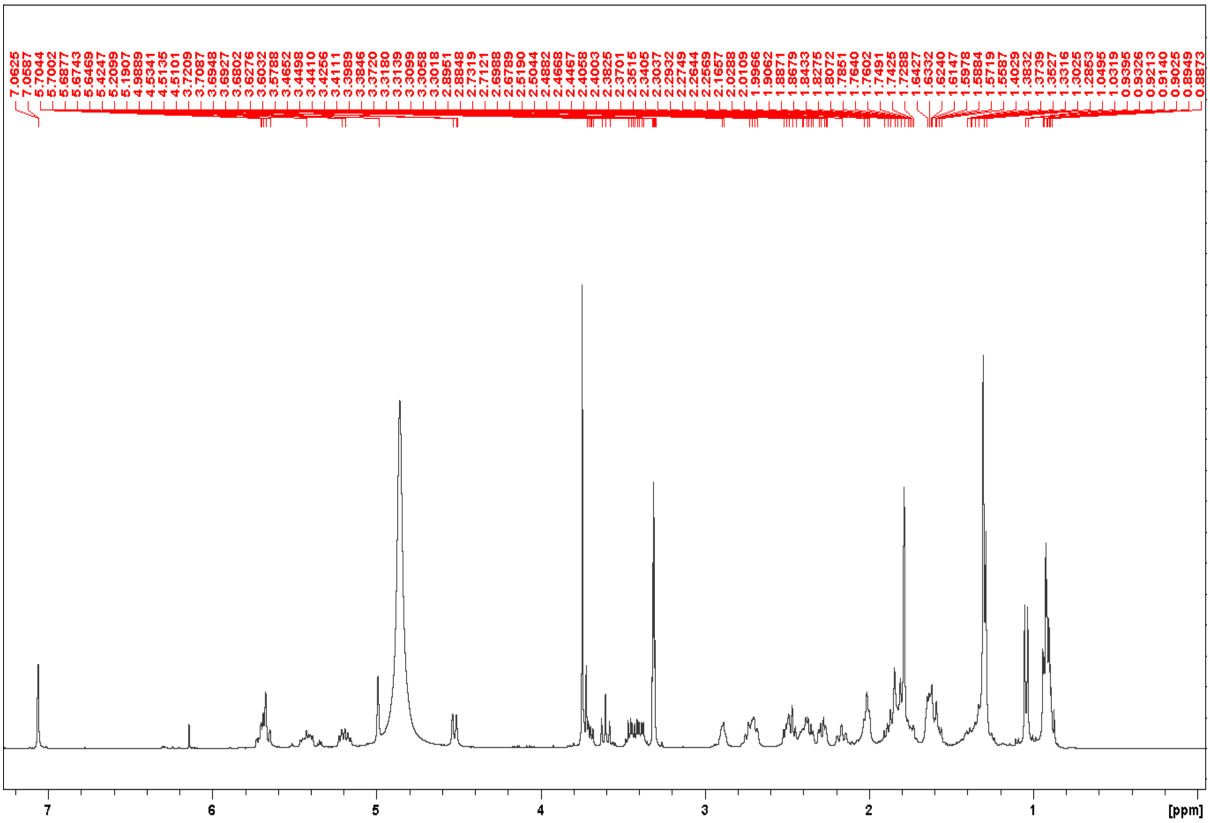

Figure S16. ^{1}H NMR spectrum (MeOH-d_4, 400 MHz) of **4**.

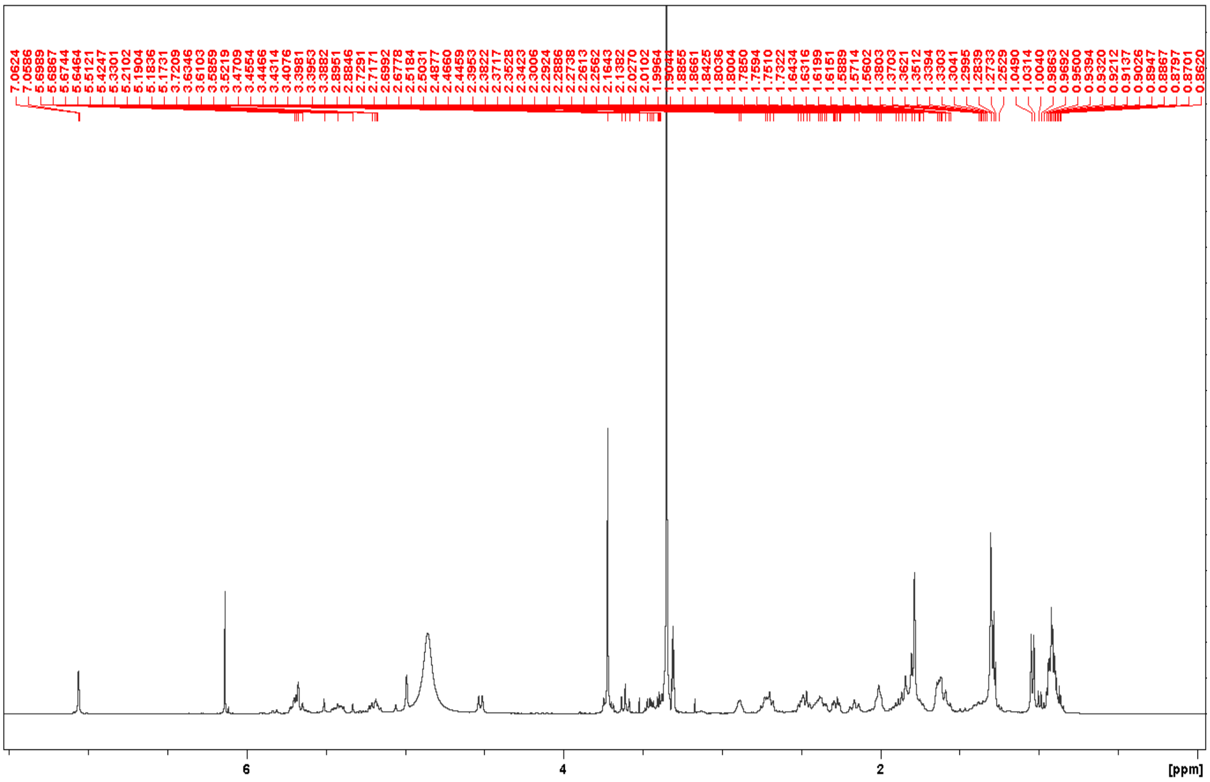

Figure S17. ^{1}H NMR spectrum (MeOH-d_4, 400 MHz) of **5**.

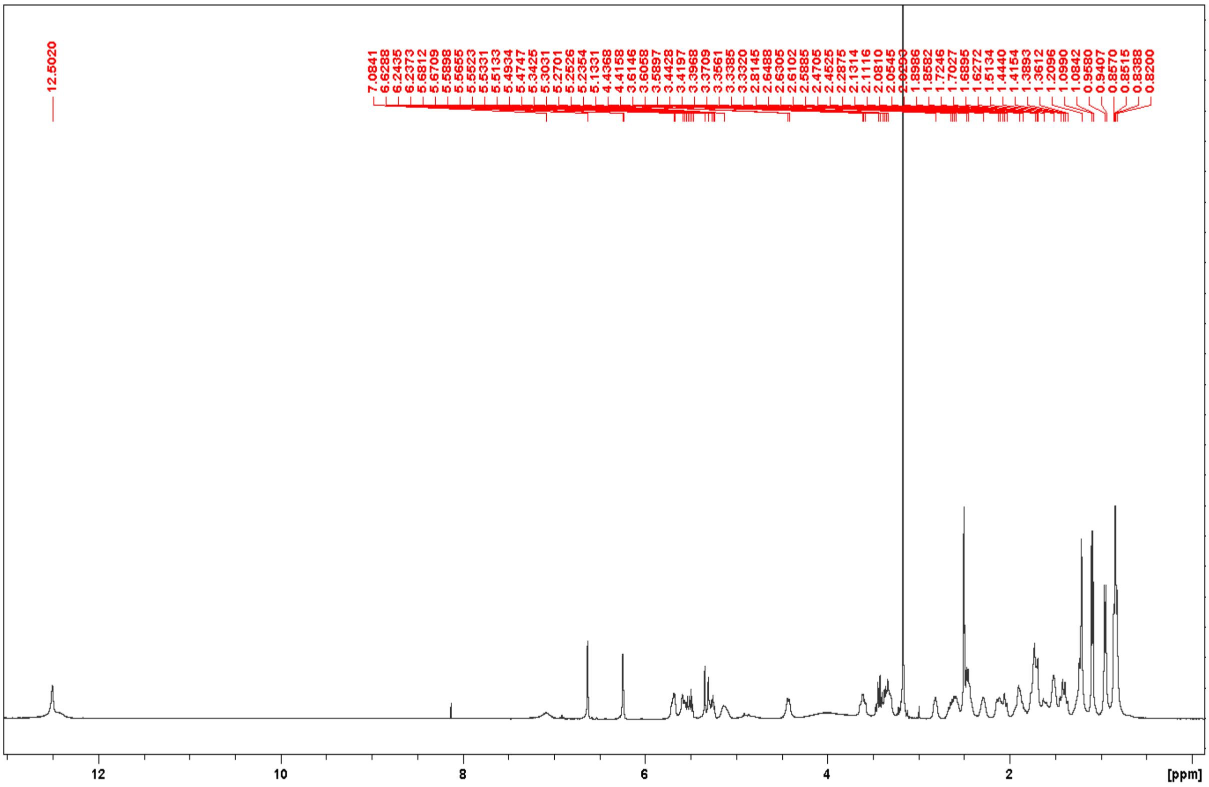

Figure S18. ^{1}H NMR spectrum (DMSO-d_6, 400 MHz) of **6**.

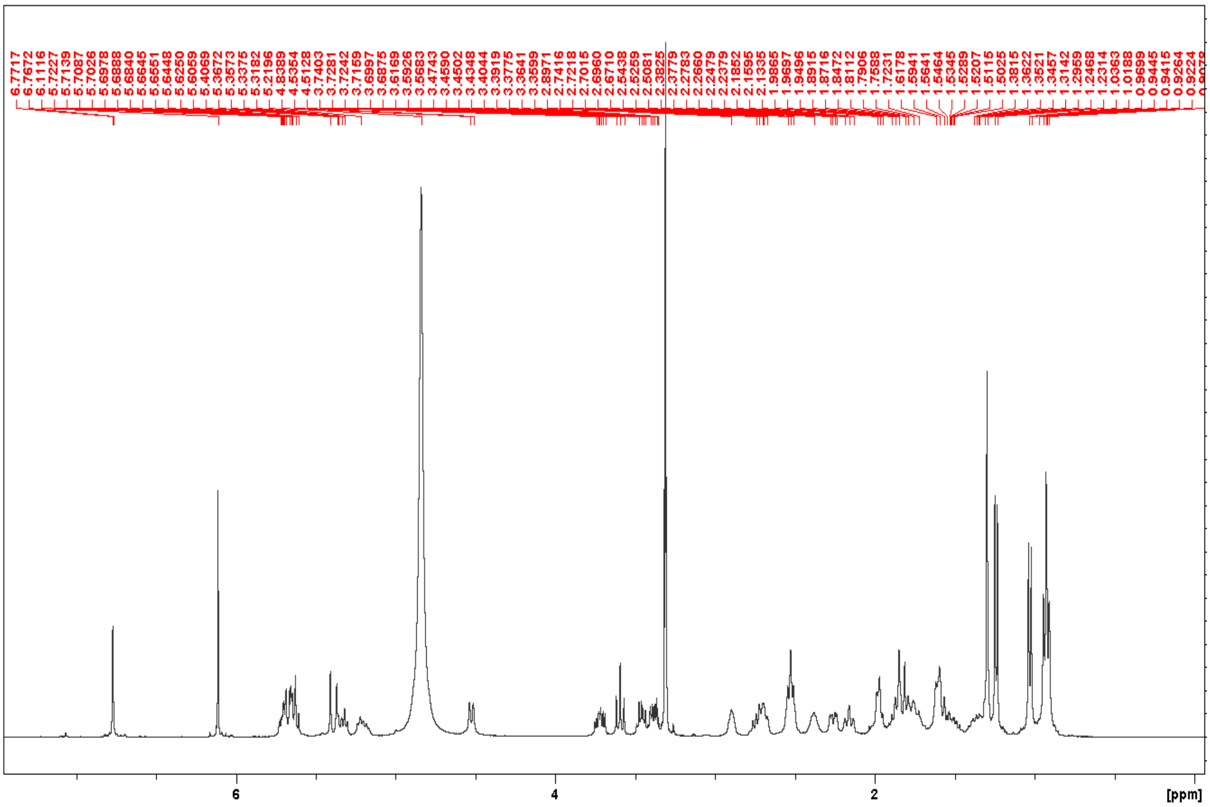

Figure S19. ¹H NMR spectrum (MeOH-*d₄*, 400 MHz) of **6**.

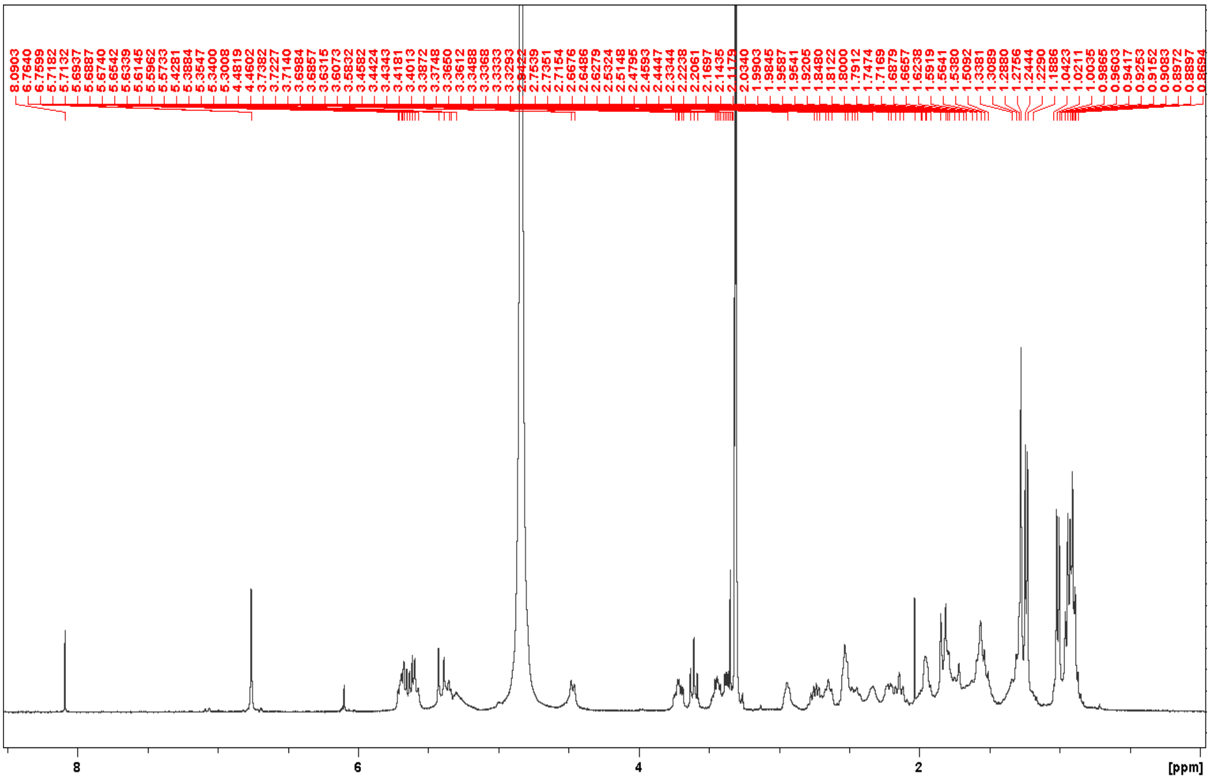

Figure S20. ¹H NMR spectrum (MeOH-*d₄*, 400 MHz) of **7**.

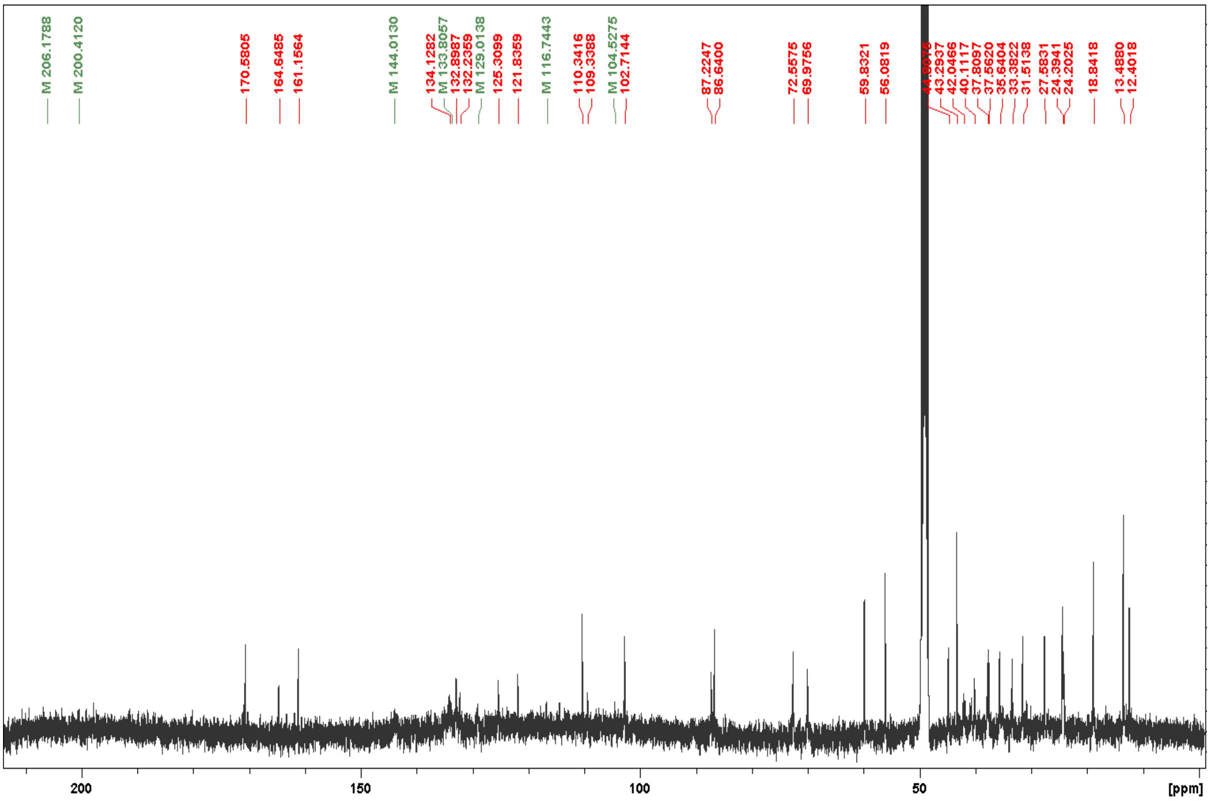

Figure S21. ^{13}C NMR spectrum (MeOH-d_4, 100 MHz) of **7**.

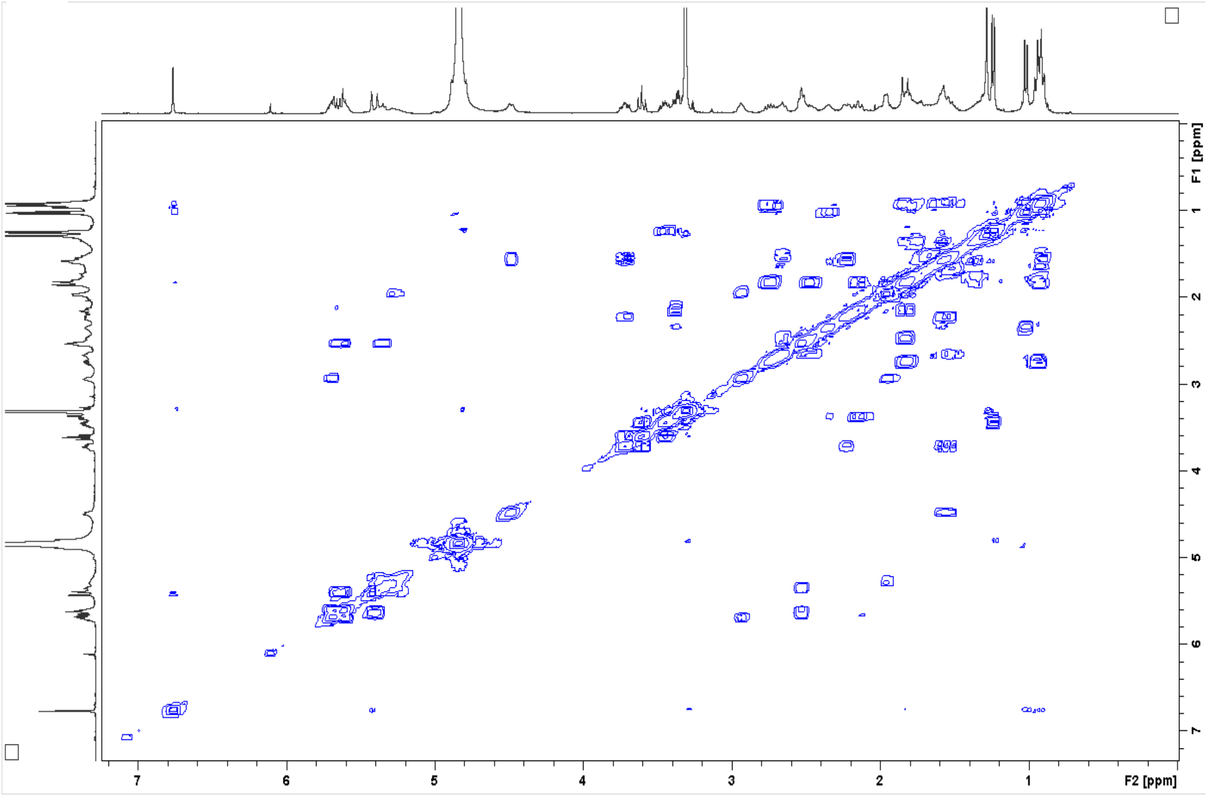

Figure S22. COSY spectrum of **7**.

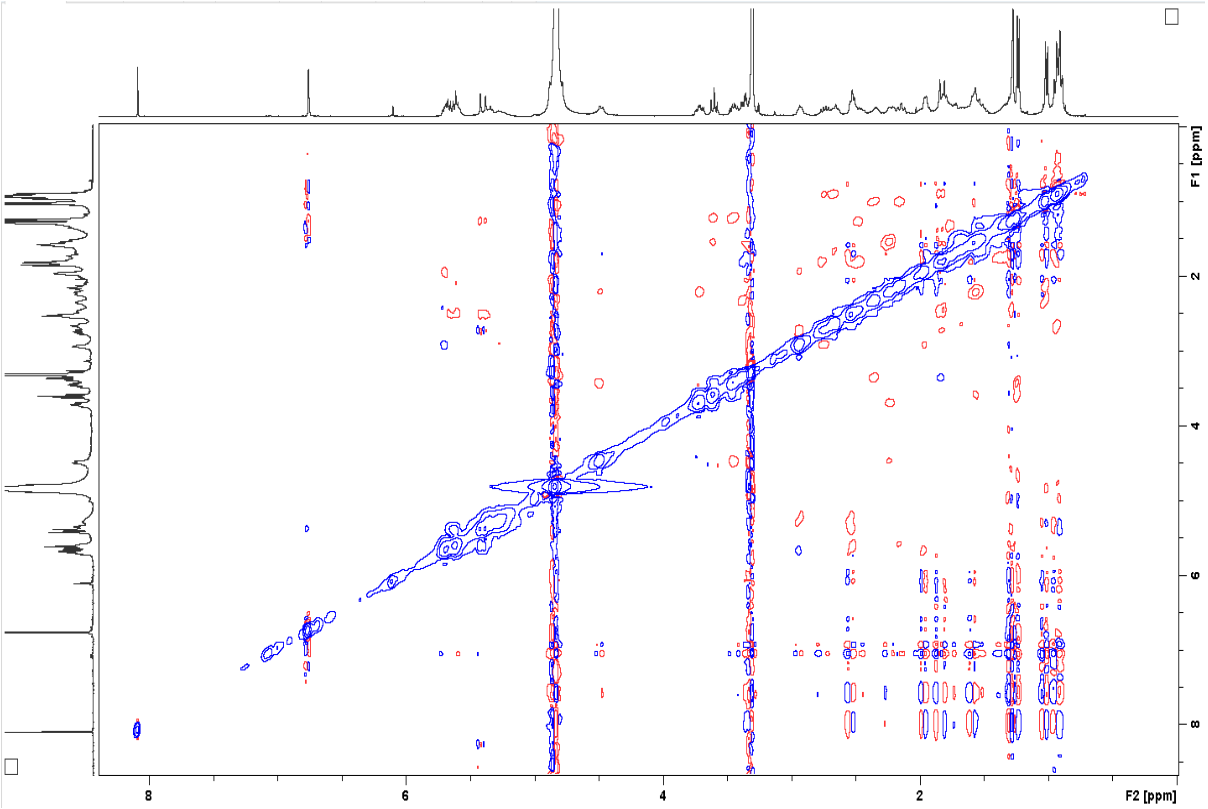

Figure S23. NOESY spectrum of **7**.

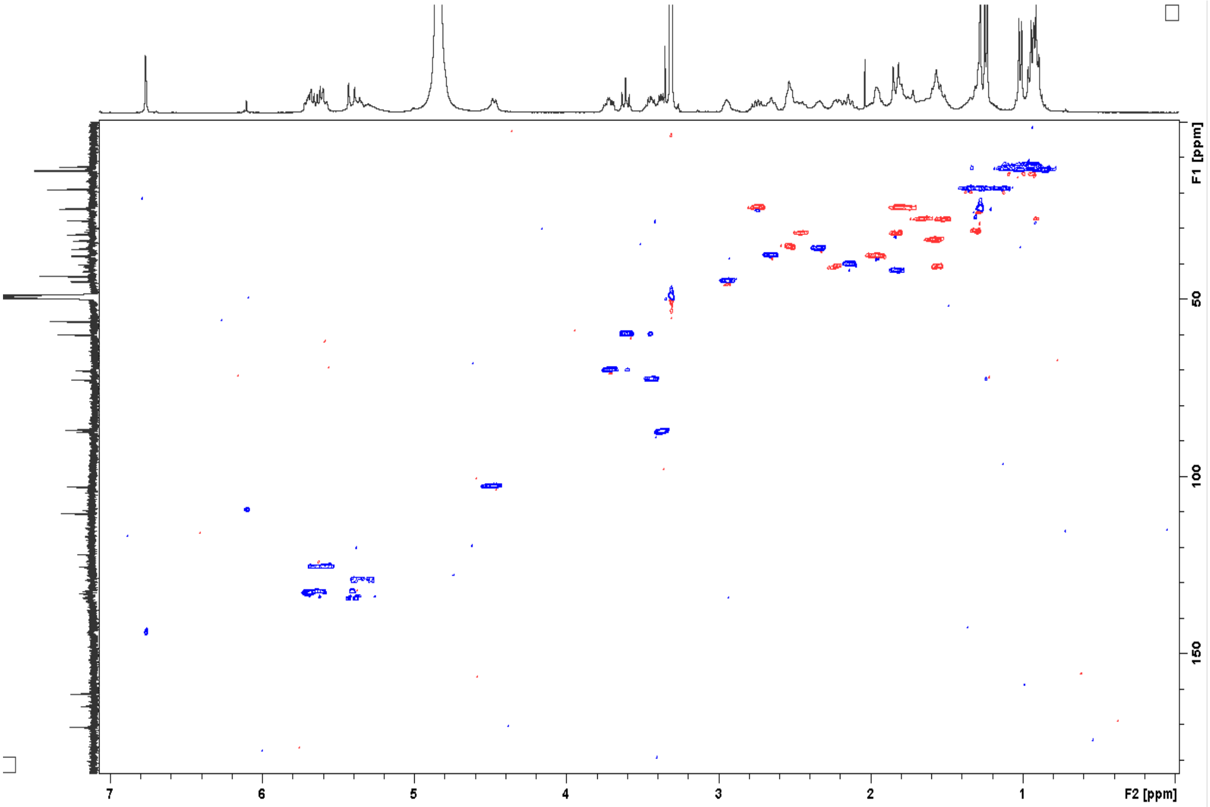

Figure S24. HSQC spectrum of **7**.

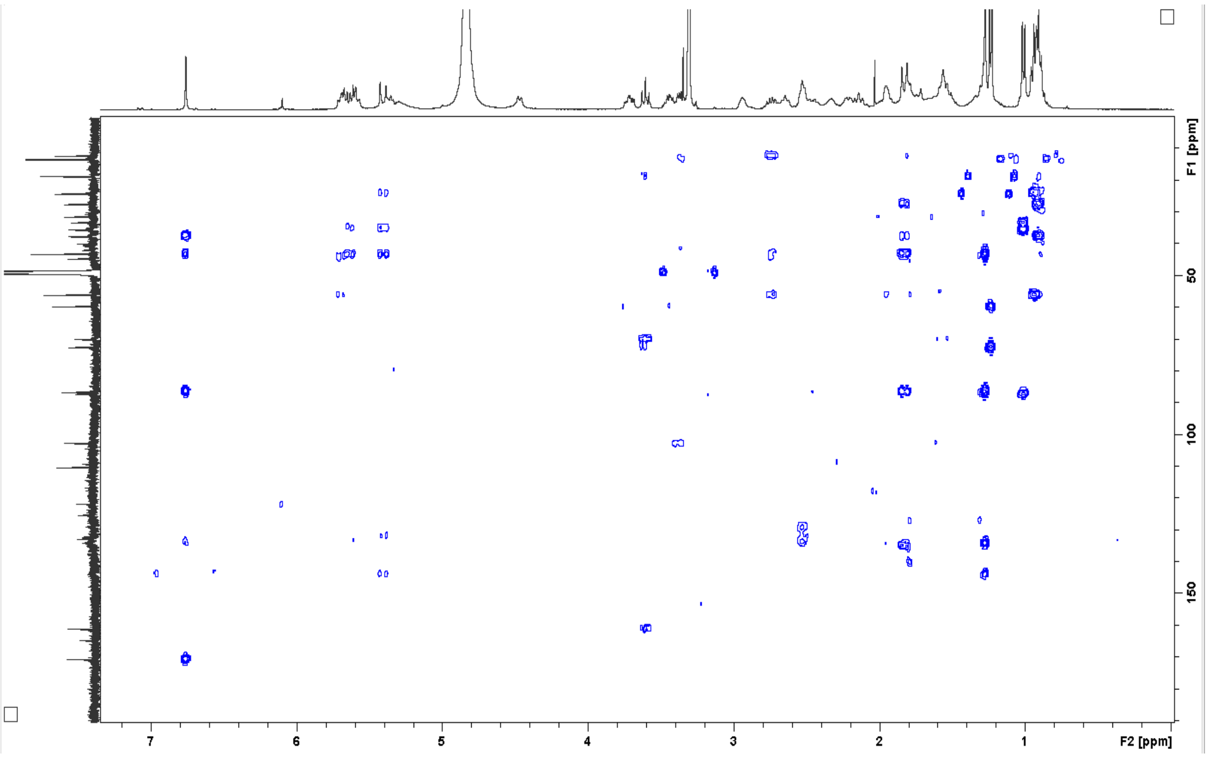

Figure S25. HMBC spectrum of **7**.

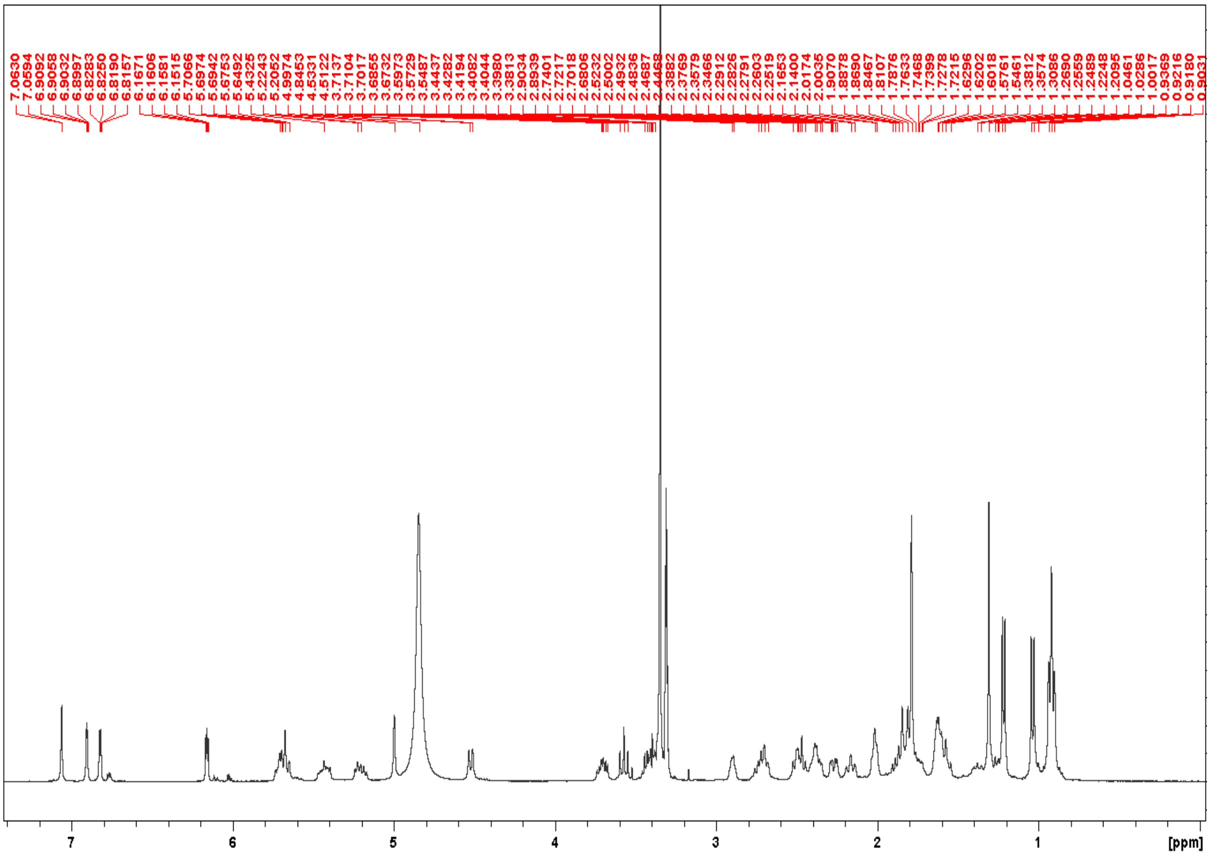

Figure S26. ^{1}H NMR spectrum (MeOH-d_4, 400 MHz) of **8.**

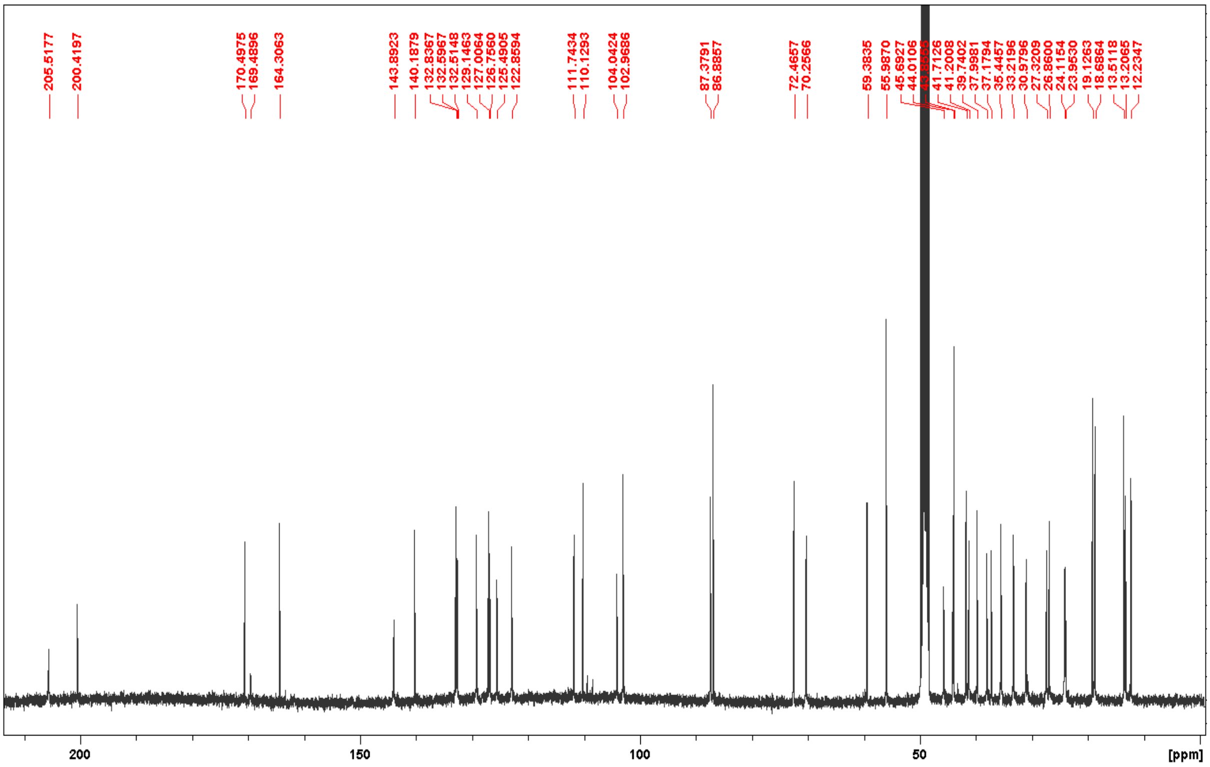

Figure S27. ^{13}C NMR spectrum (MeOH-d_4, 100 MHz) of **8.**

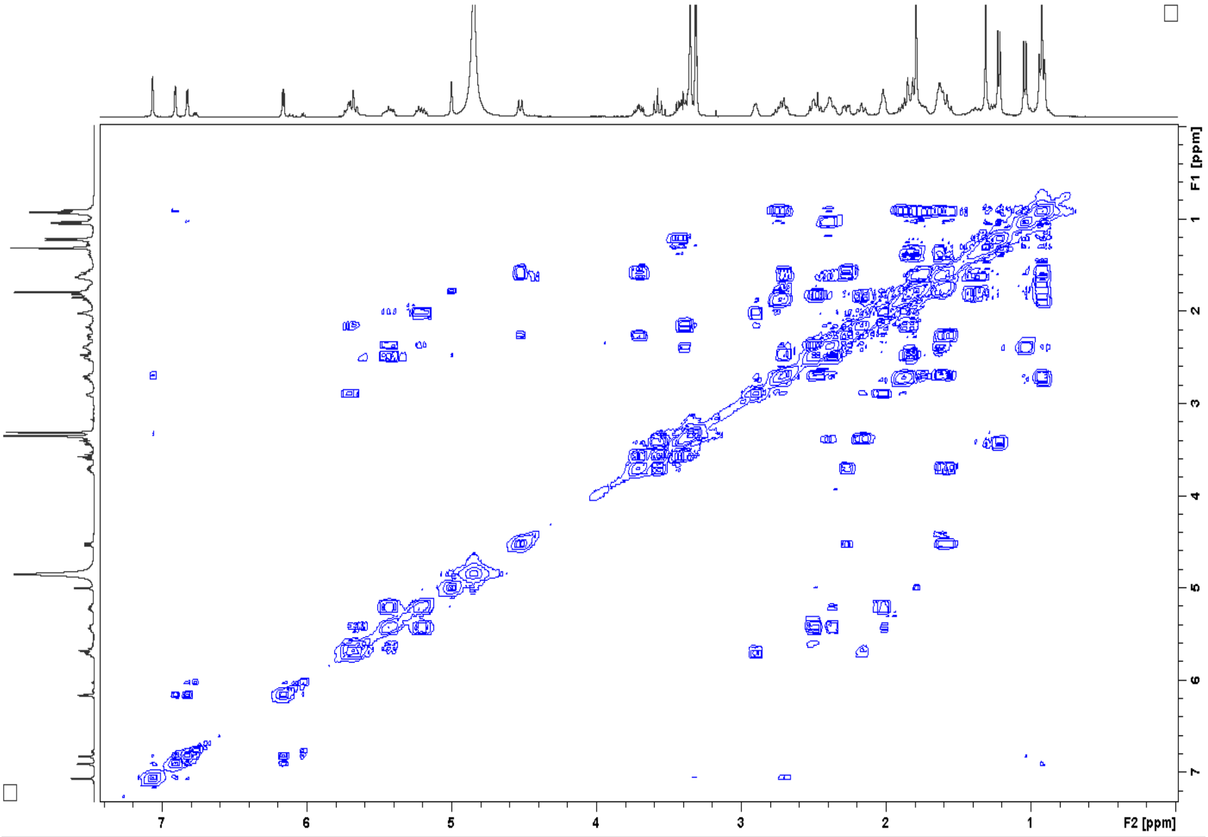

Figure S28. COSY spectrum of **8.**

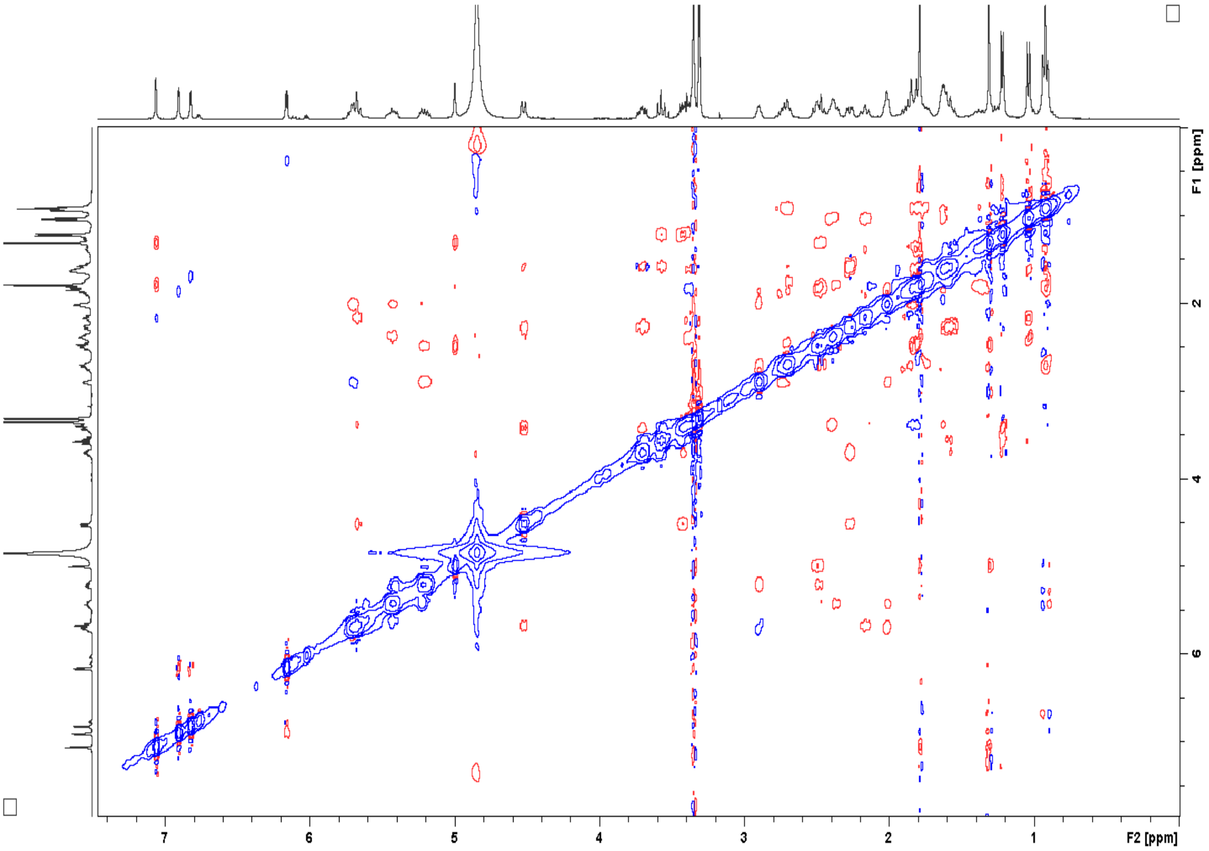

Figure S29. NOESY spectrum of **8**.

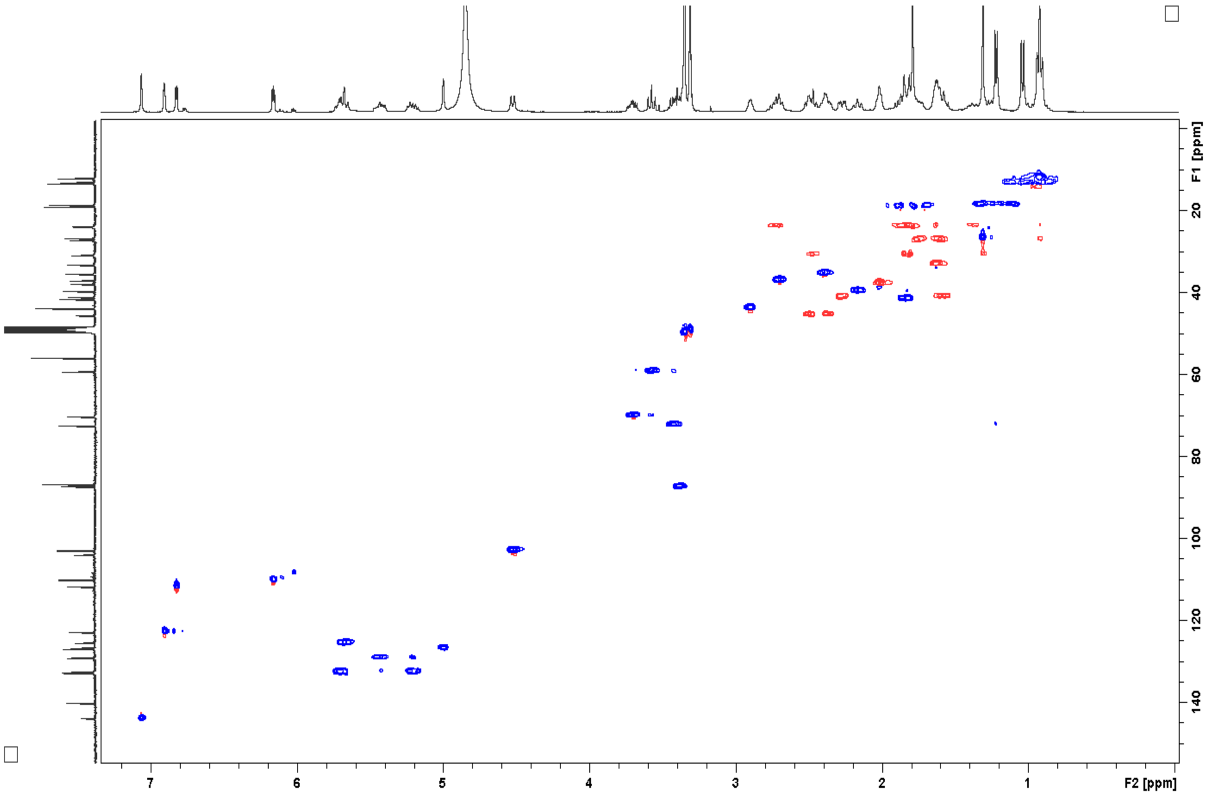

Figure S30. HSQC spectrum of **8.**

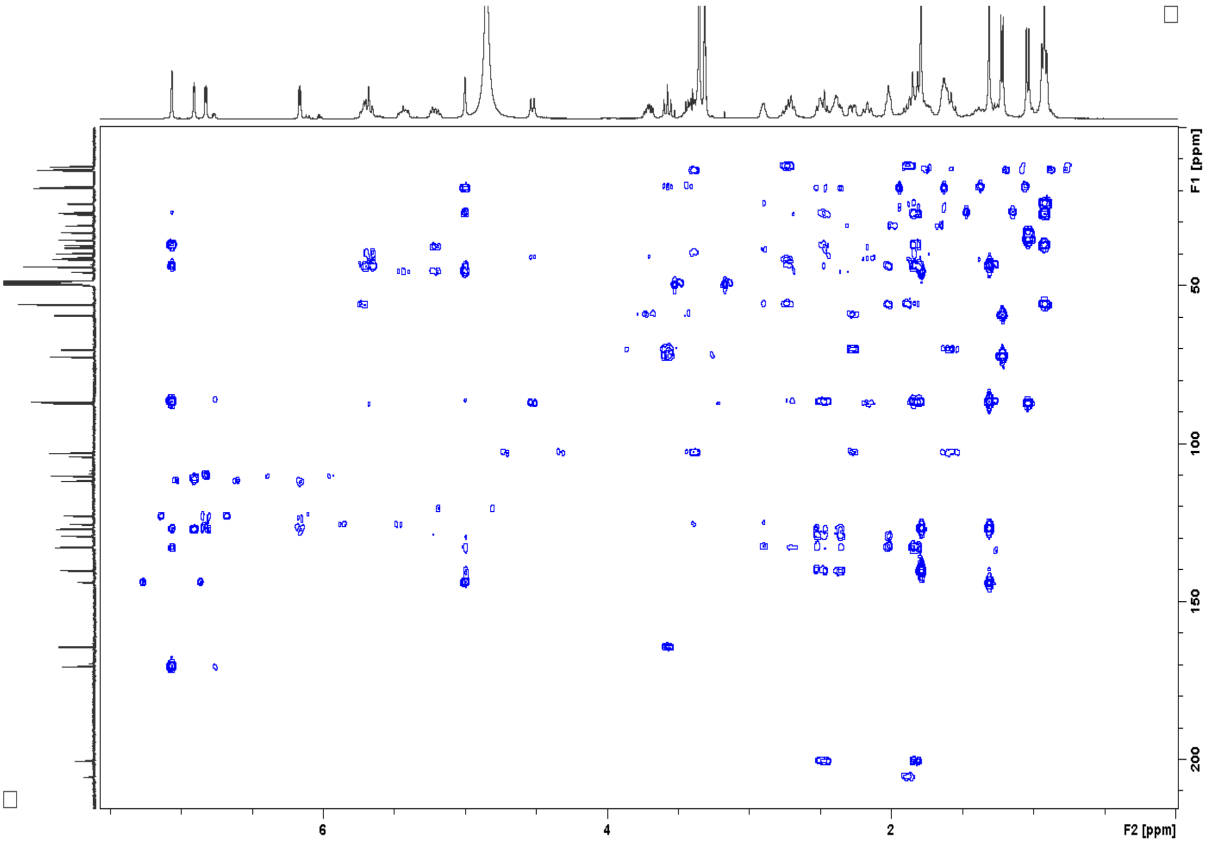

Figure S31. HMBC spectrum of **8.**

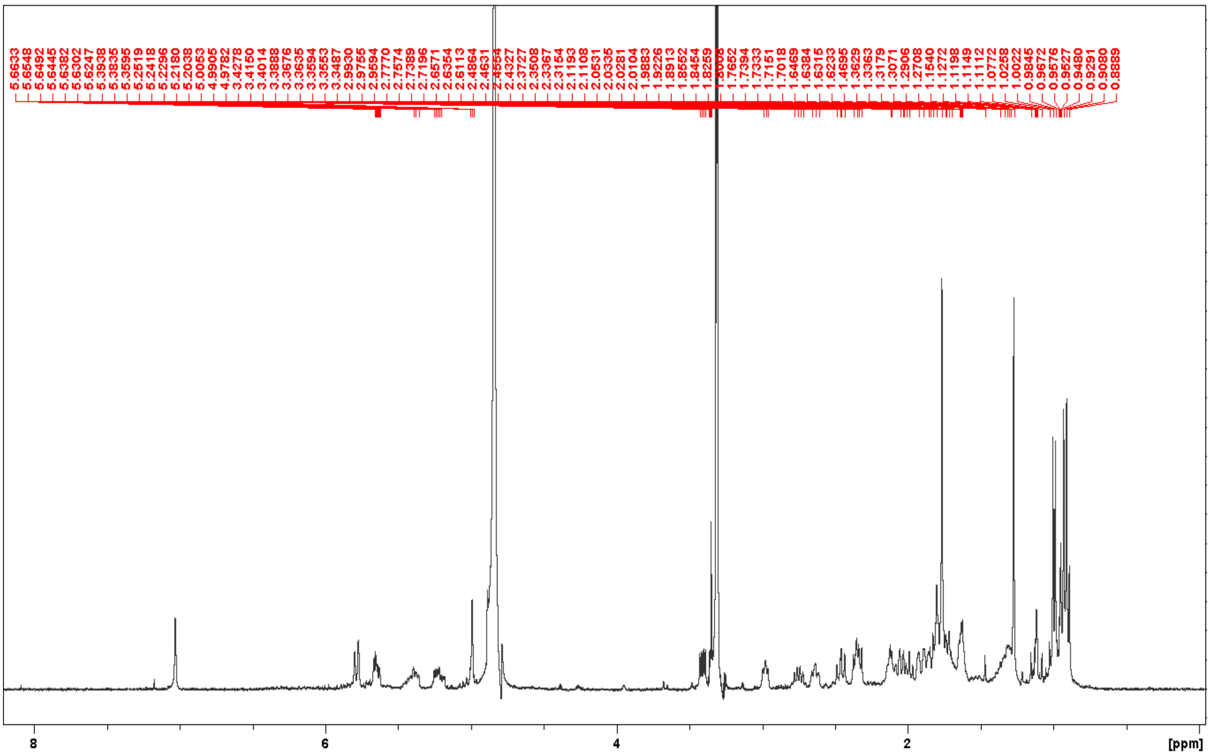

Figure S32. ^{1}H NMR spectrum (MeOH-d_4, 400 MHz) of **9.**

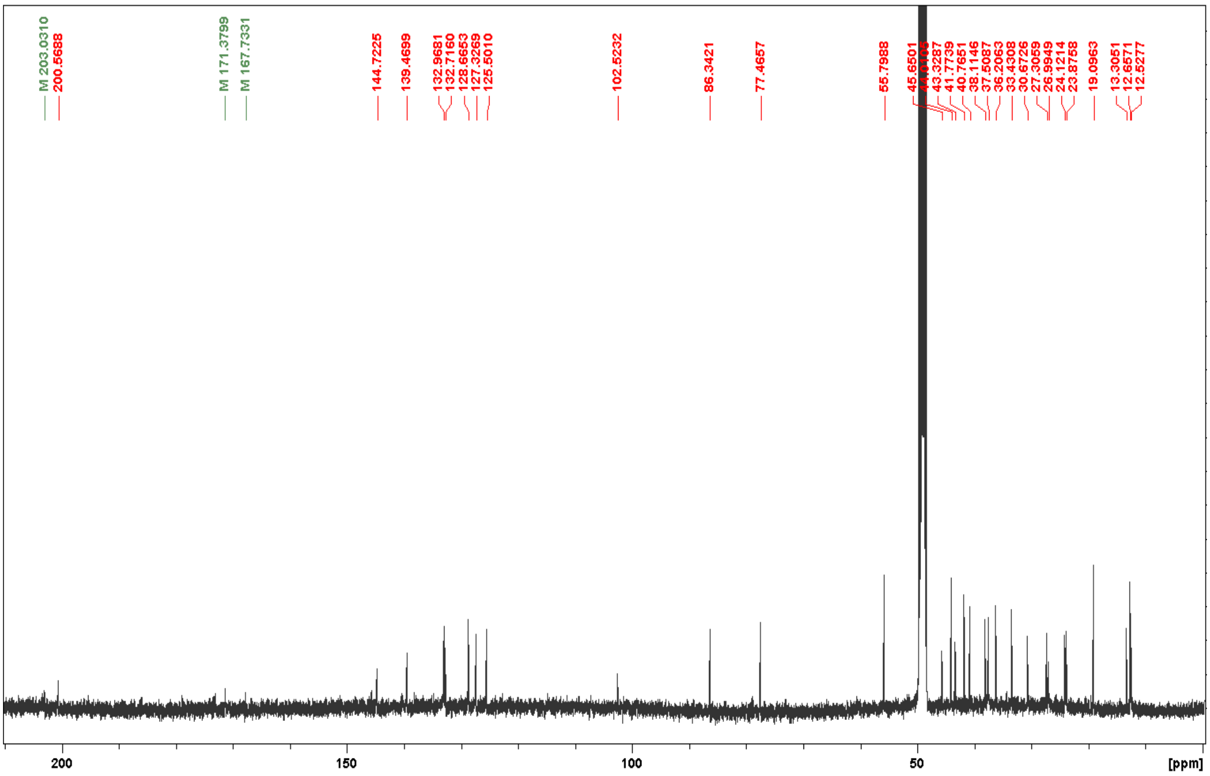

Figure S33. ^{13}C NMR spectrum (MeOH-d_4, 100 MHz) of **9.**

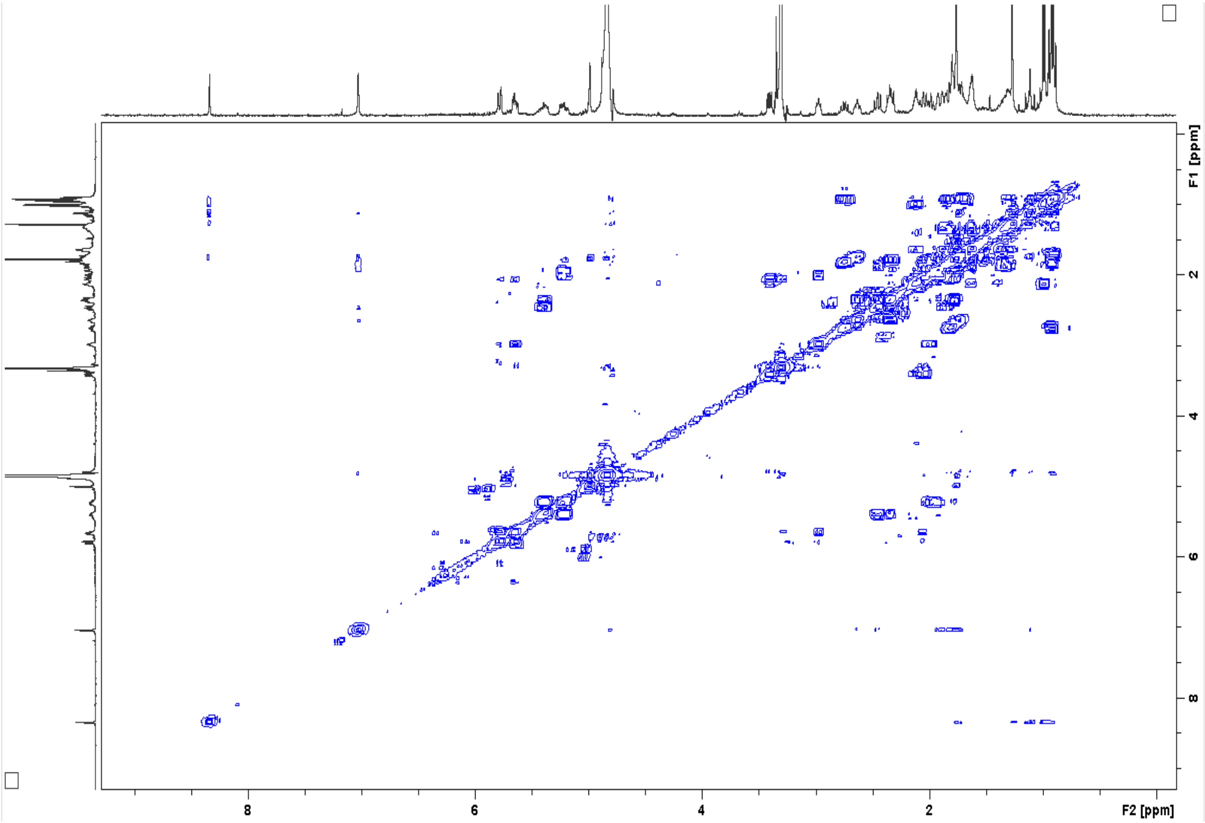

Figure S34. COSY spectrum of **9.**

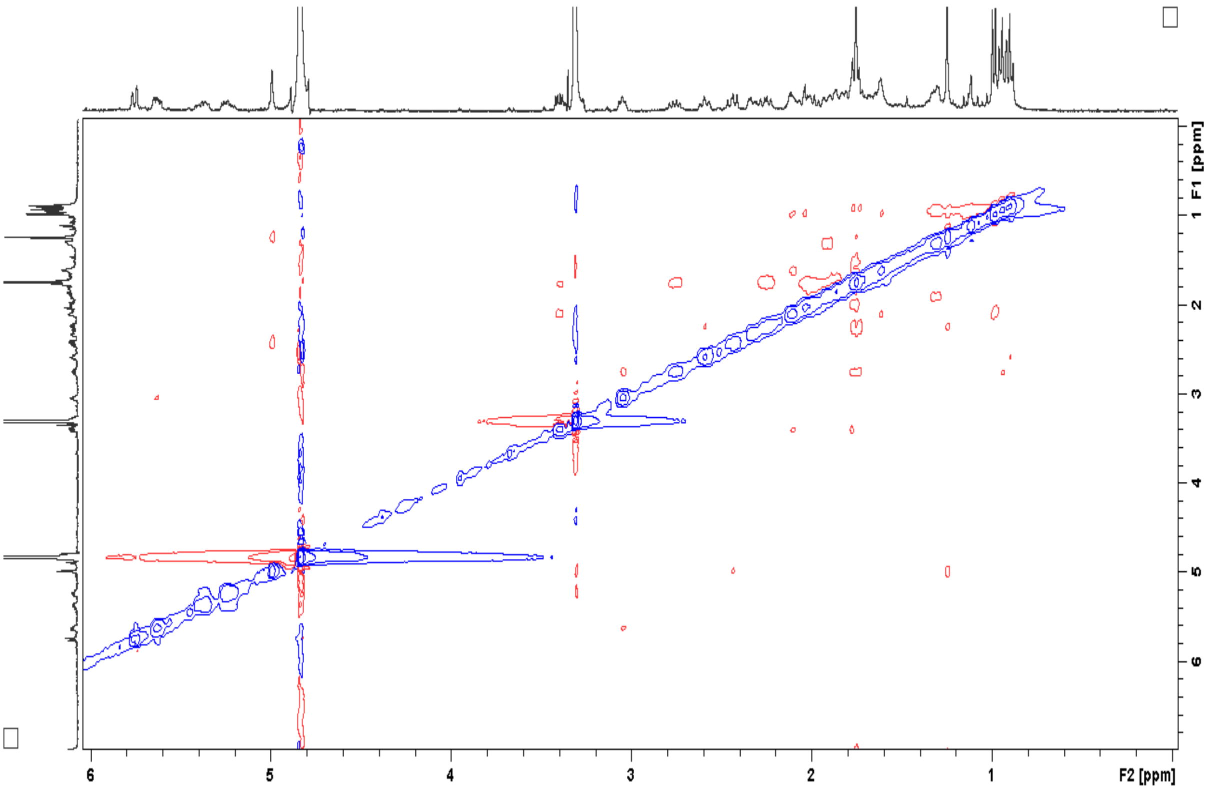

Figure S35. NOESY spectrum of **9**.

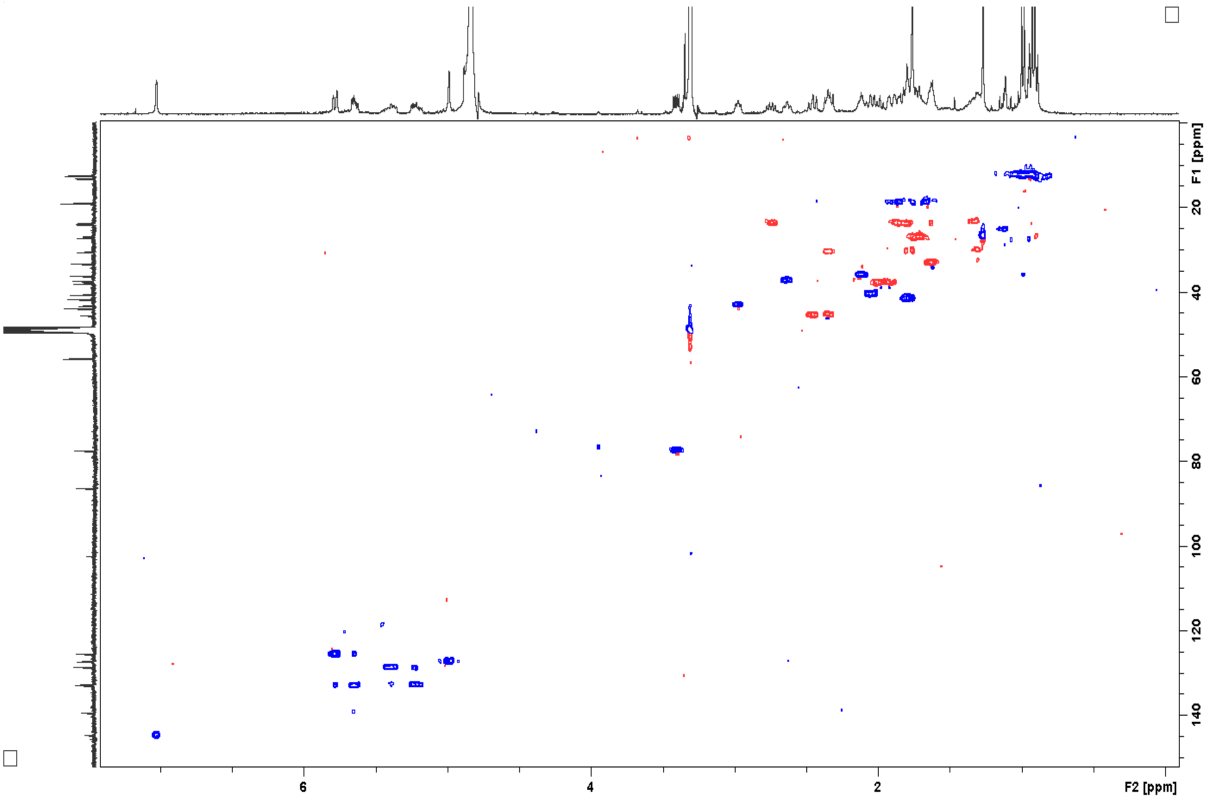

Figure S36. HSQC spectrum of **9.**

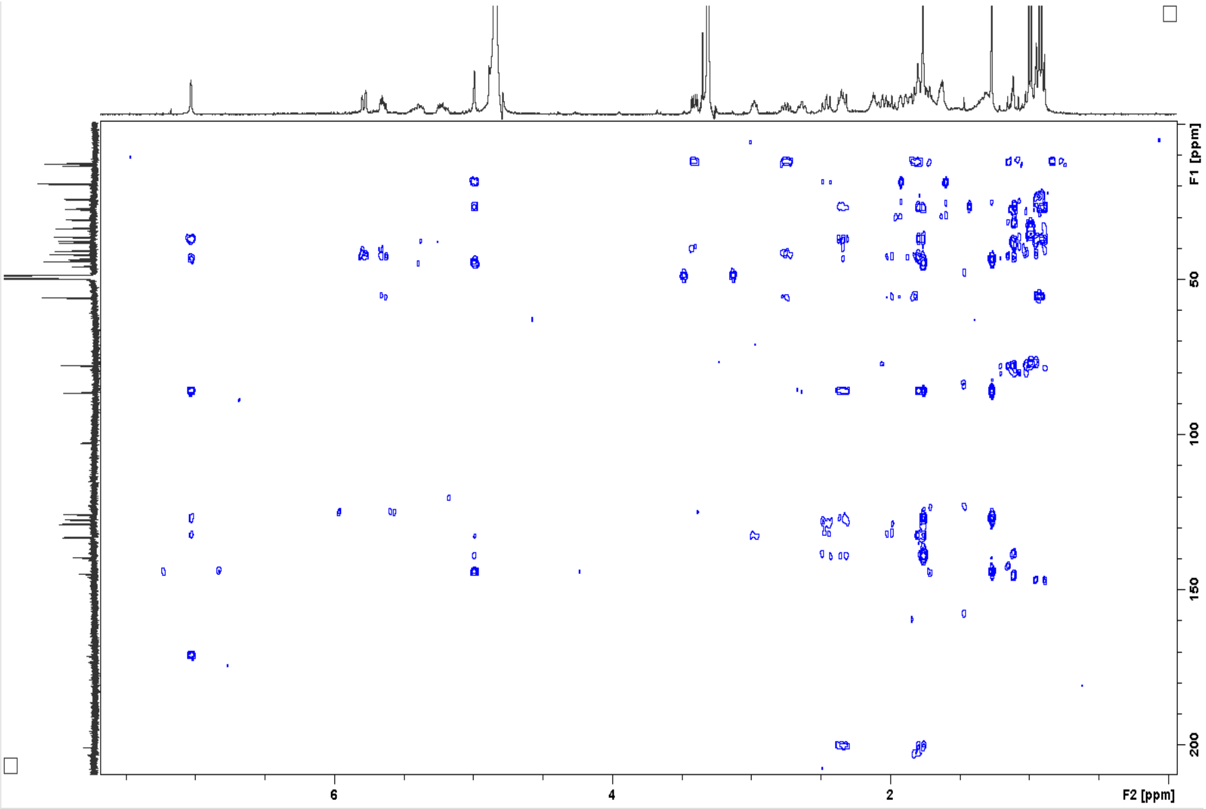

Figure S37. HMBC spectrum of **9.**

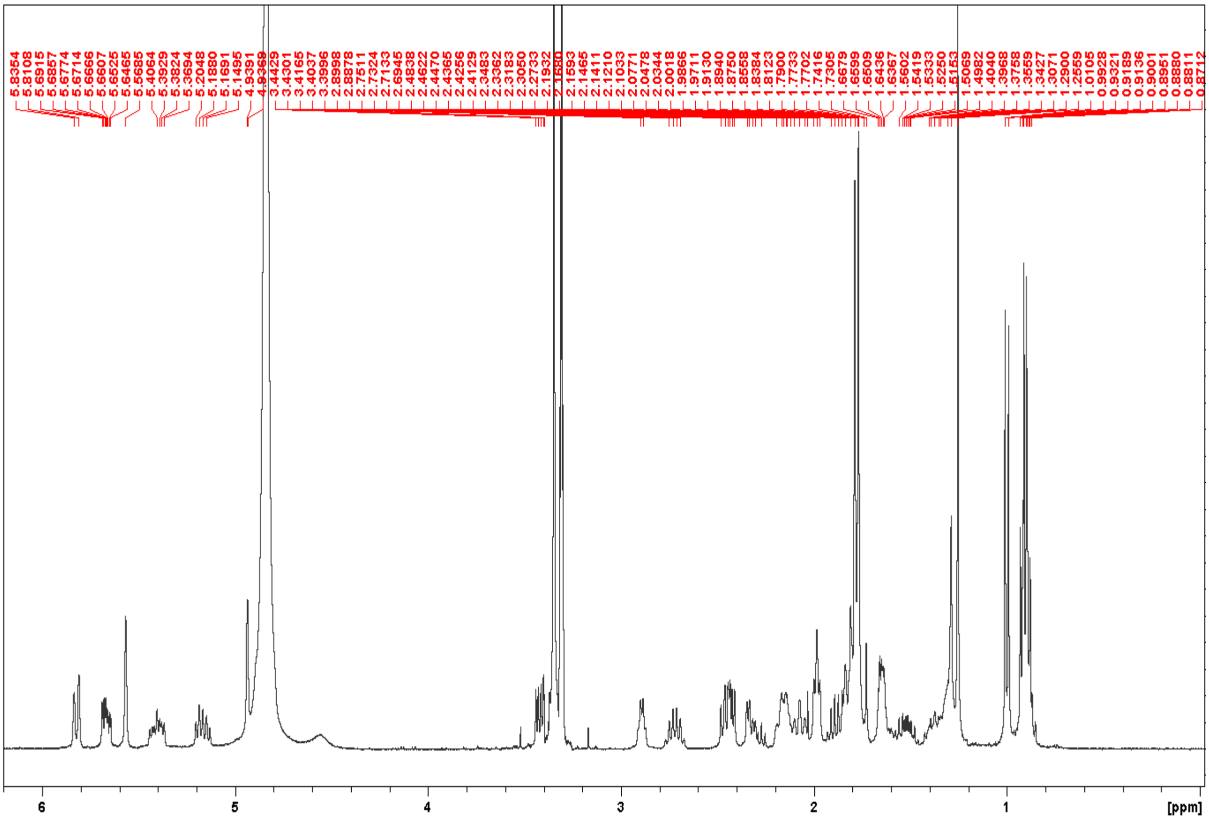

Figure S38. ^{1}H NMR spectrum (MeOH-d_4, 400 MHz) of **10.**

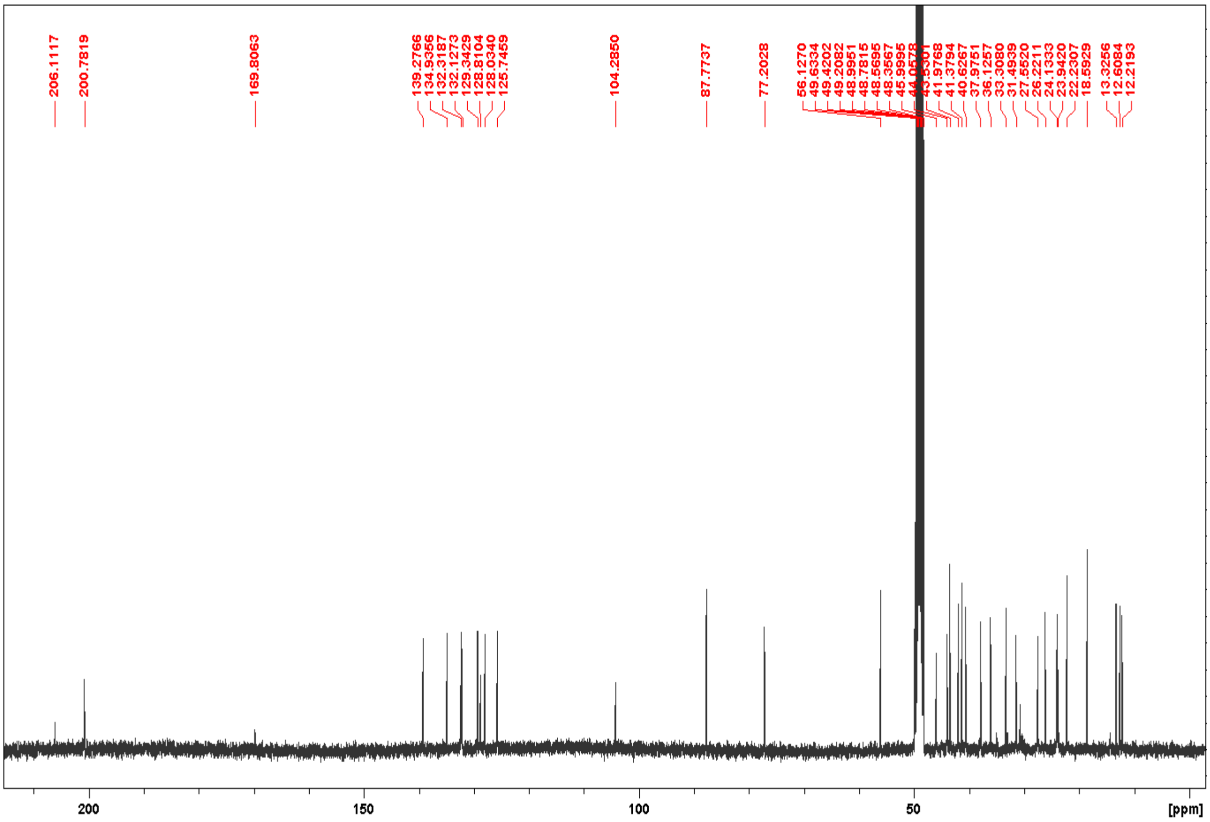

Figure S39. ^{13}C NMR spectrum (MeOH-d_4, 100 MHz) of **10.**

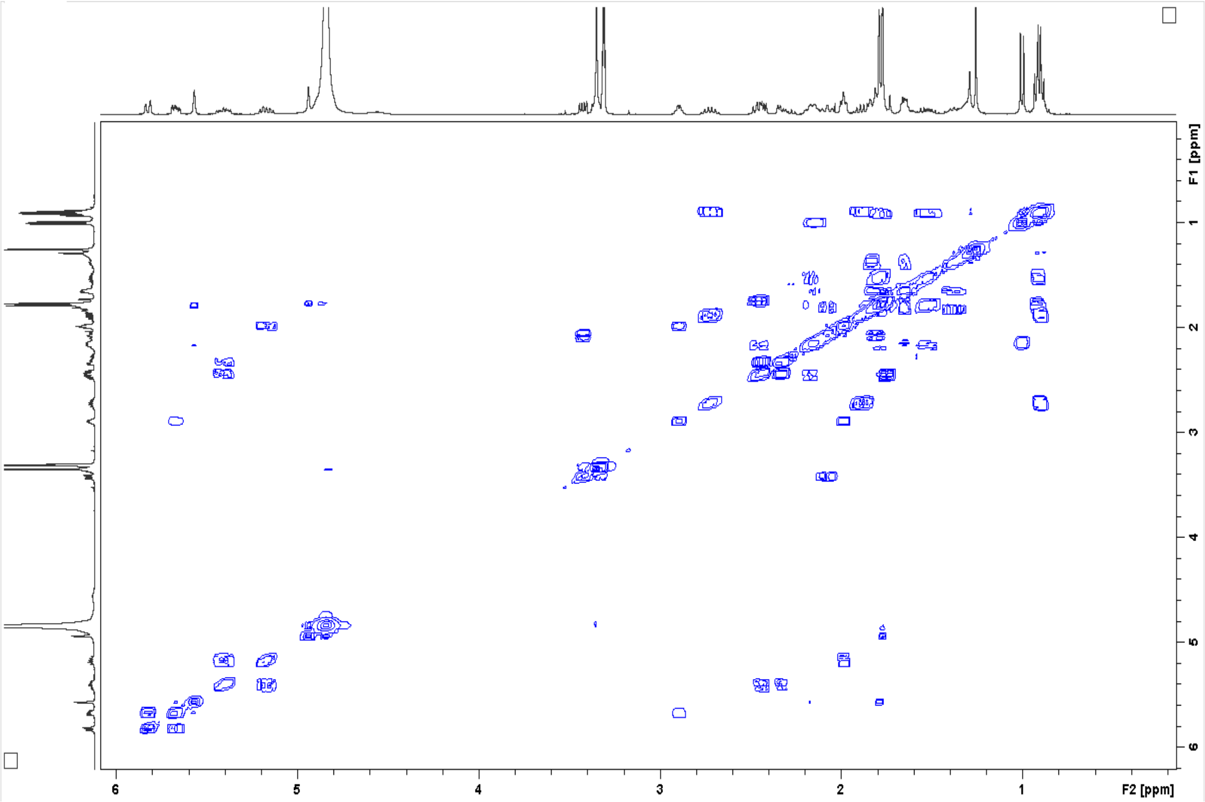

Figure S40. COSY spectrum of **10.**

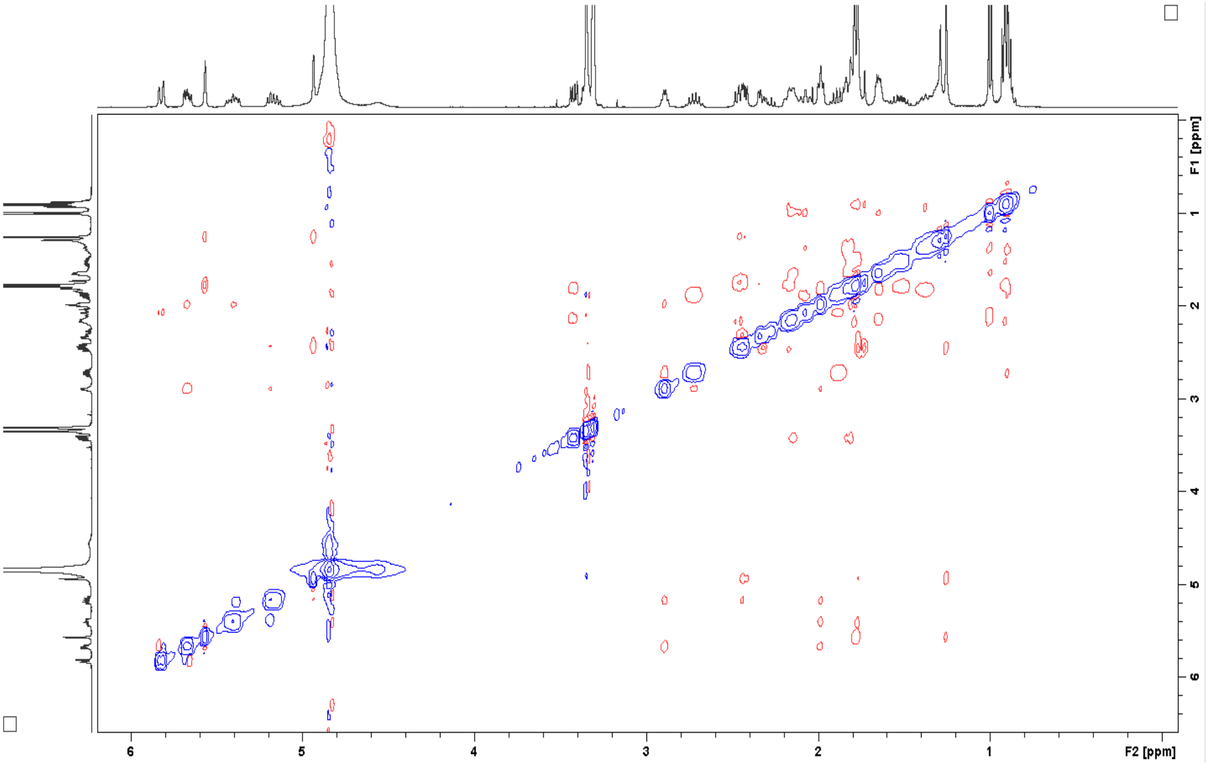

Figure S41. NOESY spectrum of **10**.

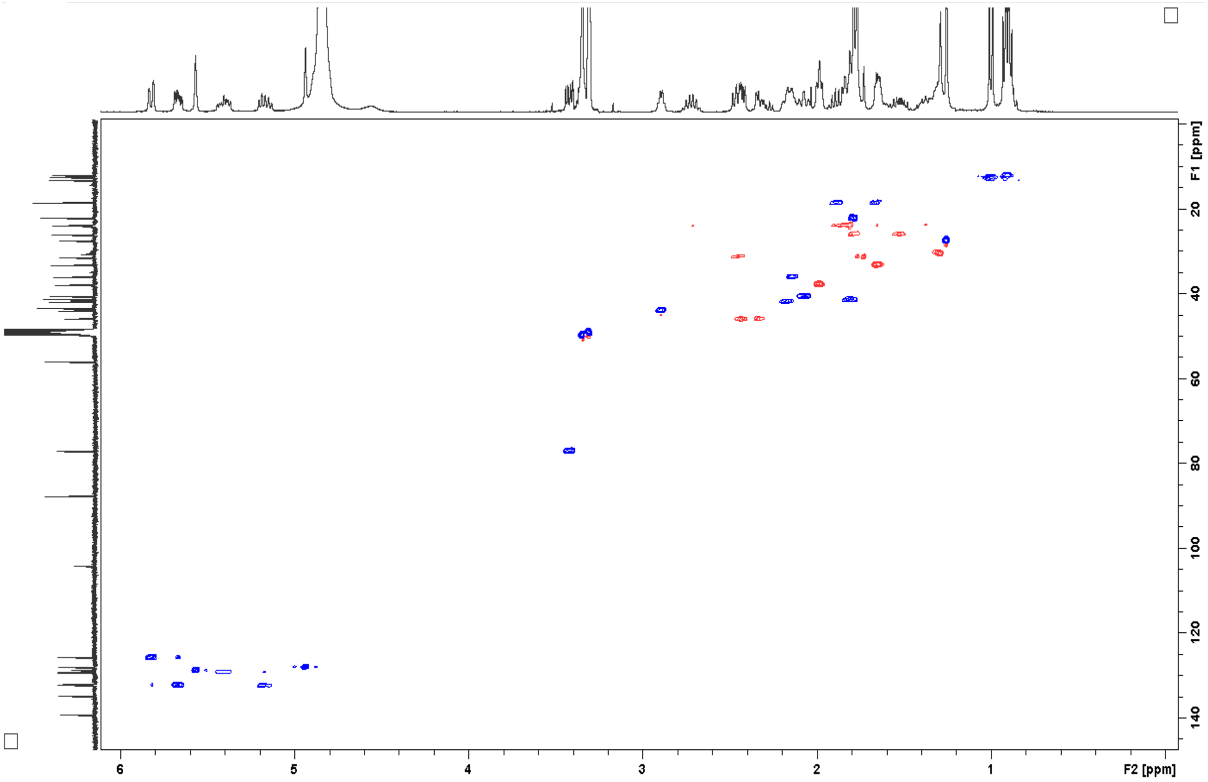

Figure S42. HSQC spectrum of **10.**

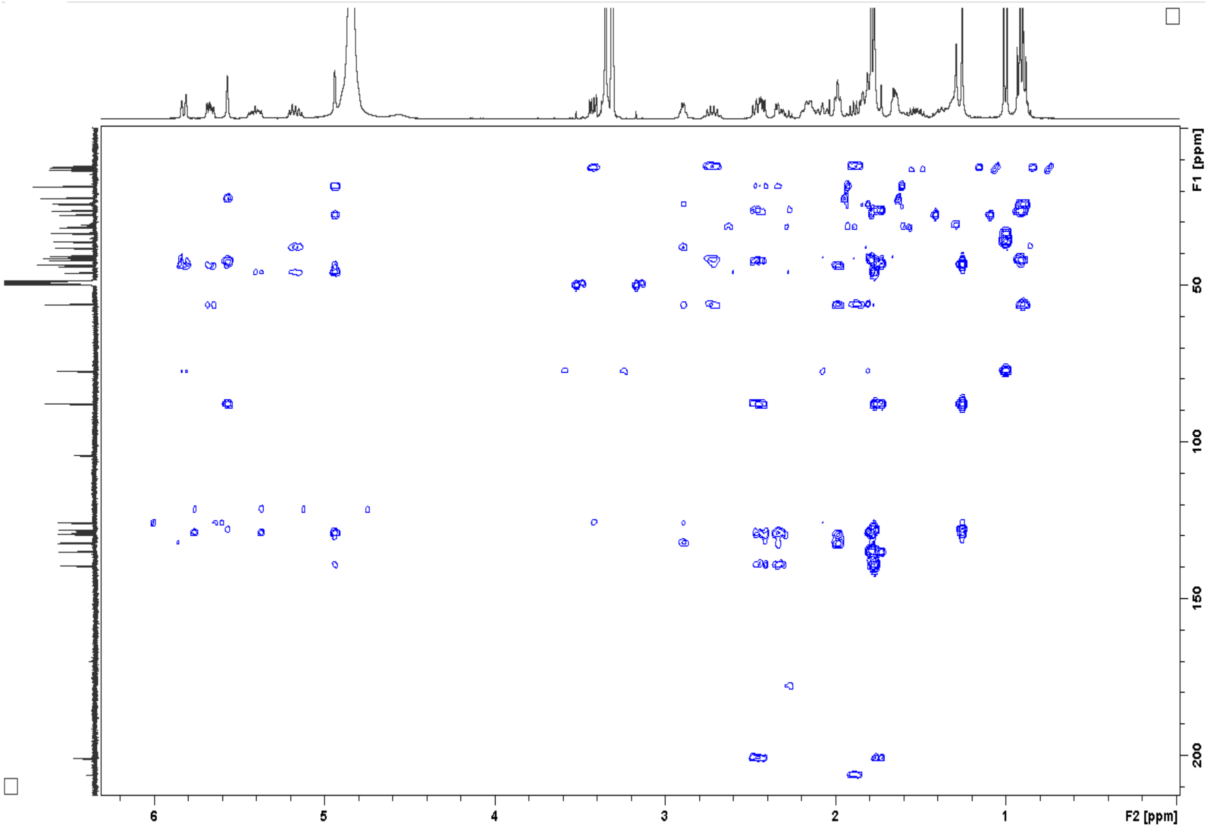

Figure S43. HMBC spectrum of **10.**

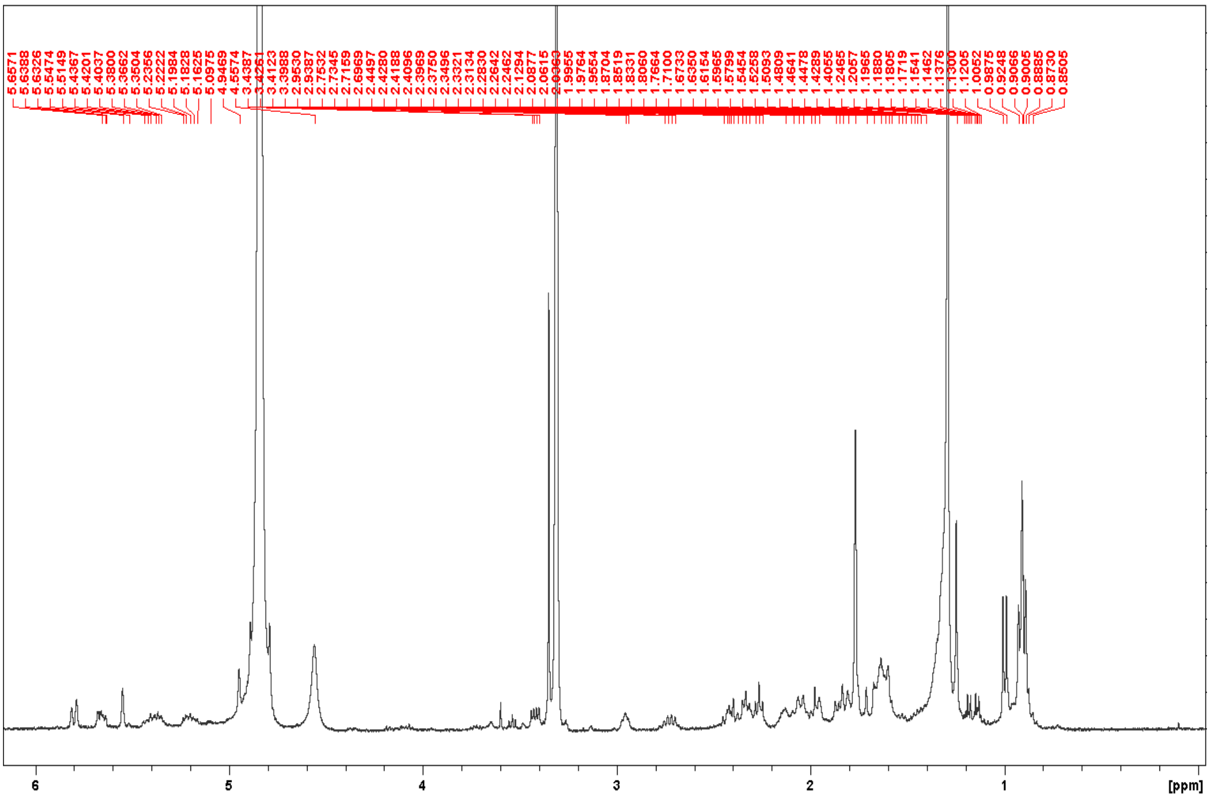

Figure S44. ^{1}H NMR spectrum (MeOH-d_4, 400 MHz) of **11.**

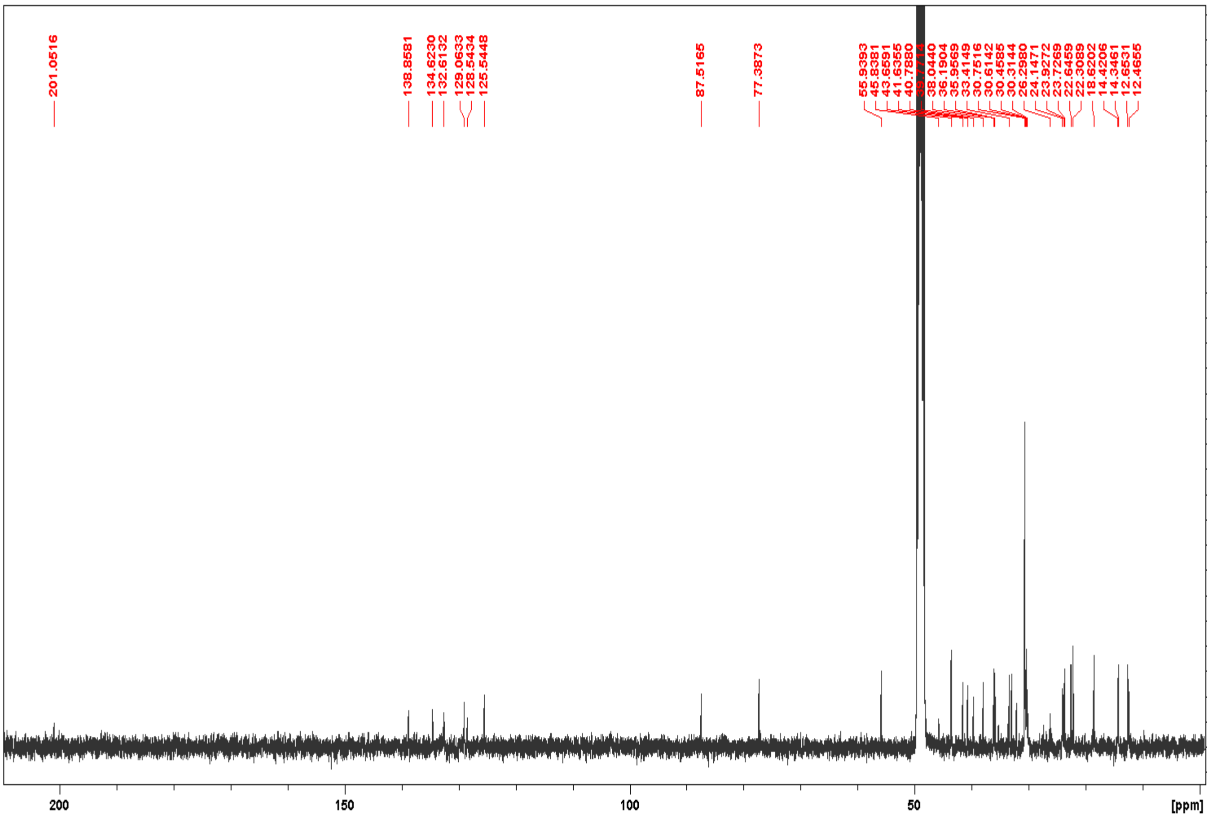

Figure S45. ^{13}C NMR spectrum (MeOH-d_4, 100 MHz) of **11.**

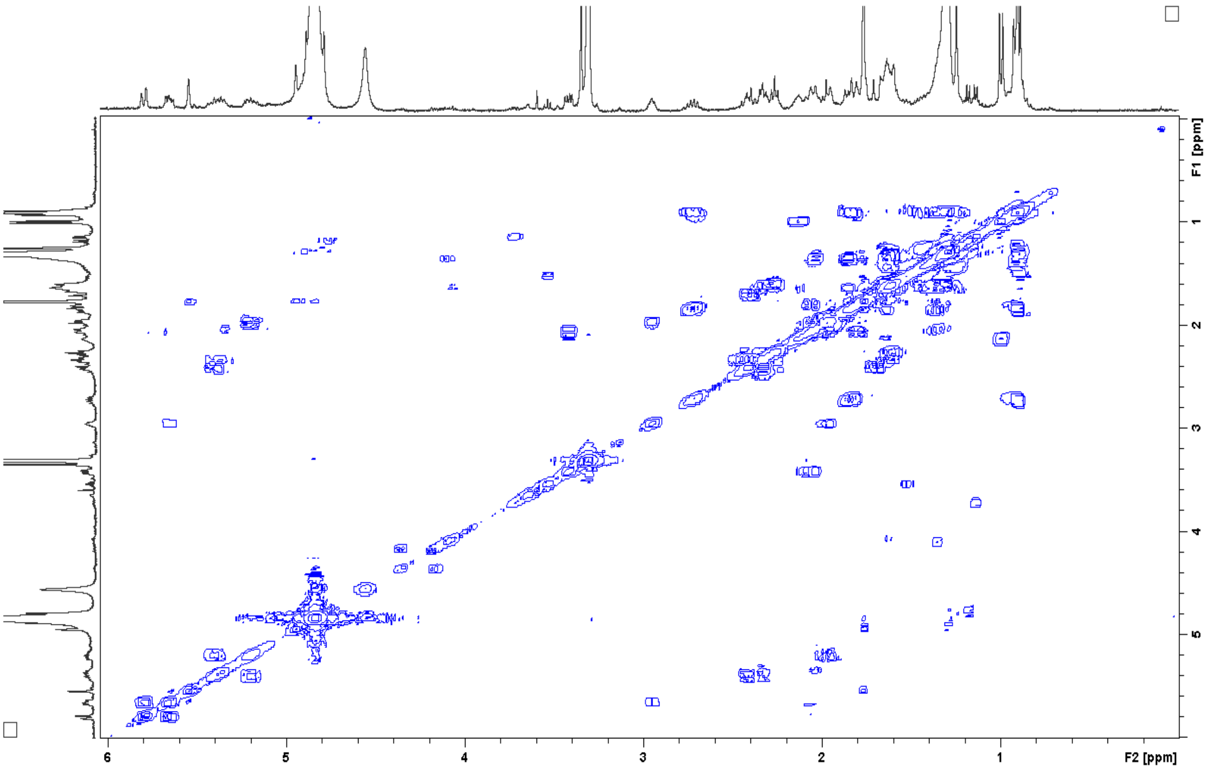

Figure S46. COSY spectrum of **11.**

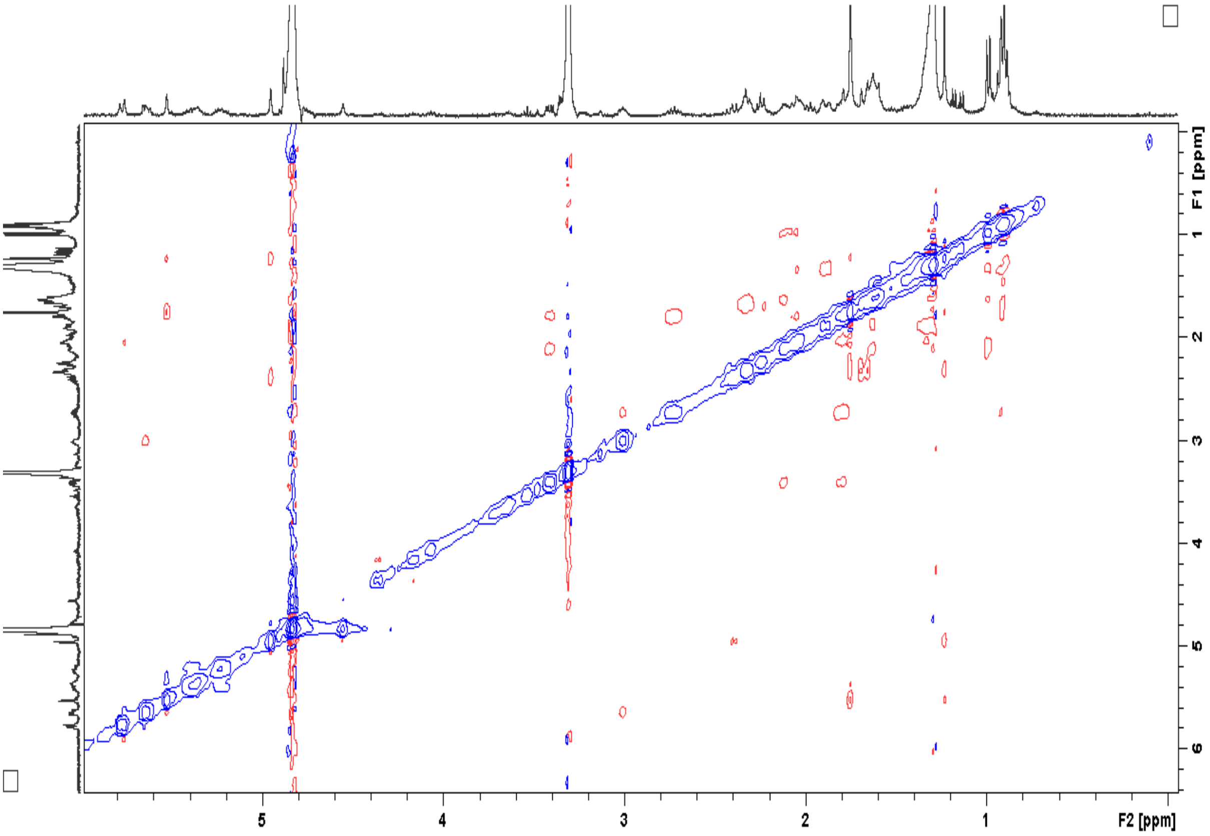

Figure S47. NOESY spectrum of **11**.

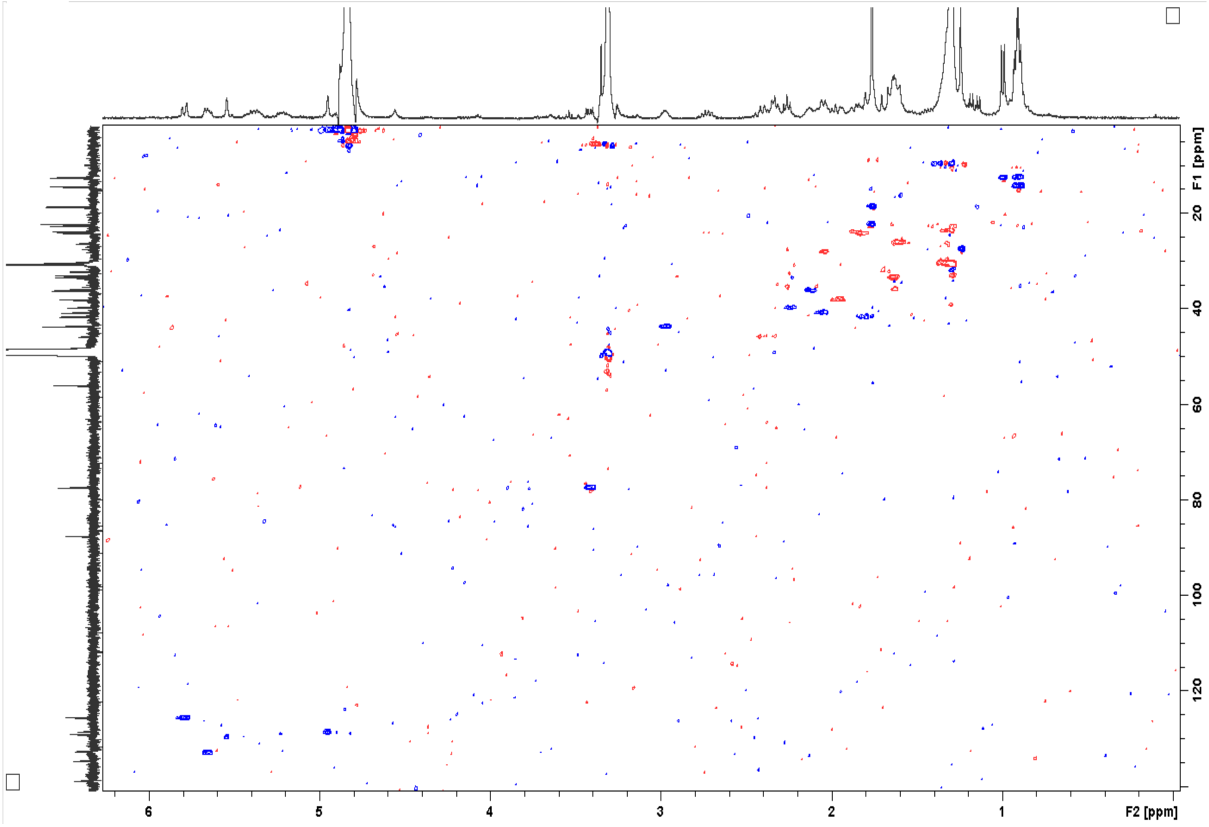

Figure S48. HSQC spectrum of **11.**

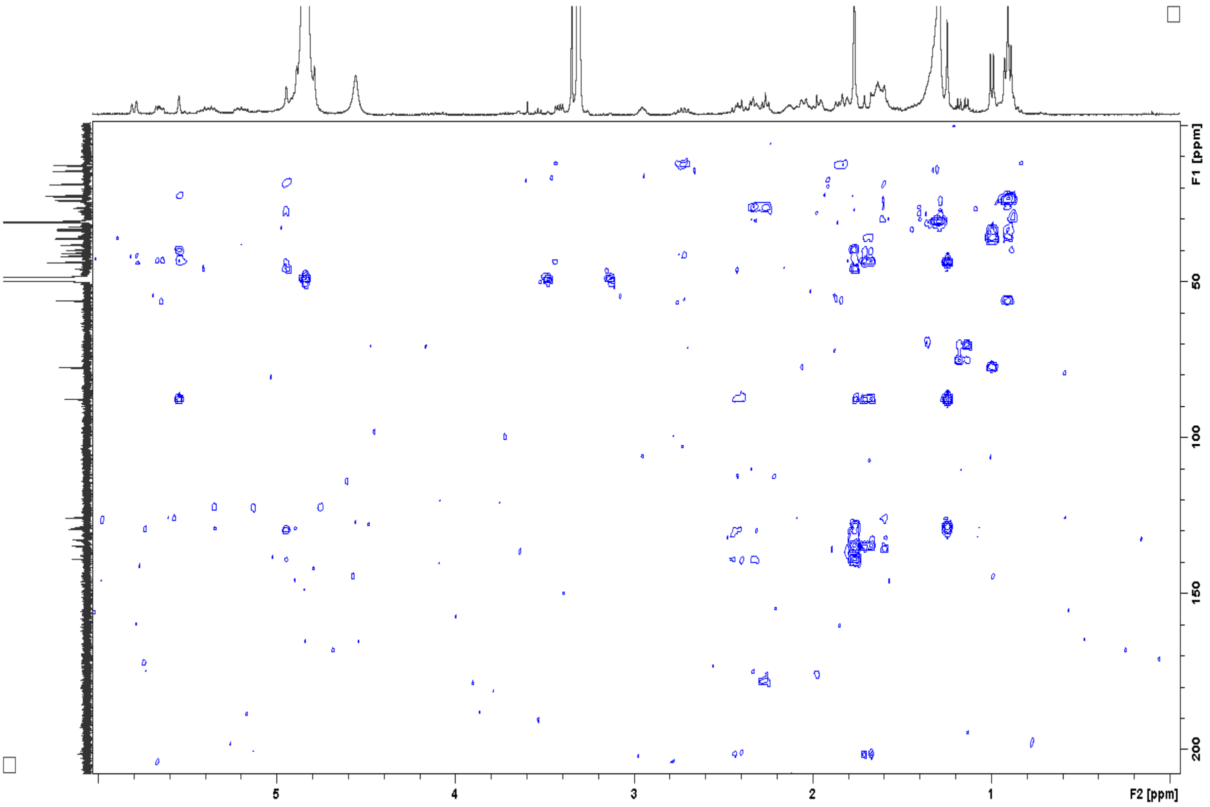

Figure S49. HMBC spectrum of **11.**

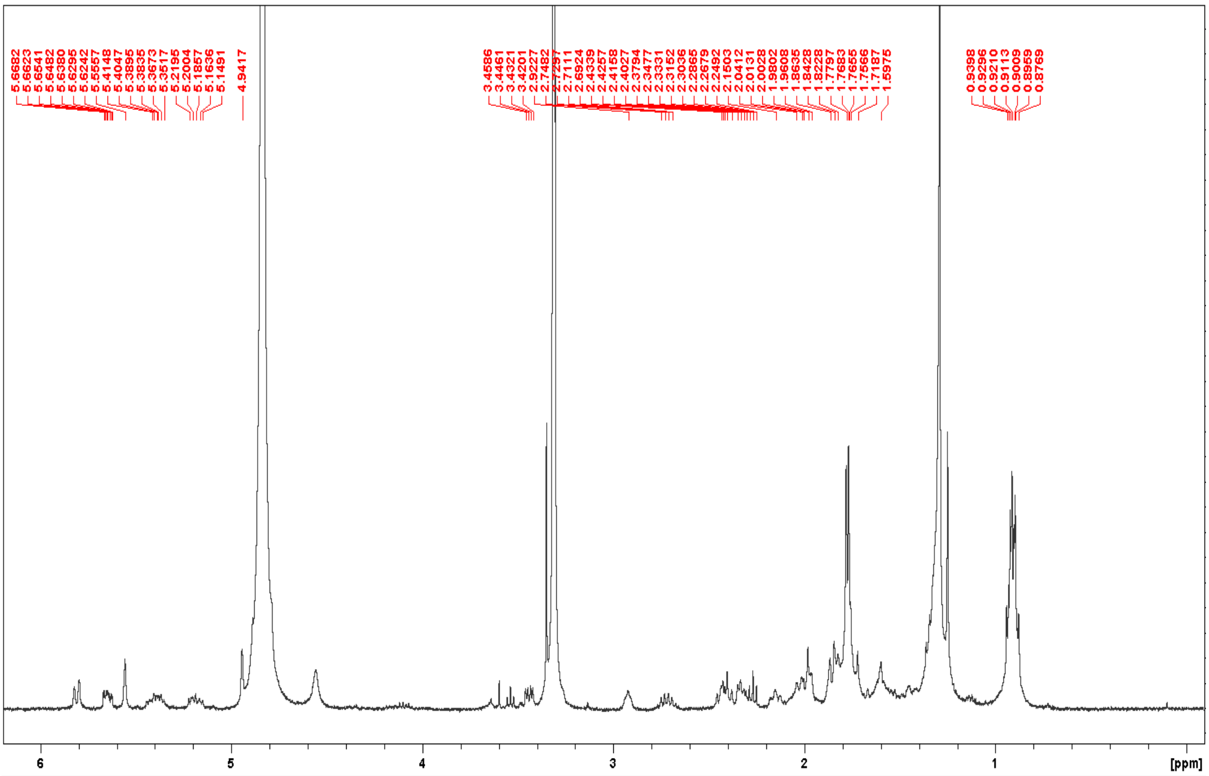

Figure S50. ^{1}H NMR spectrum (MeOH-d_4, 400 MHz) of **12.**

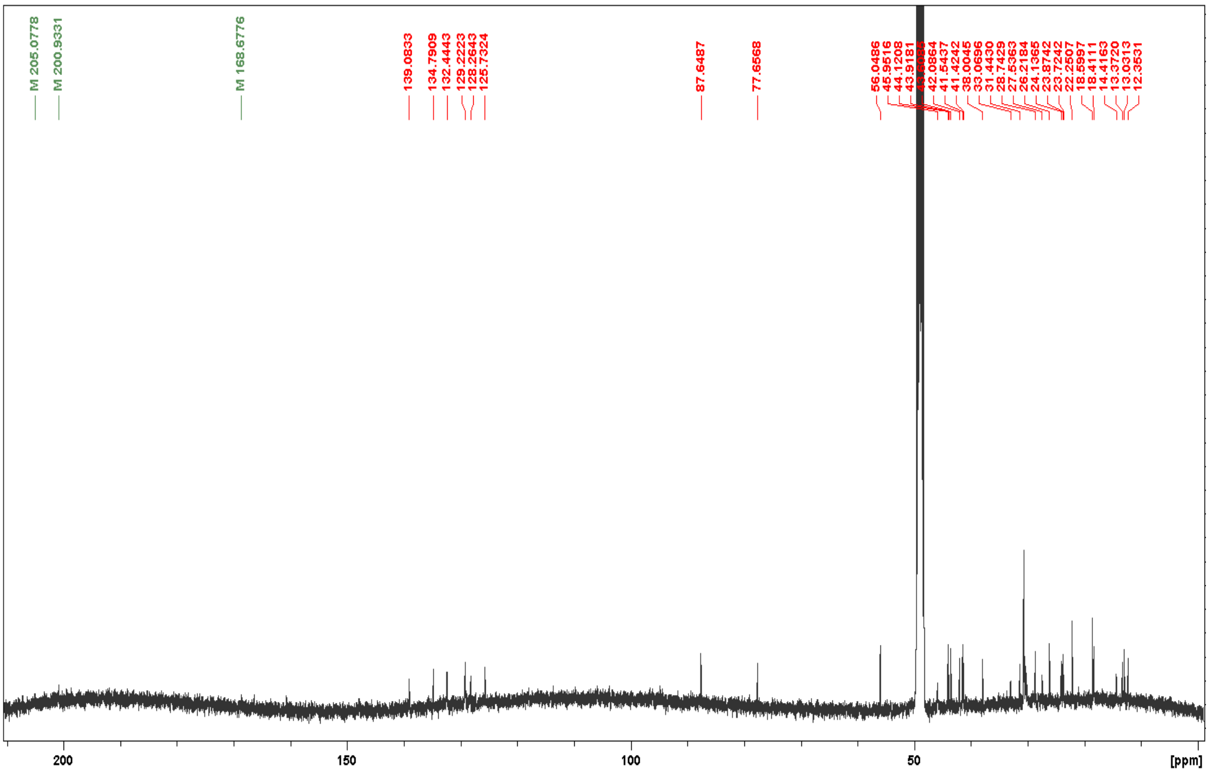

Figure S51. ^{13}C NMR spectrum (MeOH-d_4, 100 MHz) of **12.**

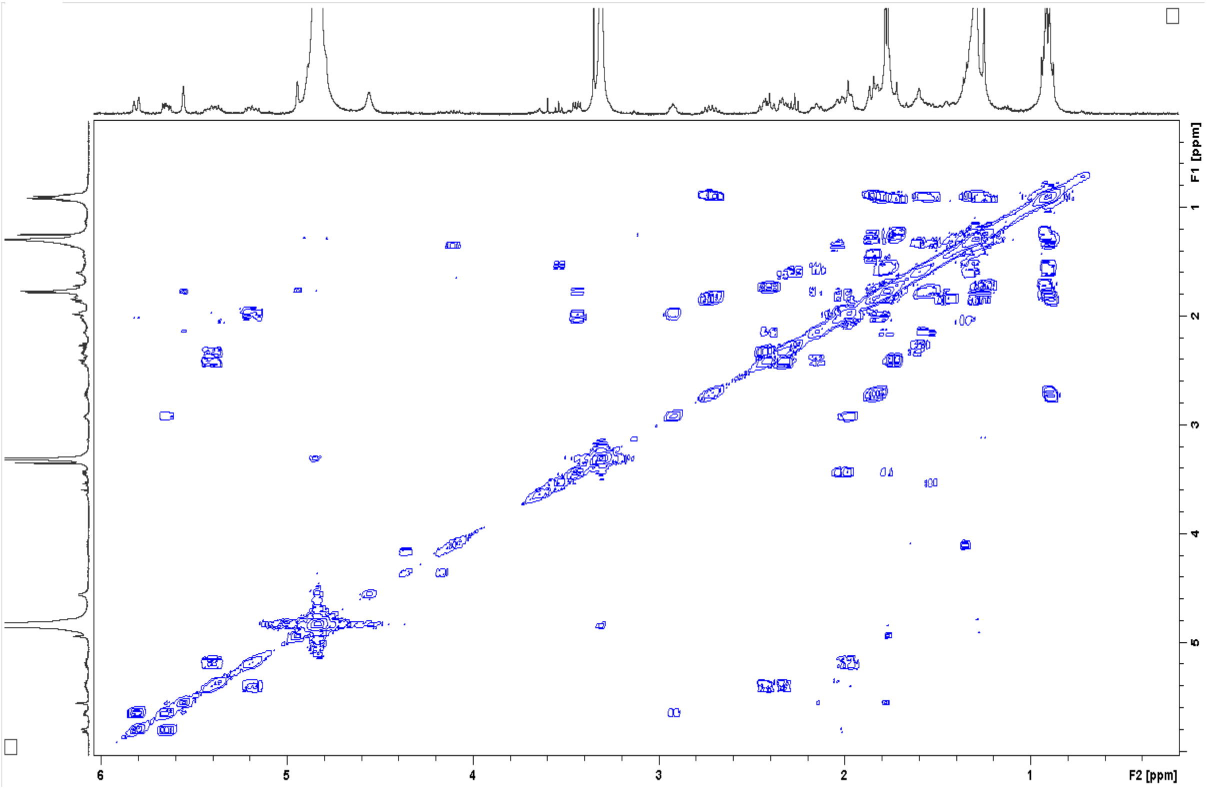

Figure S52. COSY spectrum of **12.**

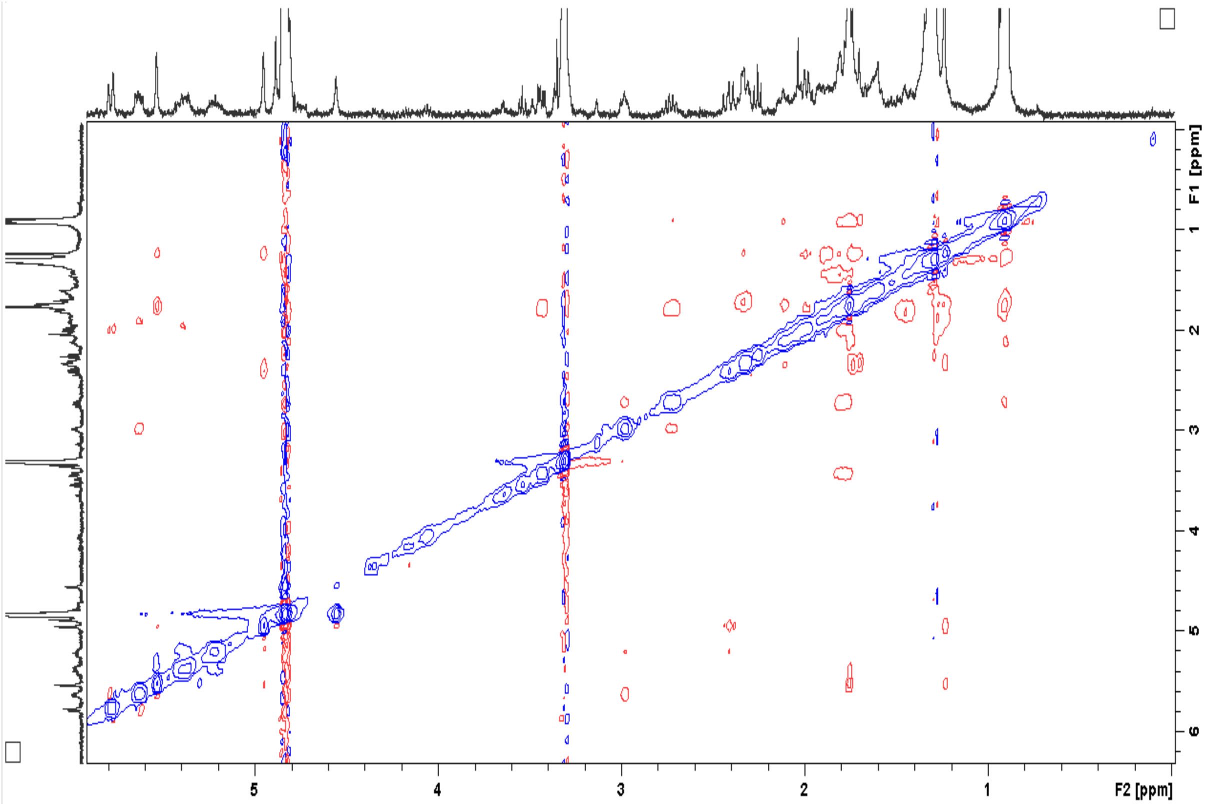

Figure S53. NOESY spectrum of **12**.

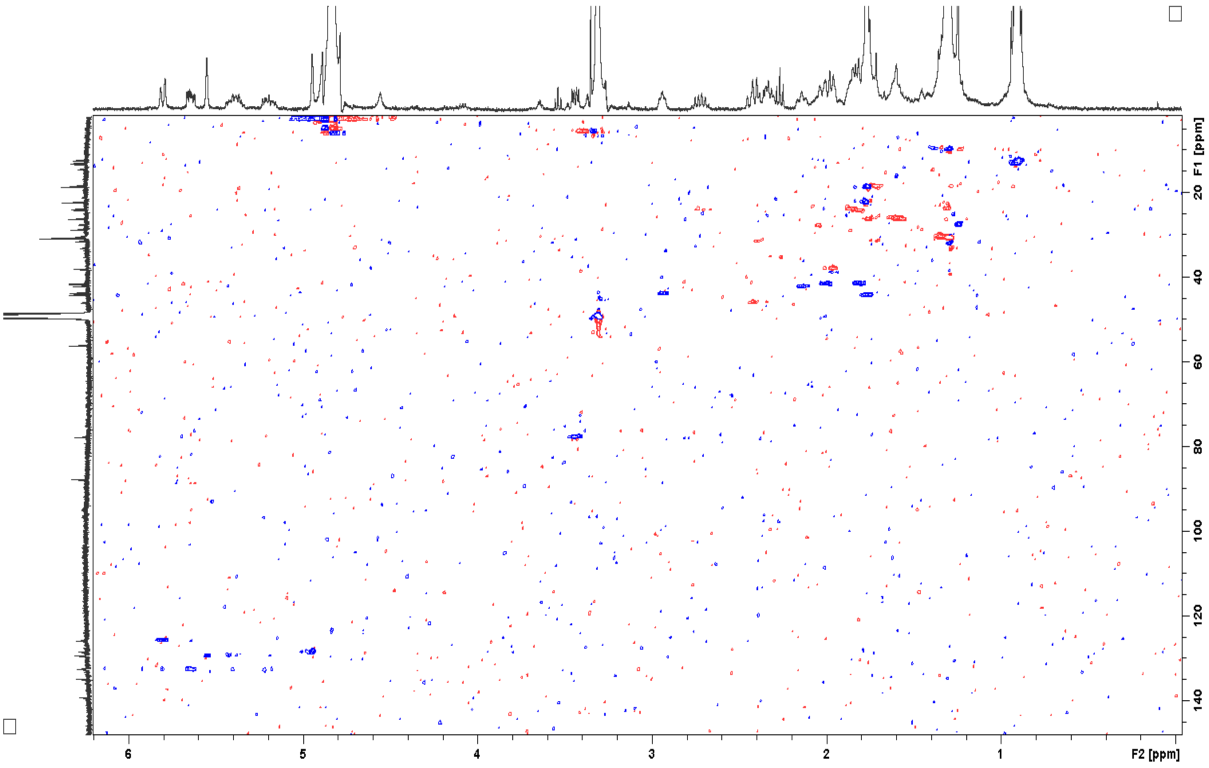

Figure S54. HSQC spectrum of **12.**

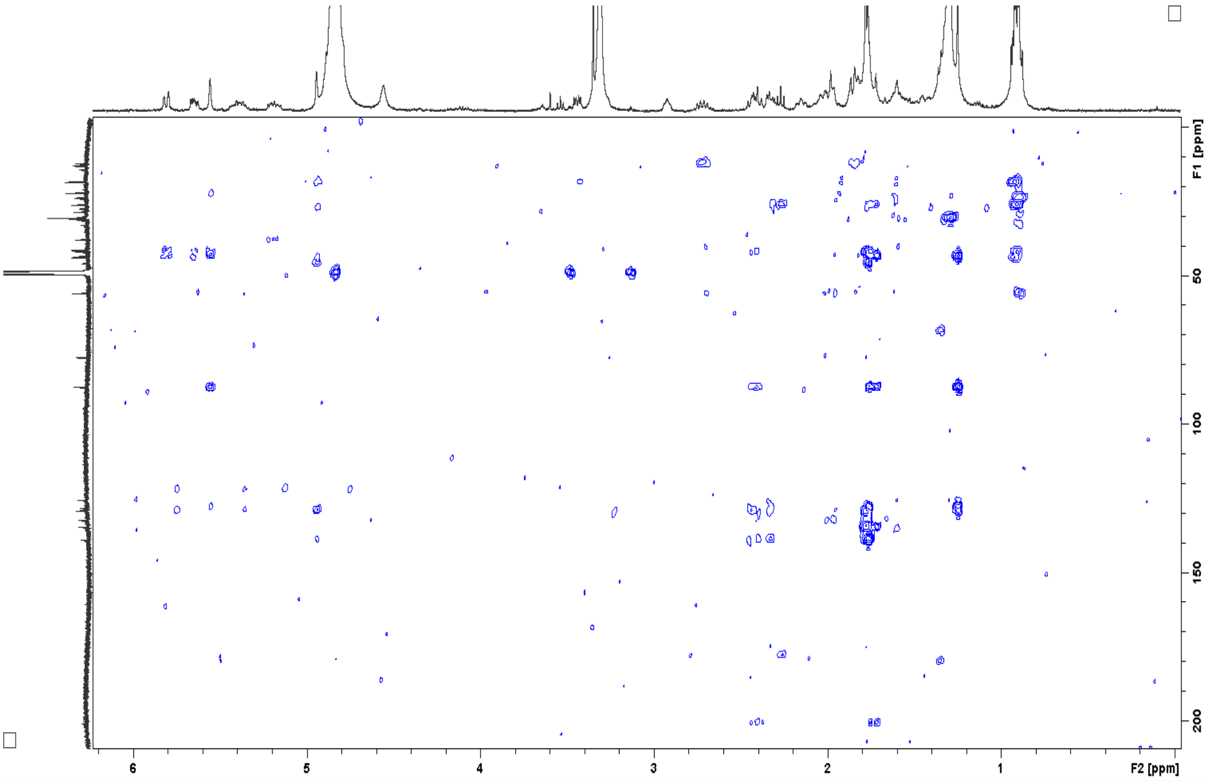

Figure S55. HMBC spectrum of **12.**

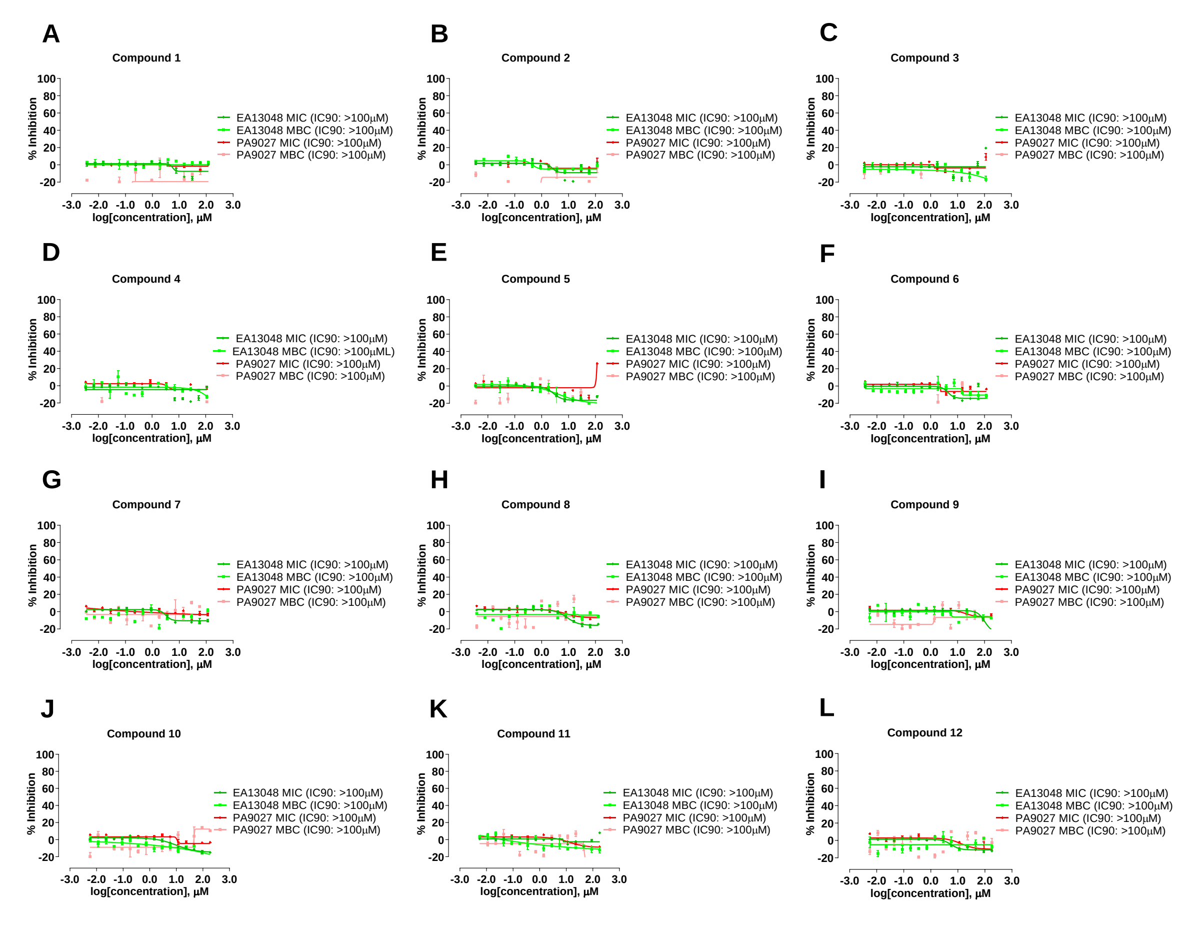

Figure S56. Inhibitory effect dose response curve against *Klebsiella aerogenes* (ATCC® 13048™) and *Pseudomonas aeruginosa* (ATCC® 9027™). **A)** Compound **1**, **B)** Compound **2**, **C)** Compound **3**, **D)** Compound **4**, **E)** Compound **5**, **F)** Compound **6**, **G)** Compound **7**, **H)** Compound **8**, **I)** Compound **9**, **J)** Compound **10**, **K)** Compound **11**, **L)** Compound **12**.

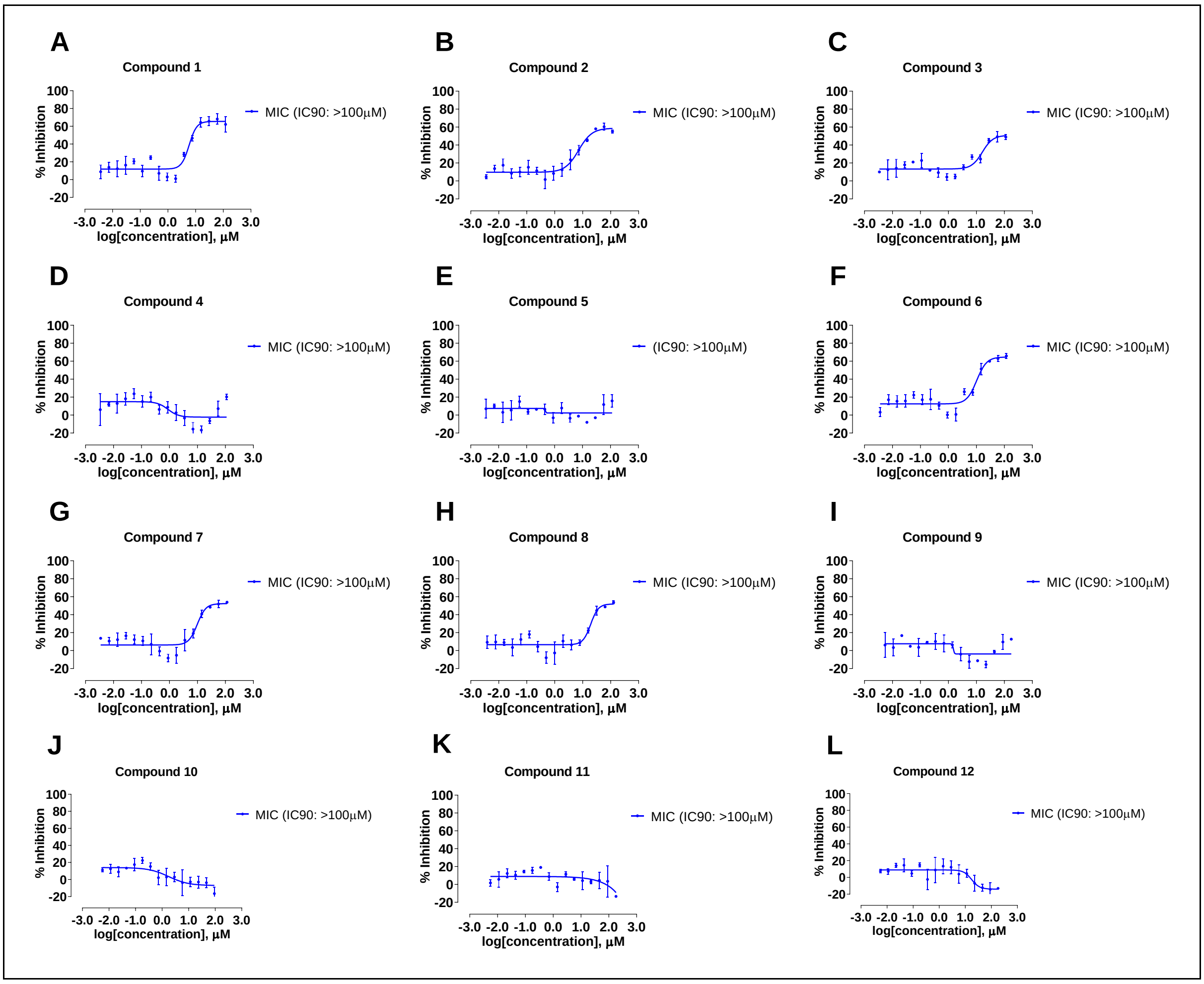

Figure S57. Inhibitory effect dose response curve against *A. fumigatus* (ATCC® 46645™). **A)** Compound **1**, **B)** Compound **2**, **C)** Compound **3**, **D)** Compound **4**, **E)** Compound **5**, **F)** Compound **6**, **G)** Compound **7**, **H)** Compound **8**, **I)** Compound **9**, **J)** Compound **10**, **K)** Compound **11**, **L)** Compound **12**.

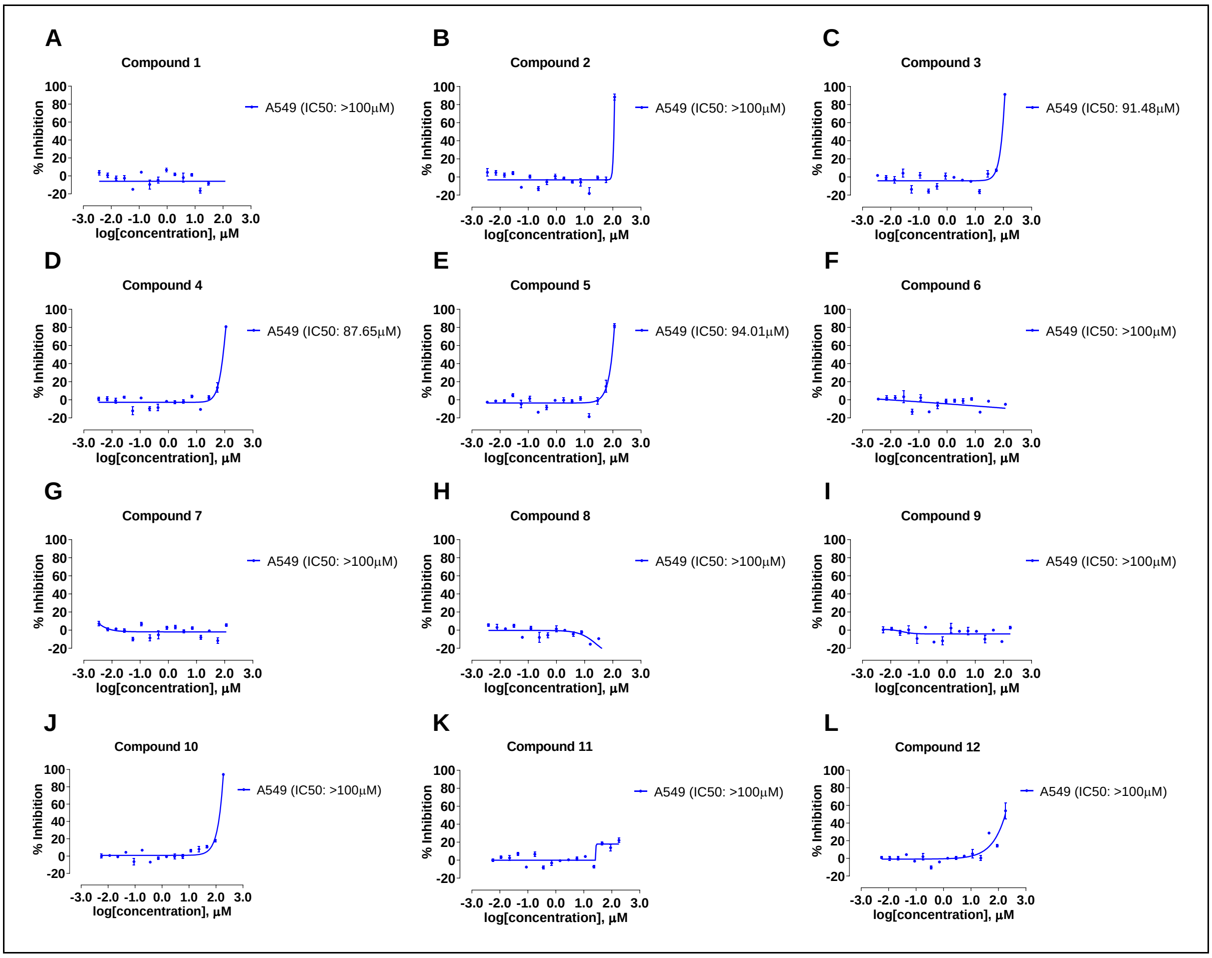

Figure S58. Inhibitory effect dose response curve against A549 Human lung carcinoma cells (ATCC® CCL-185™). **A)** Compound **1**, **B)** Compound **2**, **C)** Compound **3**, **D)** Compound **4**, **E)** Compound **5**, **F)** Compound **6**, **G)** Compound **7**, **H)** Compound **8**, **I)** Compound **9**, **J)** Compound **10**, **K)** Compound **11**, **L)** Compound **12**.
